# Coronavirus disease (COVID-19) pandemic: an overview of systematic reviews

**DOI:** 10.1186/s12879-021-06214-4

**Published:** 2021-06-04

**Authors:** Israel Júnior Borges do Nascimento, Dónal P. O’Mathúna, Thilo Caspar von Groote, Hebatullah Mohamed Abdulazeem, Ishanka Weerasekara, Ana Marusic, Livia Puljak, Vinicius Tassoni Civile, Irena Zakarija-Grkovic, Tina Poklepovic Pericic, Alvaro Nagib Atallah, Santino Filoso, Nicola Luigi Bragazzi, Milena Soriano Marcolino, On behalf of the International Network of Coronavirus Disease 2019 (InterNetCOVID-19)

**Affiliations:** 1 University Hospital and School of Medicine, Universidade Federal de Minas Gerais, Belo Horizonte, Minas Gerais Brazil; 2 Medical College of Wisconsin, Milwaukee, WI USA; 3 Helene Fuld Health Trust National Institute for Evidence-based Practice in Nursing and Healthcare, College of Nursing, The Ohio State University, Columbus, OH USA; 4 School of Nursing, Psychotherapy and Community Health, Dublin City University, Dublin, Ireland; 5 Department of Anesthesiology, Intensive Care and Pain Medicine, University of Münster, Münster, Germany; 6 Department of Sport and Health Science, Technische Universität München, Munich, Germany; 7 School of Health Sciences, Faculty of Health and Medicine, The University of Newcastle, Callaghan, Australia; 8 Department of Physiotherapy, Faculty of Allied Health Sciences, University of Peradeniya, Peradeniya, Sri Lanka; 9 Cochrane Croatia, University of Split, School of Medicine, Split, Croatia; 10 Center for Evidence-Based Medicine and Health Care, Catholic University of Croatia, Ilica 242, 10000 Zagreb, Croatia; 11 Cochrane Brazil, Evidence-Based Health Program, Universidade Federal de São Paulo, São Paulo, Brazil; 12 Yorkville University, Fredericton, New Brunswick Canada; 13 Laboratory for Industrial and Applied Mathematics (LIAM), Department of Mathematics and Statistics, York University, Toronto, Ontario Canada

**Keywords:** Coronavirus, COVID-19, SARS-Cov-2, Evidence-based medicine, Infectious diseases

## Abstract

**Background:**

Navigating the rapidly growing body of scientific literature on the SARS-CoV-2 pandemic is challenging, and ongoing critical appraisal of this output is essential. We aimed to summarize and critically appraise systematic reviews of coronavirus disease (COVID-19) in humans that were available at the beginning of the pandemic.

**Methods:**

Nine databases (Medline, EMBASE, Cochrane Library, CINAHL, Web of Sciences, PDQ-Evidence, WHO’s Global Research, LILACS, and Epistemonikos) were searched from December 1, 2019, to March 24, 2020. Systematic reviews analyzing primary studies of COVID-19 were included. Two authors independently undertook screening, selection, extraction (data on clinical symptoms, prevalence, pharmacological and non-pharmacological interventions, diagnostic test assessment, laboratory, and radiological findings), and quality assessment (AMSTAR 2). A meta-analysis was performed of the prevalence of clinical outcomes.

**Results:**

Eighteen systematic reviews were included; one was empty (did not identify any relevant study). Using AMSTAR 2, confidence in the results of all 18 reviews was rated as “critically low”. Identified symptoms of COVID-19 were (range values of point estimates): fever (82–95%), cough with or without sputum (58–72%), dyspnea (26–59%), myalgia or muscle fatigue (29–51%), sore throat (10–13%), headache (8–12%) and gastrointestinal complaints (5–9%). Severe symptoms were more common in men. Elevated C-reactive protein and lactate dehydrogenase, and slightly elevated aspartate and alanine aminotransferase, were commonly described. Thrombocytopenia and elevated levels of procalcitonin and cardiac troponin I were associated with severe disease. A frequent finding on chest imaging was uni- or bilateral multilobar ground-glass opacity. A single review investigated the impact of medication (chloroquine) but found no verifiable clinical data. All-cause mortality ranged from 0.3 to 13.9%.

**Conclusions:**

In this overview of systematic reviews, we analyzed evidence from the first 18 systematic reviews that were published after the emergence of COVID-19. However, confidence in the results of all reviews was “critically low”. Thus, systematic reviews that were published early on in the pandemic were of questionable usefulness. Even during public health emergencies, studies and systematic reviews should adhere to established methodological standards.

**Supplementary Information:**

The online version contains supplementary material available at 10.1186/s12879-021-06214-4.

## Background

The spread of the “Severe Acute Respiratory Coronavirus 2” (SARS-CoV-2), the causal agent of COVID-19, was characterized as a pandemic by the World Health Organization (WHO) in March 2020 and has triggered an international public health emergency [1]. The numbers of confirmed cases and deaths due to COVID-19 are rapidly escalating, counting in millions [2], causing massive economic strain, and escalating healthcare and public health expenses [3, 4].

The research community has responded by publishing an impressive number of scientific reports related to COVID-19. The world was alerted to the new disease at the beginning of 2020 [1], and by mid-March 2020, more than 2000 articles had been published on COVID-19 in scholarly journals, with 25% of them containing original data [5]. The living map of COVID-19 evidence, curated by the Evidence for Policy and Practice Information and Co-ordinating Centre (EPPI-Centre), contained more than 40,000 records by February 2021 [6]. More than 100,000 records on PubMed were labeled as “SARS-CoV-2 literature, sequence, and clinical content” by February 2021 [7].

Due to publication speed, the research community has voiced concerns regarding the quality and reproducibility of evidence produced during the COVID-19 pandemic, warning of the potential damaging approach of “publish first, retract later” [8]. It appears that these concerns are not unfounded, as it has been reported that COVID-19 articles were overrepresented in the pool of retracted articles in 2020 [9]. These concerns about inadequate evidence are of major importance because they can lead to poor clinical practice and inappropriate policies [10].

Systematic reviews are a cornerstone of today’s evidence-informed decision-making. By synthesizing all relevant evidence regarding a particular topic, systematic reviews reflect the current scientific knowledge. Systematic reviews are considered to be at the highest level in the hierarchy of evidence and should be used to make informed decisions. However, with high numbers of systematic reviews of different scope and methodological quality being published, overviews of multiple systematic reviews that assess their methodological quality are essential [11–13]. An overview of systematic reviews helps identify and organize the literature and highlights areas of priority in decision-making.

In this overview of systematic reviews, we aimed to summarize and critically appraise systematic reviews of coronavirus disease (COVID-19) in humans that were available at the beginning of the pandemic.

## Methodology

### Research question

This overview’s primary objective was to summarize and critically appraise systematic reviews that assessed any type of primary clinical data from patients infected with SARS-CoV-2. Our research question was purposefully broad because we wanted to analyze as many systematic reviews as possible that were available early following the COVID-19 outbreak.

### Study design

We conducted an overview of systematic reviews. The idea for this overview originated in a protocol for a systematic review submitted to PROSPERO (CRD42020170623), which indicated a plan to conduct an overview.

Overviews of systematic reviews use explicit and systematic methods for searching and identifying multiple systematic reviews addressing related research questions in the same field to extract and analyze evidence across important outcomes. Overviews of systematic reviews are in principle similar to systematic reviews of interventions, but the unit of analysis is a systematic review [14–16].

We used the overview methodology instead of other evidence synthesis methods to allow us to collate and appraise multiple systematic reviews on this topic, and to extract and analyze their results across relevant topics [17]. The overview and meta-analysis of systematic reviews allowed us to investigate the methodological quality of included studies, summarize results, and identify specific areas of available or limited evidence, thereby strengthening the current understanding of this novel disease and guiding future research [13].

A reporting guideline for overviews of reviews is currently under development, i.e., Preferred Reporting Items for Overviews of Reviews (PRIOR) [18]. As the PRIOR checklist is still not published, this study was reported following the Preferred Reporting Items for Systematic Reviews and Meta-Analyses (PRISMA) 2009 statement [19]. The methodology used in this review was adapted from the *Cochrane Handbook for Systematic Reviews of Interventions* and also followed established methodological considerations for analyzing existing systematic reviews [14].

### Ethics

Approval of a research ethics committee was not necessary as the study analyzed only publicly available articles.

### Eligibility criteria

Systematic reviews were included if they analyzed primary data from patients infected with SARS-CoV-2 as confirmed by RT-PCR or another pre-specified diagnostic technique. Eligible reviews covered all topics related to COVID-19 including, but not limited to, those that reported clinical symptoms, diagnostic methods, therapeutic interventions, laboratory findings, or radiological results. Both full manuscripts and abbreviated versions, such as letters, were eligible.

No restrictions were imposed on the design of the primary studies included within the systematic reviews, the last search date, whether the review included meta-analyses or language. Reviews related to SARS-CoV-2 and other coronaviruses were eligible, but from those reviews, we analyzed only data related to SARS-CoV-2.

No consensus definition exists for a systematic review [20], and debates continue about the defining characteristics of a systematic review [21]. Cochrane’s guidance for overviews of reviews recommends setting pre-established criteria for making decisions around inclusion [14]. That is supported by a recent scoping review about guidance for overviews of systematic reviews [22].

Thus, for this study, we defined a systematic review as a research report which searched for primary research studies on a specific topic using an explicit search strategy, had a detailed description of the methods with explicit inclusion criteria provided, and provided a summary of the included studies either in narrative or quantitative format (such as a meta-analysis). Cochrane and non-Cochrane systematic reviews were considered eligible for inclusion, with or without meta-analysis, and regardless of the study design, language restriction and methodology of the included primary studies. To be eligible for inclusion, reviews had to be clearly analyzing data related to SARS-CoV-2 (associated or not with other viruses). We excluded narrative reviews without those characteristics as these are less likely to be replicable and are more prone to bias.

Scoping reviews and rapid reviews were eligible for inclusion in this overview if they met our pre-defined inclusion criteria noted above. We included reviews that addressed SARS-CoV-2 and other coronaviruses if they reported separate data regarding SARS-CoV-2.

### Information sources

Nine databases were searched for eligible records published between December 1, 2019, and March 24, 2020: Cochrane Database of Systematic Reviews via Cochrane Library, PubMed, EMBASE, CINAHL (Cumulative Index to Nursing and Allied Health Literature), Web of Sciences, LILACS (Latin American and Caribbean Health Sciences Literature), PDQ-Evidence, WHO’s Global Research on Coronavirus Disease (COVID-19), and Epistemonikos.

### Search

The comprehensive search strategy for each database is provided in Additional file 1 and was designed and conducted in collaboration with an information specialist. All retrieved records were primarily processed in EndNote, where duplicates were removed, and records were then imported into the Covidence platform [23]. In addition to database searches, we screened reference lists of reviews included after screening records retrieved via databases.

### Study selection

All searches, screening of titles and abstracts, and record selection, were performed independently by two investigators using the Covidence platform [23]. Articles deemed potentially eligible were retrieved for full-text screening carried out independently by two investigators. Discrepancies at all stages were resolved by consensus. During the screening, records published in languages other than English were translated by a native/fluent speaker.

### Data collection process

We custom designed a data extraction table for this study, which was piloted by two authors independently. Data extraction was performed independently by two authors. Conflicts were resolved by consensus or by consulting a third researcher.

### Data items

We extracted the following data: article identification data (authors’ name and journal of publication), search period, number of databases searched, population or settings considered, main results and outcomes observed, and number of participants. From Web of Science (Clarivate Analytics, Philadelphia, PA, USA), we extracted journal rank (quartile) and Journal Impact Factor (JIF).

We categorized the following as primary outcomes: all-cause mortality, need for and length of mechanical ventilation, length of hospitalization (in days), admission to intensive care unit (yes/no), and length of stay in the intensive care unit.

The following outcomes were categorized as exploratory: diagnostic methods used for detection of the virus, male to female ratio, clinical symptoms, pharmacological and non-pharmacological interventions, laboratory findings (full blood count, liver enzymes, C-reactive protein, d-dimer, albumin, lipid profile, serum electrolytes, blood vitamin levels, glucose levels, and any other important biomarkers), and radiological findings (using radiography, computed tomography, magnetic resonance imaging or ultrasound).

We also collected data on reporting guidelines and requirements for the publication of systematic reviews and meta-analyses from journal websites where included reviews were published.

### Quality assessment in individual reviews

Two researchers independently assessed the reviews’ quality using the “A MeaSurement Tool to Assess Systematic Reviews 2 (AMSTAR 2)”. We acknowledge that the AMSTAR 2 was created as “a critical appraisal tool for systematic reviews that include randomized or non-randomized studies of healthcare interventions, or both” [24]. However, since AMSTAR 2 was designed for systematic reviews of intervention trials, and we included additional types of systematic reviews, we adjusted some AMSTAR 2 ratings and reported these in Additional file 2.

Adherence to each item was rated as follows: yes, partial yes, no, or not applicable (such as when a meta-analysis was not conducted). The overall confidence in the results of the review is rated as “critically low”, “low”, “moderate” or “high”, according to the AMSTAR 2 guidance based on seven critical domains, which are items 2, 4, 7, 9, 11, 13, 15 as defined by AMSTAR 2 authors [24]. We reported our adherence ratings for transparency of our decision with accompanying explanations, for each item, in each included review.

One of the included systematic reviews was conducted by some members of this author team [25]. This review was initially assessed independently by two authors who were not co-authors of that review to prevent the risk of bias in assessing this study.

### Synthesis of results

For data synthesis, we prepared a table summarizing each systematic review. Graphs illustrating the mortality rate and clinical symptoms were created. We then prepared a narrative summary of the methods, findings, study strengths, and limitations.

For analysis of the prevalence of clinical outcomes, we extracted data on the number of events and the total number of patients to perform proportional meta-analysis using RStudio© software, with the “meta” package (version 4.9–6), using the “metaprop” function for reviews that did not perform a meta-analysis, excluding case studies because of the absence of variance. For reviews that did not perform a meta-analysis, we presented pooled results of proportions with their respective confidence intervals (95%) by the inverse variance method with a random-effects model, using the DerSimonian-Laird estimator for τ^2^. We adjusted data using Freeman-Tukey double arcosen transformation. Confidence intervals were calculated using the Clopper-Pearson method for individual studies. We created forest plots using the RStudio© software, with the “metafor” package (version 2.1–0) and “forest” function.

### Managing overlapping systematic reviews

Some of the included systematic reviews that address the same or similar research questions may include the same primary studies in overviews. Including such overlapping reviews may introduce bias when outcome data from the same primary study are included in the analyses of an overview multiple times. Thus, in summaries of evidence, multiple-counting of the same outcome data will give data from some primary studies too much influence [14]. In this overview, we did not exclude overlapping systematic reviews because, according to Cochrane’s guidance, it may be appropriate to include all relevant reviews’ results if the purpose of the overview is to present and describe the current body of evidence on a topic [14]. To avoid any bias in summary estimates associated with overlapping reviews, we generated forest plots showing data from individual systematic reviews, but the results were not pooled because some primary studies were included in multiple reviews.

## Results

**Fig. 1:**
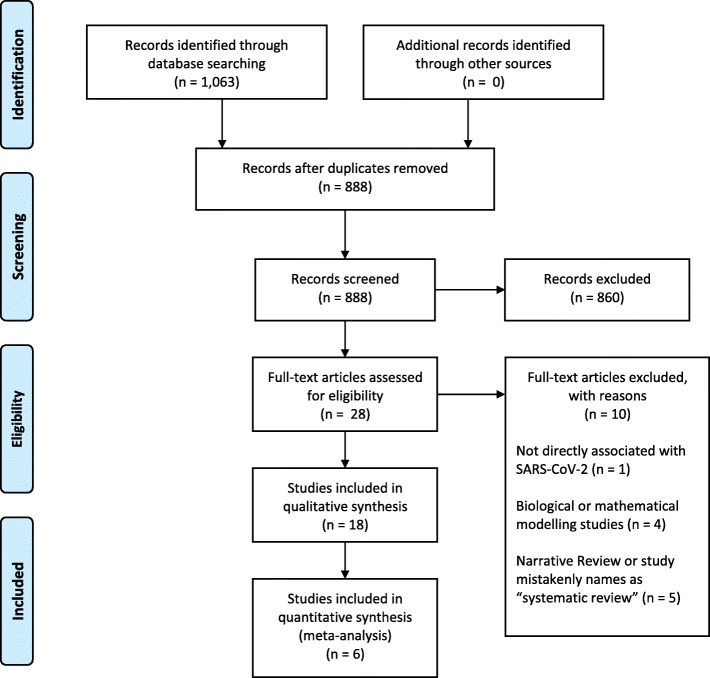
PRISMA flow diagram

Our search retrieved 1063 publications, of which 175 were duplicates. Most publications were excluded after the title and abstract analysis (*n* = 860). Among the 28 studies selected for full-text screening, 10 were excluded for the reasons described in Additional file 3, and 18 were included in the final analysis (Fig. 1) [25–42]. Reference list screening did not retrieve any additional systematic reviews.

### Characteristics of included reviews

Summary features of 18 systematic reviews are presented in Table 1. They were published in 14 different journals. Only four of these journals had specific requirements for systematic reviews (with or without meta-analysis): *European Journal of Internal Medicine, Journal of Clinical Medicine, Ultrasound in Obstetrics and Gynecology,* and *Clinical Research in Cardiology*. Two journals reported that they published only invited reviews (*Journal of Medical Virology* and *Clinica Chimica Acta*). Three systematic reviews in our study were published as letters; one was labeled as a scoping review and another as a rapid review (Table 2).

**Table 1:** Descriptive summary of the 18 systematic reviews included in the analysis (Adhikari is a scoping review; Mullins et al. is a rapid review)

Review identification	Journal and IF	Time frame assessed	Databases	Number of included studies	Study design of included studies	Methodological quality assessment	Meta-analysis performed	Patients: total (n)	Mean/Median age of included patients (years)	Male sex (%)
Adhikari et al	Infectious Diseases of Poverty, 3.12	January 1, 2020 to January 31, 2020	bioRxiv, medRxiv, chemRxiv, Google Scholar, PubMed, CNKI and WanFang Data; grey literature (online material released by the National Health Commission of the People’s Republic of China, Chinese Center for Disease Prevention and Control, and the WHO)	65	Cross-sectional, molecular, diagnostic, theoretical and mathematical modeling studies and reviews	N	N	Not reported	Median: 59, ranging from 2 to 89 years, when reported	Most cases were males; 59–68% where reported
Borges do Nascimento et al	Journal of Clinical Medicine, 5.688	January 1, 2019 to February 24, 2020	MEDLINE, Embase, CENTRAL (Cochrane Library), Scopus and LILACS	60	Case reports, case series and observational studies/epidemiological reports	Y	Y	59,254	Not reported; ranging from 3 months to 99 years	51.92
Cortegiani et al	Journal of Critical Care, 2.783	Inception up to March 1, 2020	PubMed and Embase, Chinese Clinical Trial Registry, ClinicalTrials.gov, and ICTPR (WHO)	29	Narrative letters, in-vitro study, editorial, expert consensus paper, national guideline documents and clinical trials^a^	N	N	3090	Not reported	Not reported
Li B et al. (Prevalence…)	Clinical Research in Cardiology, 4.907	December 2019 to February 2020	PubMed, medRxiv and Embase	6	Case series and observational studies	N	Y	1,527	Median: 49.75	57.80; ranging from 44.5 to 77
Li LQ et al. (2019 novel…)	Journal of Medical Virology, 2.049	December 2019 to February 2020	PubMed, Embase, Web of Science, WanFang Data, and CNKI	10.	Case reports, case series and observational studies	Y	Y	1994	Median: 49.94 (without one study)	63.35; ranging from 44.5 to 77
Lippi & Henry (Active smoking…)	European Journal of Internal Medicine, 3.660	2019 to March 9, 2020	MEDLINE (via PubMed interface), Scopus and Web of Science	5	Case series and observational studies	N	Y	1399	Not reported	More cases
Lippi et al. (Cardiac troponin…)	Progress in Cardiovascular Diseases, 6.162	2019 to March 4, 2020	MEDLINE (via PubMed interface), Scopus and Web of Science	4	Observational studies	N	Y	341	Not reported	Not rported
Lippi & Plebani (Procalcitonin…)	Clinica Chimica Acta, 2.735	Inception to March 3, 2020	MEDLINE (PubMed interface), Scopus and Web of Science	4	Observational studies	N	Y	Not reported	Not reported	Not reported
Lippi et al. (Thrombocyto…)	Clinica Chimica Acta, 2.735	2019 to March 6, 2020	Medline (PubMed interface), Scopus and Web of Science	9	Observational studies	N	Y	1,799	Not reported	71.71
Ludvigsson	Acta Pediatrica, 2.265	January 1, 2020 to March 18, 2020	MEDLINE (via PubMed interface), Embase and ICTPR	45	Case reports, case series and observational studies	N	N	Not summarized	6.7 (where reported) and newborn infants	56.6 (where reported)
Lupia et al	Journal of Global Antimicrobial Resistance, 2469	November 30, 2019 to February 13, 2020	PubMed and Cochrane Library	13	Case reports, case series and observational studies	N	N	874	42.25 (without one study ranging from 19 to 63)	53.67
Marasinghe	Systematic Review, 1.692	Inception up to February 2020	Cochrane Library, PubMed, Embase, Google Scholar and Scopus	0	Not applicable	Not applicable	Not applicable	Not applicable	Not applicable	Not applicable
Mullins et al	Ultrasound in Obstetrics & Gynecology, 5.595	Inception up to March 10, 2020	PubMed and MedRxiv	21	Case reports or case series	N	N	62 (32 mothers and 30 newborns)	Median: 30	Not applicable
Pang et al	Journal of Clinical Medicine, 5.688	December 1, 2019 to February 6, 2020	PubMed, Embase and Cochrane Library	27	Randomized controlled trials (RCTs) and validation trials	N	N	656 for SARS-COV-2	Not reported	Not reported
Rodriguez-Morales et al	Travel Medicine and Infectious Disease, 4.868	January 1, 2020 to February 23, 2020	MEDLINE (via PubMed interface), Scopus and Web of Science	58	Case reports, case series and cross-sectional studies	Y	Y (19 studies)	2874 (19 studies)	Mean: 51.97	55.9
Salehi et al	American Journal of Roentgenology, 3.161	Inception up to February 12, 2020 (updated on February 19, 2020)	PubMed, EMBASE, Google Scholar, and the World Health Organization Database	30	Case reports and case series	Y	N	919	Not reported	Not reported
Sun et al	Journal of Medical Virology, 2.049	Inception up to February 24, 2020	PubMed, Embase and Cochrane Library and additional databases/sources (including the China CDC)	10	Case reports, case series and observational studies	Y	Y	50,466	Mean: 44.25	52.01
Yang et al	International Journal of Infectious Diseases, 3.538	January 1, 2020 to February 25, 2020	PubMed, Embase and Web of Science	7	Case series and observational studies	N	Y	1576	Median: 46.0	51.6

^a^ Five studies did not fit the classification for study design: one narrative letter, one editorial, one expert consensus paper and two national guideline documents

**Table 2:** Main findings observed in the systematic reviews obtained

Review identification	General summary	Clinical symptoms	Diagnosis	Laboratory findings	Radiological findings	Therapeutic findings	Comorbidities evaluated	Quality of evidence	Overall quality assessment using AMSTAR 2	Review strengths and limitations
Adhikari et al	The study presents several categories of findings. Epidemiological findings showed that both the immunosuppressed and normal population appear susceptible to the COVID-19 infection. Biological analysis showed that SARS-CoV-2 is similar to coronaviruses found in bats. The effective reproductive number of SARS-CoV-2 (2.9) is higher than that of SARS (1.77). Virus transmission originated in Wuhan and from there it spread internationally. It is likely to be transmitted from human-to-human contact (via droplets or aerosol) as well as via surface contact. Prevention and control consist of isolation, identification and follow-up of contacts, environmental disinfection, and use of personal protective equipment.	The most commonly reported clinical symptoms are fever, cough, myalgia or fatigue, pneumonia, and complicated dyspnea, whereas less commonly reported symptoms include headache, diarrhea, hemoptysis, runny nose, and phlegm producing cough. Patients with mild symptoms were reported to recover after 1 week while severe cases were reported to experience progressive respiratory failure due to alveolar damage from the virus, which may lead to death.	Diagnosis of suspected cases used real-time fluorescence (RT-PCR) to detect the positive nucleic acid of SARS-CoV-2 in sputum, throat swabs, and secretions of the lower respiratory tract samples.	Decrease in lymphocytes and white blood cells.	New pulmonary infiltrates on chest radiography	No actual improvement in symptoms after 3 days of antibiotics treatment.	Cases resulting in death were primarily middle-aged and elderly patients with pre-existing diseases (tumor surgery, cirrhosis, hypertension, coronary heart disease, diabetes, and Parkinson’s disease).	Not available	Critically low	Early scoping report. Broad range of topics addressed. Narrative presentation of many results.
Borges do Nascimento et al	All-cause mortality was 0.3% (95% CI 0.0–1.0%). Epidemiological studies showed that mortality was higher in males and elderly patients	The incidence of symptoms were shown as following: Fever 82%, (CI) 56–99%; Cough 61, 95% CI 39–81%; Muscle aches and/or fatigue 36, 95% CI 18–55%; Dyspnea 26, 95% CI 12–41%; Headache 12, 95% CI 4–23%; Sore throat 10, 95% CI 5–17% and gastrointestinal symptoms 9,95% CI 3–17%.	Median time from onset of disease to diagnosis was 5 (interquartile ratio 2–9) days. In addition, Artificial intelligence has been recently raised as a potential tool to enhance care, and possibly be used for COVID-19 related cases.	Laboratory findings revealed lymphopenia (0.93 × 10^9^/L, 95% CI 0.83–1.03 × 10^9^/L and abnormal C-reactive protein (33.72 mg/dL, 95% CI 21.54–45.91 mg/dL.	Radiological findings varied, but mostly described ground-glass opacities and consolidation.	Antivirals (oseltamivir, umifenovir, ganciclovir, ritonavir) were reported as the most commonly used agents. Use of antibiotics was also reported (vancomycin, azithromycin, meropenem, cefaclor, cefepime and tazobactam). Other medications used were corticosteroids, alpha-interferon, immunoglobulin and antifungal drugs	The most prevalent co-morbidities were hypertension, diabetes, chronic liver disease and smoking.	All-cause mortality with a very low quality of evidence using GRADE	Critically low	Broad range of topics addressed
Cortegiani et al	Chloroquine seemed to be effective in limiting the replication of SARS-CoV-2 in vitro, justifying clinical research in patients with COVID-19. However, clinical use should adhere to the Monitored Emergency Use of Unregistered Interventions framework or be ethically approved as a trial	Not available	Not available	Not available	Not available	Not available	Not available	Not available	Critically low	Reviewed early pre-clinical evidence of effectiveness and safety of chloroquine, which justified following clinical research.
Li B et al	The median ages were, respectively, 56, 49, 47, 55.5, 34 and 57 years old according to the six studies. Patients with previous cardiovascular metabolic diseases are more likely to have a greater risk of developing into the severe condition and the comorbidities can also greatly affect the prognosis of the COVID-19. COVID-19 can also aggravate the damage to the heart.	Not available	The infection was diagnosed throughout the whole spectrum of age covering from new born to 92 years old.	Not available	Not available	Not available	The most prevalent comorbidities among confirmed cases of COVID-19 were hypertension (17.1%), cardiocerebrovascular disease (16.4%) and diabetes (9.7%). Patients with severe disease/in ICU were more likely to have hypertension, cardio-cerebrovascular diseases and diabetes than patients with non-severe disease/not in ICU; 8.0% of patients with COVID-19 suffered acute cardiac injury. Incidence of myocardial injury was ~ 13 times higher in patients with severe disease/in ICU than patients with non-severe disease/not in ICU.	Not available	Critically low	Assessed the prevalence important comorbidities.
Li LQ et al	The patients were 60% male (95% CI [0.54, 0.65]), the discharge rate was 42% (95% CI [0.29, 0.55]), and the fatality rate was 7% (95% CI [0.04,0.10]).	Clinical symptoms presented were: fever (88.5%), cough (68.6%), myalgia or fatigue (35.8%), expectoration (28.2%), and dyspnea (21.9%), headache or dizziness (12.1%), diarrhea (4.8%), nausea and vomiting (3.9%).	Not available	Laboratory results showed lymphocytopenia (64.5%), increase of C-reactive protein (44.3%), increase of lactic dehydrogenase (28.3%), and leukocytopenia (29.4%).	Not available	Not available	Not available	Not available	Critically low	Broad range of topics addressed
Lippi & Henry	Overall, in only one study active smoking was found to be a significant predictor of COVID-19 severity, whilst in the other four studies the association was not statistically significant.	Not available	Not available	No significant association was found between active smoking and severity of COVID-19 (OR, 1.69; 95% CI, 0.41–6.92; *p* = 0.254).	Not available	Not available	Smocking acticity	Not available	Critically low	Addressed the association of COVID-19 with an important comorbidity.
Lippi et al	cTnI values are significantly increased in patients with severe SARS-CoV-2 infection compared to those with milder forms of disease.	Not available	Not available	cTnI values were significantly increased in patients with severe COVID-19 compared to those with non-severe disease (SMD, 25.6 ng/L; 95% CI, 6.8–44.5 ng/L).	Not available	Not available	Not available	Not available	Critically low	Addressed biomarker with potential prognostic value.
Lippi & Plebani	Procalcitonin measurement may play a role for predicting evolution towards a more severe form of disease.	Not available	Not available	Results suggested that an increased procalcitonin value is associated with a higher risk of severe COVID-19 (OR, 4.76; 95% CI, 2.74–8.29).	Not available	Not available	Not available	Not available	Critically low	Addressed biomarker with potential prognostic value.
Lippi et al	Thrombocytopenia is associated with increased risk of severe disease and mortality in patients with COVID-19.	Not available	Not available	Platelet count was significantly lower in patients with more severe COVID-19 (weighted mean difference (WMD(−31 × 10^9^/L; 95% CI −35-29 × 10^9^/L). A subgroup analysis comparing patients by survival, found an even lower platelet count was observed with mortality (WMD, −48 × 10^9^/L; 95% CI −57 to -39 × 10^9^/L). A low platelet count was associated with over fivefold enhanced risk of severe COVID-19 (OR, 5.1; 95% CI 1.8–14.6).	Not available	Not available	Not available	Not available	Critically low	Addressed lab value with potential prognostic value.
Ludvigsson	Children account for 1–5% of diagnosed COVID-19 cases and they frequently have milder disease than adults; deaths have been extremely rare. Newborn infants have developed symptomatic COVID-19, but evidence of vertical intrauterine transmission is scarce.	Clinical characteristics presented mainly as fever and respiratory symptoms, and fewer children seem to have developed severe pneumonia.	Nasal and pharyngeal swabs or blood analysis are adequate samples for RT-PCR. Sequencing of specimens and clinical diagnosis have been used as alternative diagnostic approaches.	Elevated inflammatory markers were less common in children than adults and lymphocytopenia seemed rare.	Included studies described ground-glass opacities, local or bilateral patchy shadowing, and halo signs	Suggested treatment included providing oxygen, inhalations, nutritional support and maintaining fluids and electrolyte balances.	Not available	Not available	Critically low	Addressed symptoms and prognosis in children
Lupia et al	Most of the patients were male (age range, 8–92). Cardiovascular, digestive and endocrine system diseases were commonly reported.	Fever, cough, dyspnea, myalgia and fatigue were the most common symptoms.	Not available	Case studies reported leukopenia, thrombocytopenia, slightly elevated AST and ALT, and elevated C-reactive protein.	Multiple bilateral lobular and subsegmental areas of consolidation or bilateral GGOs were most commonly reported in chest CT findings.	Lopinavir, ritonavir, umifenovir and oseltamivir were the most common antivirals used to treat the infection. Supportive intervention (oxygen therapy) was frequently required by patients. Empirical antibiotics have been described. Steroids were also commonly described	Not available	Not available	Critically low	Summarizes findings from English-language case reports and case series.
Marasinghe	No studies were found investigating the effectiveness of face mask use in limiting the spread of this specific virus.	Not available	Not available	Not available	Not available	Not available	Not available	Not available	Critically low	Addressed an important preventive topic (face mask use).
Mullins et al	Study revealed that 7 mothers were asymptomatic (21.8%) and 2 mothers were admitted to the intensive care unit (6.25%). Delivery was by Caesarean section in 27 cases and by vaginal delivery in two, and 15 (47%) delivered preterm. There was one stillbirth and one neonatal death.	Seven patients were asymptomatic at admission while 18 were symptomatic (with viral changes on chest x-ray and chest tomography). Included symptoms were: cough, headache, chills, myalgia, sore throat, and shortness of breath.	Not available	Not available	Among included pregnant patients, evidence of pneumonia, bilateral infiltrates, ground-glass opacities, and consolidation were the most common radiological findings.	Not available	Included patients with asthma and pulmonary fibrosis.	Not available	Critically low	Addressed COVID-19 in pregnancy, delivery and postnatal.
Pang et al	The current diagnostic and therapeutic alternatives, including rapid diagnostics and vaccines are essential to limit transmission of respiratory infectious diseases such as the novel coronavirus.	Possible diagnostic approaches are RT-PCR, serological assays and point-of-care testing.	The study presented a detailed description of diagnostic methods, such as rapid tests, detection of genetical material and serological testing.	Not available	Not available	Several trials were identified, investigating therapeutics such as hydroxychloroquine, lopinavir and ritonavir, glucocorticoids therapy. Several vaccines are in development.	Not available	Not available	Critically low	Review focused on potential new diagnostics and therapeutics.
Rodriguez-Morales et al	20.3% (95% CI 10.0–30.6%) of patients required ICU support, 32.8% presented with acute respiratory distress syndrome (95% CI 13.7–51.8) and 6.2% (95% CI 3.1–9.3) with shock. The case fatality rate was 13.9% (95% CI 6.2–21.5%).	Clinical symptoms presented were fever (88.7, 95% CI 84.5–92.9%), cough (57.6, 95% CI 40.8–74.4%) and dyspnea (45.6, 95% CI 10.9–80.4%).	Not available	Regarding laboratory findings, decreased albumin (75.8, 95% CI 30.5–100.0%), high C-reactive protein (58.3, 95% CI 21.8–94.7%), and high lactate dehydrogenase (57.0, 95% CI 38.0–76.0), lymphopenia (43.1, 95% CI 18.9–67.3), and high erythrocyte sedimentation rate (41.8, 95% CI 0.0–92.8), were the most common laboratory results.	Results showed bilateral pneumonia, with associated ground-glass opacities.	Not available	Patients presented in 36.8% of cases with comorbidities (95% CI 24.7–48.9%), the most significant being hypertension (18.6, 95% CI 8.1–29.0%), cardiovascular disease (14.4, 95% CI 5.7–23.1%), and diabetes (11.9, 95% CI 9.1–14.6%), among others.	Not available	Critically low	Broad range of topics addressed.
Salehi et al	Known features of COVID-19 on initial CT include bilateral multilobar ground-glass opacification with a peripheral or posterior distribution, mainly in the lower lobes and less frequently within the right middle lobe. Septal thickening, bronchiectasis, pleural thickening, and subpleural involvement are some of the less common findings, mainly in the later stages of the disease.	Not available	Not available	Not available	A correlation was found between CT findings and disease severity and mortality. In severely ill patients, the most commonly reported CT findings were bilateral and multilobar involvement and subsegmental consolidative opacities. ARDS was the most common indication for transfer to the ICU, with the majority of COVID-19 mortalities occurring among patients with ARDS in the ICU.	Not available	Not available	Not available	Critically low	Focused review on radiological imaging.
Sun et al	The percentage of severe cases among all infected cases was 0.181 (95% CI: 0.127–0.243), and the case fatality rate was 0.043 (95% CI: 0.027, 0.061).	Clinical symptoms presented were fever 0.891 (95% CI: 0.818–0.945), cough 0.722 (95% CI: 0.657–0.782), muscle soreness or fatigue 0.425 (95% CI: 0.213–0.652). ARDS incidence was 0.148 (95% CI: 0.046–0.296).	Not available	Not available	The incidence of abnormal chest computer tomography was 0.966 (95% CI: 0.921–0.993).	Not available	Not available	Not available	Critically low	Broad range of topics addressed.
Yang et al	The symptoms of COVID-19 are similar to those of influenza (e. g, fever, cough or fatigue), and the COVID-19 outbreaks occurred during a year of a high prevalence of respiratory diseases caused by influenza, respiratory syncytial virus, and other respiratory viruses.	The most common clinical symptoms were fever (91 ± 3, 95% CI 86–97%), cough (67 ± 7, 95% CI 59–76%), fatigue (51 ± 0, 95% CI 34–68%) and dyspnea (30 ± 4, 95% CI 21–40%).	Not available	Not available	Not available	Not available	The most common comorbidities were hypertension (17 ± 7, 95% CI 14–22%), diabetes (8 ± 6, 95% CI 6–11%), cardiovascular diseases (5 ± 4, 95% CI 4–7%) and respiratory system diseases (2 ± 0, 95% CI 1–3%). There was a higher likelihood that patients with severe disease had hypertension (OR 2.36, 95% CI: 1.46–3.83), respiratory disease (OR 2.46, 95% CI: 1.76–3.44), or cardiovascular disease (OR 3.42, 95% CI: 1.88–6.22), compared with patients with non-severe disease.	Not available	Critically low	Review published in and early phase of the pandemic, which assessed symptoms and comorbidities.

List of abbreviations: *SARS-CoV-2* Severe Acute Respiratory Syndrome Coronavirus 2, *CI* Confidence Interval, *RT-PCR* Real Time Polymerase Chain Reaction, *GRADE* Grading of Recommendations, Assessment, Development and Evaluations, *ARDS* Acute Respiratory Distress Syndrome, *ICU* Intensive Care Unit, *AST* Aspartate Transaminase, *ALT* Alanine Transaminase, *cTnI* Cardiac Troponin I, *GGO* Ground-glass opacification

All reviews were published in English, in first quartile (Q1) journals, with JIF ranging from 1.692 to 6.062. One review was empty, meaning that its search did not identify any relevant studies; i.e., no primary studies were included [36]. The remaining 17 reviews included 269 unique studies; the majority (*N* = 211; 78%) were included in only a single review included in our study (range: 1 to 12). Primary studies included in the reviews were published between December 2019 and March 18, 2020, and comprised case reports, case series, cohorts, and other observational studies. We found only one review that included randomized clinical trials [38]. In the included reviews, systematic literature searches were performed from 2019 (entire year) up to March 9, 2020. Ten systematic reviews included meta-analyses. The list of primary studies found in the included systematic reviews is shown in Additional file 4, as well as the number of reviews in which each primary study was included.

### Population and study designs

Most of the reviews analyzed data from patients with COVID-19 who developed pneumonia, acute respiratory distress syndrome (ARDS), or any other correlated complication. One review aimed to evaluate the effectiveness of using surgical masks on preventing transmission of the virus [36], one review was focused on pediatric patients [34], and one review investigated COVID-19 in pregnant women [37]. Most reviews assessed clinical symptoms, laboratory findings, or radiological results.

### Systematic review findings

**Fig. 2:**
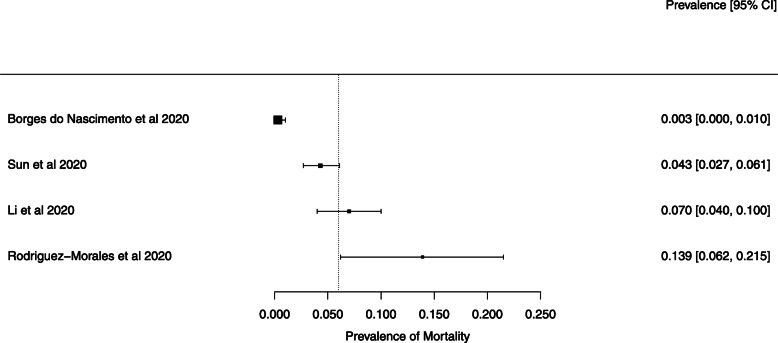
A meta-analysis of the prevalence of mortality

The summary of findings from individual reviews is shown in Table 2. Overall, all-cause mortality ranged from 0.3 to 13.9% (Fig. 2).

#### Clinical symptoms

**Fig. 3:**
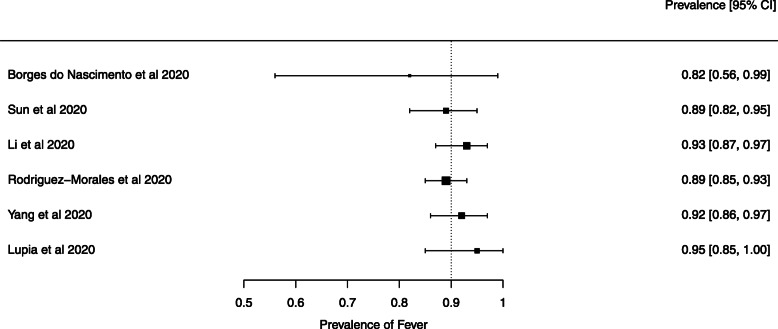
A meta-analysis of the prevalence of fever

**Fig. 4:**
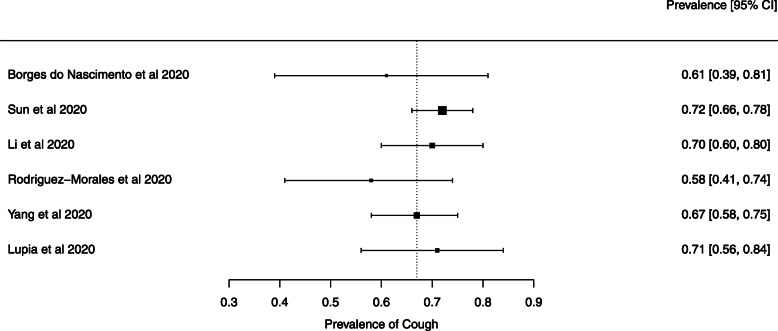
A meta-analysis of the prevalence of cough

**Fig. 5:**
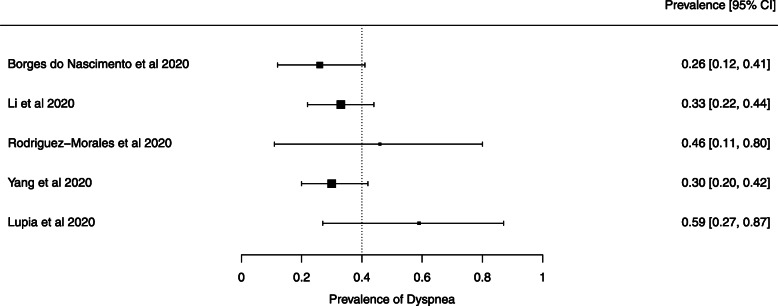
A meta-analysis of the prevalence of dyspnea

**Fig. 6:**
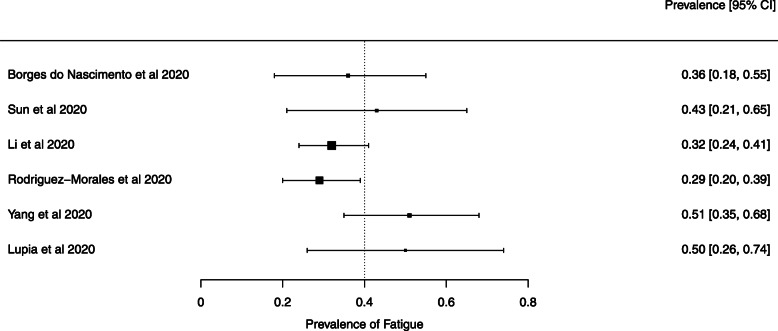
A meta-analysis of the prevalence of fatigue or myalgia

**Fig. 7:**
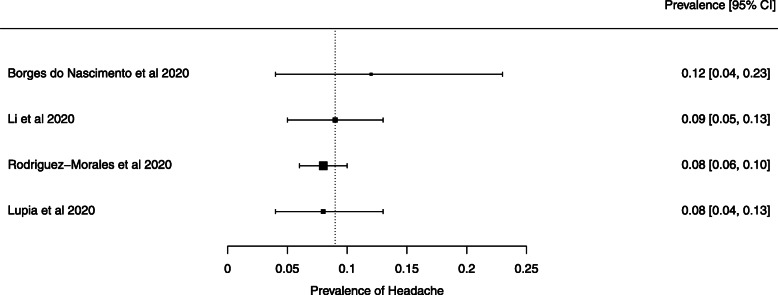
A meta-analysis of the prevalence of headache

**Fig. 8:**
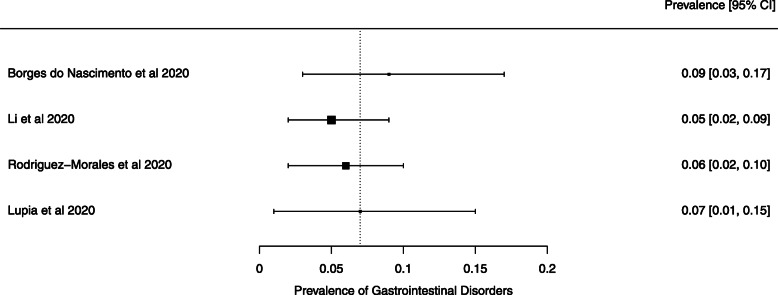
A meta-analysis of the prevalence of gastrointestinal disorders

**Fig. 9:**
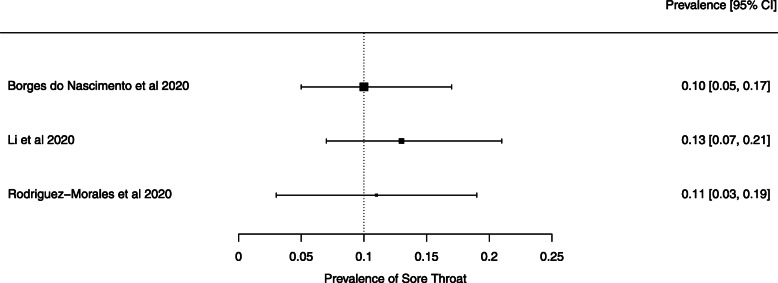
A meta-analysis of the prevalence of sore throat

Seven reviews described the main clinical manifestations of COVID-19 [26, 28, 29, 34, 35, 39, 41]. Three of them provided only a narrative discussion of symptoms [26, 34, 35]. In the reviews that performed a statistical analysis of the incidence of different clinical symptoms, symptoms in patients with COVID-19 were (range values of point estimates): fever (82–95%), cough with or without sputum (58–72%), dyspnea (26–59%), myalgia or muscle fatigue (29–51%), sore throat (10–13%), headache (8–12%), gastrointestinal disorders, such as diarrhea, nausea or vomiting (5.0–9.0%), and others (including, in one study only: dizziness 12.1%) (Figs. 3, 4, 5, 6, 7, 8 and 9). Three reviews assessed cough with and without sputum together; only one review assessed sputum production itself (28.5%).

#### Diagnostic aspects

Three reviews described methodologies, protocols, and tools used for establishing the diagnosis of COVID-19 [26, 34, 38]. The use of respiratory swabs (nasal or pharyngeal) or blood specimens to assess the presence of SARS-CoV-2 nucleic acid using RT-PCR assays was the most commonly used diagnostic method mentioned in the included studies. These diagnostic tests have been widely used, but their precise sensitivity and specificity remain unknown. One review included a Chinese study with clinical diagnosis with no confirmation of SARS-CoV-2 infection (patients were diagnosed with COVID-19 if they presented with at least two symptoms suggestive of COVID-19, together with laboratory and chest radiography abnormalities) [34].

#### Therapeutic possibilities

Pharmacological and non-pharmacological interventions (supportive therapies) used in treating patients with COVID-19 were reported in five reviews [25, 27, 34, 35, 38]. Antivirals used empirically for COVID-19 treatment were reported in seven reviews [25, 27, 34, 35, 37, 38, 41]; most commonly used were protease inhibitors (lopinavir, ritonavir, darunavir), nucleoside reverse transcriptase inhibitor (tenofovir), nucleotide analogs (remdesivir, galidesivir, ganciclovir), and neuraminidase inhibitors (oseltamivir). Umifenovir, a membrane fusion inhibitor, was investigated in two studies [25, 35]. Possible supportive interventions analyzed were different types of oxygen supplementation and breathing support (invasive or non-invasive ventilation) [25]. The use of antibiotics, both empirically and to treat secondary pneumonia, was reported in six studies [25–27, 34, 35, 38]. One review specifically assessed evidence on the efficacy and safety of the anti-malaria drug chloroquine [27]. It identified 23 ongoing trials investigating the potential of chloroquine as a therapeutic option for COVID-19, but no verifiable clinical outcomes data. The use of mesenchymal stem cells, antifungals, and glucocorticoids were described in four reviews [25, 34, 35, 38].

#### Laboratory and radiological findings

Of the 18 reviews included in this overview, eight analyzed laboratory parameters in patients with COVID-19 [25, 29, 30, 32–35, 39]; elevated C-reactive protein levels, associated with lymphocytopenia, elevated lactate dehydrogenase, as well as slightly elevated aspartate and alanine aminotransferase (AST, ALT) were commonly described in those eight reviews. Lippi et al. assessed cardiac troponin I (cTnI) [25], procalcitonin [32], and platelet count [33] in COVID-19 patients. Elevated levels of procalcitonin [32] and cTnI [30] were more likely to be associated with a severe disease course (requiring intensive care unit admission and intubation). Furthermore, thrombocytopenia was frequently observed in patients with complicated COVID-19 infections [33].

Chest imaging (chest radiography and/or computed tomography) features were assessed in six reviews, all of which described a frequent pattern of local or bilateral multilobar ground-glass opacity [25, 34, 35, 39–41]. Those six reviews showed that septal thickening, bronchiectasis, pleural and cardiac effusions, halo signs, and pneumothorax were observed in patients suffering from COVID-19.

### Quality of evidence in individual systematic reviews

Table 3 shows the detailed results of the quality assessment of 18 systematic reviews, including the assessment of individual items and summary assessment. A detailed explanation for each decision in each review is available in Additional file 5.

**Table 3:** Quality assessment rating of systematic reviews included in the COVID-19 overview

Study identification	AMSTAR 2 assessment for individual items																AMSTAR 2 Score Summary	Funding or support for the systematic review
	1	2	3	4	5	6	7	8	9	10	11	12	13	14	15	16	Final Rating	
Adhikari et al	Y	PY	Y	PY	Y	N	N	Y	N	N	NA	NA	N	Y	NA	Y	Critically Low	Y
Borges do Nascimento et al	Y	Y	Y	PY	Y	Y	N	Y	Y	N	Y	Y	Y	Y	N	Y	Critically Low	No
Cortegiani et al	Y	PY	Y	Y	Y	Y	N	Y	N	N	NA	NA	N	N	NA	Y	Critically Low	No
Li B et al. (Prevalence…)	Y	N	Y	N	N	N	N	PY	Y	N	Y	N	N	Y	Y	Y	Critically Low	Y
Li LQ et al. (2019 novel…)	Y	N	Y	PY	Y	Y	N	PY	Y	N	Y	Y	Y	Y	Y	Y	Critically Low	Y
Lippi & Henry (Active smoking…)	Y	N	N	PY	N	N	N	N	N	N	N	N	N	N	N	Y	Critically Low	Not reported
Lippi et al. (Cardiac troponin…)	Y	N	N	PY	N	N	N	N	N	N	N	N	N	N	N	Y	Critically Low	Not reported
Lippi & Plebani (Procalcitonin…)	Y	N	N	PY	N	N	N	N	N	N	N	N	N	Y	N	N	Critically Low	Not reported
Lippi et al. (Thrombocyto…)	Y	N	N	PY	Y	N	N	N	N	N	N	N	N	Y	N	Y	Critically Low	Not reported
Ludvigsson	Y	N	N	PY	N	N	N	N	N	N	NA	NA	N	N	NA	Y	Critically Low	No
Lupia et al	Y	N	N	N	N	N	N	N	N	N	NA	NA	N	N	NA	Y	Critically Low	No
Marasinghe	Y	N	Y	N	N	NA	N	NA	NA	NA	NA	NA	NA	NA	NA	Y	Critically Low	No
Mullins et al	Y	N	N	N	N	N	Y	N	N	N	NA	NA	N	N	NA	Y	Critically Low	No
Pang et al	N	N	Y	N	Y	N	N	Y	N	N	NA	NA	N	N	NA	Y	Critically Low	Y
Rodriguez-Morales et al	Y	Y	Y	PY	Y	Y	N	PY	N	N	Y	N	N	N	Y	Y	Critically Low	Y
Salehi et al	Y	N	N	N	Y	Y	N	N	Y	N	NA	NA	Y	N	NA	N	Critically Low	Not reported
Sun et al	Y	N	Y	PY	N	N	N	N	Y	N	Y	N	Y	Y	Y	Y	critically Low	Not reported
Yang et al	Y	N	Y	PY	N	Y	N	N	N	N	Y	N	N	Y	N	Y	Critically Low	Not reported

Note - Yes; *N* No; *PY* Partially yes; *NA* Not applicable

AMSTAR 2 Questions:

1. “Did the research questions and inclusion criteria for the review include the components of PICO?”

2. “Did the report of the review contain an explicit statement that the review methods were established prior to the conduct of the review and did the report justify any significant deviations from the protocol?”;

3. “Did the review authors explain their selection of the study designs for inclusion in the review?”;

4. “Did the review authors use a comprehensive literature search strategy?”

5. “Did the review authors perform study selection in duplicate?”;

6. “Did the review authors perform data extraction in duplicate?”;

7. “Did the review authors provide a list of excluded studies and justify the exclusions?”

8. “Did the review authors describe the included studies in adequate detail?”;

9. “Did the review authors use a satisfactory technique for assessing the risk of bias (RoB) in individual studies that were included in the review?”;

10. “Did the review authors report on the sources of funding for the studies included in the review?

11. “If meta-analysis was performed did the review authors use appropriate methods for statistical combination of results?”

12. “If meta-analysis was performed, did the review authors assess the potential impact of RoB in individual studies on the results of the meta-analysis or other evidence synthesis?”;

13. “Did the review authors account for RoB in individual studies when interpreting/ discussing the results of the review?”;

14. “Did the review authors provide a satisfactory explanation for, and discussion of, any heterogeneity observed in the results of the review?”;

15. “If they performed quantitative synthesis did the review authors carry out an adequate investigation of publication bias (small study bias) and discuss its likely impact on the results of the review?”;

16. “Did the review authors report any potential sources of conflict of interest, including any funding they received for conducting the review?”

*BOLD means critical domains

High confidence → No critical or maximum one non-critical weakness // Moderate confidence→ No critical with > 1 non-critical weaknesses

Low confidence→ One critical +/− non-critical weaknesses // Critically low confidence→ > 1 critical +/− non-critical weaknesses

Using AMSTAR 2 criteria, confidence in the results of all 18 reviews was rated as “critically low” (Table 3). Common methodological drawbacks were: omission of prospective protocol submission or publication; use of inappropriate search strategy: lack of independent and dual literature screening and data-extraction (or methodology unclear); absence of an explanation for heterogeneity among the studies included; lack of reasons for study exclusion (or rationale unclear).

Risk of bias assessment, based on a reported methodological tool, and quality of evidence appraisal, in line with the Grading of Recommendations Assessment, Development, and Evaluation (GRADE) method, were reported only in one review [25]. Five reviews presented a table summarizing bias, using various risk of bias tools [25, 29, 39–41]. One review analyzed “study quality” [37]. One review mentioned the risk of bias assessment in the methodology but did not provide any related analysis [28].

## Discussion

This overview of systematic reviews analyzed the first 18 systematic reviews published after the onset of the COVID-19 pandemic, up to March 24, 2020, with primary studies involving more than 60,000 patients. Using AMSTAR-2, we judged that our confidence in all those reviews was “critically low”. Ten reviews included meta-analyses. The reviews presented data on clinical manifestations, laboratory and radiological findings, and interventions. We found no systematic reviews on the utility of diagnostic tests.

Symptoms were reported in seven reviews; most of the patients had a fever, cough, dyspnea, myalgia or muscle fatigue, and gastrointestinal disorders such as diarrhea, nausea, or vomiting. Olfactory dysfunction (anosmia or dysosmia) has been described in patients infected with COVID-19 [43]; however, this was not reported in any of the reviews included in this overview. During the SARS outbreak in 2002, there were reports of impairment of the sense of smell associated with the disease [44, 45].

The reported mortality rates ranged from 0.3 to 14% in the included reviews. Mortality estimates are influenced by the transmissibility rate (basic reproduction number), availability of diagnostic tools, notification policies, asymptomatic presentations of the disease, resources for disease prevention and control, and treatment facilities; variability in the mortality rate fits the pattern of emerging infectious diseases [46]. Furthermore, the reported cases did not consider asymptomatic cases, mild cases where individuals have not sought medical treatment, and the fact that many countries had limited access to diagnostic tests or have implemented testing policies later than the others. Considering the lack of reviews assessing diagnostic testing (sensitivity, specificity, and predictive values of RT-PCT or immunoglobulin tests), and the preponderance of studies that assessed only symptomatic individuals, considerable imprecision around the calculated mortality rates existed in the early stage of the COVID-19 pandemic.

Few reviews included treatment data. Those reviews described studies considered to be at a very low level of evidence: usually small, retrospective studies with very heterogeneous populations. Seven reviews analyzed laboratory parameters; those reviews could have been useful for clinicians who attend patients suspected of COVID-19 in emergency services worldwide, such as assessing which patients need to be reassessed more frequently.

All systematic reviews scored poorly on the AMSTAR 2 critical appraisal tool for systematic reviews. Most of the original studies included in the reviews were case series and case reports, impacting the quality of evidence. Such evidence has major implications for clinical practice and the use of these reviews in evidence-based practice and policy. Clinicians, patients, and policymakers can only have the highest confidence in systematic review findings if high-quality systematic review methodologies are employed. The urgent need for information during a pandemic does not justify poor quality reporting.

We acknowledge that there are numerous challenges associated with analyzing COVID-19 data during a pandemic [47]. High-quality evidence syntheses are needed for decision-making, but each type of evidence syntheses is associated with its inherent challenges.

The creation of classic systematic reviews requires considerable time and effort; with massive research output, they quickly become outdated, and preparing updated versions also requires considerable time. A recent study showed that updates of non-Cochrane systematic reviews are published a median of 5 years after the publication of the previous version [48].

Authors may register a review and then abandon it [49], but the existence of a public record that is not updated may lead other authors to believe that the review is still ongoing. A quarter of Cochrane review protocols remains unpublished as completed systematic reviews 8 years after protocol publication [50].

Rapid reviews can be used to summarize the evidence, but they involve methodological sacrifices and simplifications to produce information promptly, with inconsistent methodological approaches [51]. However, rapid reviews are justified in times of public health emergencies, and even Cochrane has resorted to publishing rapid reviews in response to the COVID-19 crisis [52]. Rapid reviews were eligible for inclusion in this overview, but only one of the 18 reviews included in this study was labeled as a rapid review.

Ideally, COVID-19 evidence would be continually summarized in a series of high-quality living systematic reviews, types of evidence synthesis defined as “*a systematic review which is continually updated, incorporating relevant new evidence as it becomes available*” [53]. However, conducting living systematic reviews requires considerable resources, calling into question the sustainability of such evidence synthesis over long periods [54].

Research reports about COVID-19 will contribute to research waste if they are poorly designed, poorly reported, or simply not necessary. In principle, systematic reviews should help reduce research waste as they usually provide recommendations for further research that is needed or may advise that sufficient evidence exists on a particular topic [55]. However, systematic reviews can also contribute to growing research waste when they are not needed, or poorly conducted and reported. Our present study clearly shows that most of the systematic reviews that were published early on in the COVID-19 pandemic could be categorized as research waste, as our confidence in their results is critically low.

Our study has some limitations. One is that for AMSTAR 2 assessment we relied on information available in publications; we did not attempt to contact study authors for clarifications or additional data. In three reviews, the methodological quality appraisal was challenging because they were published as letters, or labeled as rapid communications. As a result, various details about their review process were not included, leading to AMSTAR 2 questions being answered as “not reported”, resulting in low confidence scores. Full manuscripts might have provided additional information that could have led to higher confidence in the results. In other words, low scores could reflect incomplete reporting, not necessarily low-quality review methods. To make their review available more rapidly and more concisely, the authors may have omitted methodological details. A general issue during a crisis is that speed and completeness must be balanced. However, maintaining high standards requires proper resourcing and commitment to ensure that the users of systematic reviews can have high confidence in the results.

Furthermore, we used adjusted AMSTAR 2 scoring, as the tool was designed for critical appraisal of reviews of interventions. Some reviews may have received lower scores than actually warranted in spite of these adjustments.

Another limitation of our study may be the inclusion of multiple overlapping reviews, as some included reviews included the same primary studies. According to the Cochrane Handbook, including overlapping reviews may be appropriate when the review’s aim is “*to present and describe the current body of systematic review evidence on a topic*” [12], which was our aim. To avoid bias with summarizing evidence from overlapping reviews, we presented the forest plots without summary estimates. The forest plots serve to inform readers about the effect sizes for outcomes that were reported in each review.

Several authors from this study have contributed to one of the reviews identified [25]. To reduce the risk of any bias, two authors who did not co-author the review in question initially assessed its quality and limitations.

Finally, we note that the systematic reviews included in our overview may have had issues that our analysis did not identify because we did not analyze their primary studies to verify the accuracy of the data and information they presented. We give two examples to substantiate this possibility. Lovato et al. wrote a commentary on the review of Sun et al. [41], in which they criticized the authors’ conclusion that sore throat is rare in COVID-19 patients [56]. Lovato et al. highlighted that multiple studies included in Sun et al. did not accurately describe participants’ clinical presentations, warning that only three studies clearly reported data on sore throat [56].

In another example, Leung [57] warned about the review of Li, L.Q. et al. [29]: “*it is possible that this statistic was computed using overlapped samples, therefore some patients were double counted*”. Li et al. responded to Leung that it is uncertain whether the data overlapped, as they used data from published articles and did not have access to the original data; they also reported that they requested original data and that they plan to re-do their analyses once they receive them; they also urged readers to treat the data with caution [58]. This points to the evolving nature of evidence during a crisis.

Our study’s strength is that this overview adds to the current knowledge by providing a comprehensive summary of all the evidence synthesis about COVID-19 available early after the onset of the pandemic. This overview followed strict methodological criteria, including a comprehensive and sensitive search strategy and a standard tool for methodological appraisal of systematic reviews.

## Conclusion

In conclusion, in this overview of systematic reviews, we analyzed evidence from the first 18 systematic reviews that were published after the emergence of COVID-19. However, confidence in the results of all the reviews was “critically low”. Thus, systematic reviews that were published early on in the pandemic could be categorized as research waste. Even during public health emergencies, studies and systematic reviews should adhere to established methodological standards to provide patients, clinicians, and decision-makers trustworthy evidence.

## Supplementary Information

**Additional file 1: Appendix 1.** Search strategies used in the study.

**Additional file 2: Appendix 2.** Adjusted scoring of AMSTAR 2 used in this study for systematic reviews of studies that did not analyze interventions.

**Additional file 3: Appendix 3.** List of excluded studies, with reasons.

**Additional file 4: Appendix 4.** Table of overlapping studies, containing the list of primary studies included, their visual overlap in individual systematic reviews, and the number in how many reviews each primary study was included.

**Additional file 5: Appendix 5.** A detailed explanation of AMSTAR scoring for each item in each review.

**Additional file 6: Appendix 6.** List of members and affiliates of International Network of Coronavirus Disease 2019 (InterNetCOVID-19).

## Data Availability

All data collected and analyzed within this study are available from the corresponding author on reasonable request.

## References

[1] World Health Organization. Timeline - COVID-19: Available at: https://www.who.int/news/item/29-06-2020-covidtimeline. Accessed 1 June 2021.

[2] COVID-19 Dashboard by the Center for Systems Science and Engineering (CSSE) at Johns Hopkins University (JHU). Available at: https://coronavirus.jhu.edu/map.html. Accessed 1 June 2021.

[3] Anzai A, Kobayashi T, Linton NM, Kinoshita R, Hayashi K, Suzuki A, et al. Assessing the Impact of Reduced Travel on Exportation Dynamics of Novel Coronavirus Infection (COVID-19). J Clin Med. 2020;9(2):601. 10.3390/jcm9020601 PMC7073579 32102279

[4] Chinazzi M, Davis JT, Ajelli M, Gioannini C, Litvinova M, Merler S, Pastore y Piontti A, Mu K, Rossi L, Sun K, Viboud C, Xiong X, Yu H, Halloran ME, Longini IM, Vespignani A (2020). The effect of travel restrictions on the spread of the 2019 novel coronavirus (COVID-19) outbreak. Science.

[5] Fidahic M, Nujic D, Runjic R, Civljak M, Markotic F, Lovric Makaric Z, Puljak L (2020). Research methodology and characteristics of journal articles with original data, preprint articles and registered clinical trial protocols about COVID-19. BMC Med Res Methodol.

[6] EPPI Centre*.* COVID-19: a living systematic map of the evidence. Available at: http://eppi.ioe.ac.uk/cms/Projects/DepartmentofHealthandSocialCare/Publishedreviews/COVID-19Livingsystematicmapoftheevidence/tabid/3765/Default.aspx. Accessed 1 June 2021.

[7] NCBI SARS-CoV-2 Resources. Available at: https://www.ncbi.nlm.nih.gov/sars-cov-2/. Accessed 1 June 2021.

[8] Gustot T (2020). Quality and reproducibility during the COVID-19 pandemic. JHEP Rep.

[9] Kodvanj, I., et al., Publishing of COVID-19 Preprints in Peer-reviewed Journals, Preprinting Trends, Public Discussion and Quality Issues. Preprint article. bioRxiv 2020.11.23.394577; doi: 10.1101/2020.11.23.394577*.* 10.1007/s11192-021-04249-7 PMC8801281 35125557

[10] Dobler CC (2020). Poor quality research and clinical practice during COVID-19. Breathe (Sheff).

[11] Bastian H, Glasziou P, Chalmers I (2010). Seventy-five trials and eleven systematic reviews a day: how will we ever keep up?. PLoS Med.

[12] Lunny C, Brennan SE, McDonald S, McKenzie JE (2017). Toward a comprehensive evidence map of overview of systematic review methods: paper 1-purpose, eligibility, search and data extraction. Syst Rev.

[13] Pollock M, Fernandes RM, Becker LA, Pieper D, Hartling L. Chapter V: Overviews of Reviews. In: Higgins JPT, Thomas J, Chandler J, Cumpston M, Li T, Page MJ, Welch VA (editors). Cochrane Handbook for Systematic Reviews of Interventions version 6.1 (updated September 2020). Cochrane. 2020. Available from www.training.cochrane.org/handbook.

[14] Higgins JPT, Thomas J, Chandler J, Cumpston M, Li T, Page MJ, et al. Cochrane handbook for systematic reviews of interventions version 6.1 (updated September 2020). Cochrane. 2020; Available from www.training.cochrane.org/handbook.

[15] Pollock M, Fernandes RM, Newton AS, Scott SD, Hartling L (2019). The impact of different inclusion decisions on the comprehensiveness and complexity of overviews of reviews of healthcare interventions. Syst Rev.

[16] Pollock M, Fernandes RM, Newton AS, Scott SD, Hartling L (2019). A decision tool to help researchers make decisions about including systematic reviews in overviews of reviews of healthcare interventions. Syst Rev.

[17] Hunt H, Pollock A, Campbell P, Estcourt L, Brunton G (2018). An introduction to overviews of reviews: planning a relevant research question and objective for an overview. Syst Rev.

[18] Pollock M, Fernandes RM, Pieper D, Tricco AC, Gates M, Gates A, Hartling L (2019). Preferred reporting items for overviews of reviews (PRIOR): a protocol for development of a reporting guideline for overviews of reviews of healthcare interventions. Syst Rev.

[19] Moher D, Liberati A, Tetzlaff J, Altman DG, PRISMA Group. Preferred reporting items for systematic reviews and meta-analyses: the PRISMA statement. Open Med. 2009;3(3):e123–30. PMC3090117 21603045

[20] Krnic Martinic M, Pieper D, Glatt A, Puljak L (2019). Definition of a systematic review used in overviews of systematic reviews, meta-epidemiological studies and textbooks. BMC Med Res Methodol.

[21] Puljak L (2017). If there is only one author or only one database was searched, a study should not be called a systematic review. J Clin Epidemiol.

[22] Gates M, Gates A, Guitard S, Pollock M, Hartling L (2020). Guidance for overviews of reviews continues to accumulate, but important challenges remain: a scoping review. Syst Rev.

[23] Covidence - systematic review software. Available at: https://www.covidence.org/. Accessed 1 June 2021.

[24] Shea BJ, Reeves BC, Wells G, Thuku M, Hamel C, Moran J, et al. AMSTAR 2: a critical appraisal tool for systematic reviews that include randomised or non-randomised studies of healthcare interventions, or both. BMJ. 2017;358:j4008. 10.1136/bmj.j4008 PMC5833365 28935701

[25] Borges do Nascimento IJ (2020). Novel Coronavirus Infection (COVID-19) in Humans: A Scoping Review and Meta-Analysis. J Clin Med.

[26] Adhikari SP, Meng S, Wu YJ, Mao YP, Ye RX, Wang QZ, Sun C, Sylvia S, Rozelle S, Raat H, Zhou H (2020). Epidemiology, causes, clinical manifestation and diagnosis, prevention and control of coronavirus disease (COVID-19) during the early outbreak period: a scoping review. Infect Dis Poverty.

[27] Cortegiani A, Ingoglia G, Ippolito M, Giarratano A, Einav S (2020). A systematic review on the efficacy and safety of chloroquine for the treatment of COVID-19. J Crit Care.

[28] Li B, Yang J, Zhao F, Zhi L, Wang X, Liu L, Bi Z, Zhao Y (2020). Prevalence and impact of cardiovascular metabolic diseases on COVID-19 in China. Clin Res Cardiol.

[29] Li LQ, Huang T, Wang YQ, Wang ZP, Liang Y, Huang TB, Zhang HY, Sun W, Wang Y (2020). COVID-19 patients’ clinical characteristics, discharge rate, and fatality rate of meta-analysis. J Med Virol.

[30] Lippi G, Lavie CJ, Sanchis-Gomar F (2020). Cardiac troponin I in patients with coronavirus disease 2019 (COVID-19): evidence from a meta-analysis. Prog Cardiovasc Dis.

[31] Lippi G, Henry BM (2020). Active smoking is not associated with severity of coronavirus disease 2019 (COVID-19). Eur J Intern Med.

[32] Lippi G, Plebani M (2020). Procalcitonin in patients with severe coronavirus disease 2019 (COVID-19): a meta-analysis. Clin Chim Acta.

[33] Lippi G, Plebani M, Henry BM (2020). Thrombocytopenia is associated with severe coronavirus disease 2019 (COVID-19) infections: a meta-analysis. Clin Chim Acta.

[34] Ludvigsson JF (2020). Systematic review of COVID-19 in children shows milder cases and a better prognosis than adults. Acta Paediatr.

[35] Lupia T, Scabini S, Mornese Pinna S, di Perri G, de Rosa FG, Corcione S (2020). 2019 novel coronavirus (2019-nCoV) outbreak: a new challenge. J Glob Antimicrob Resist.

[36] Marasinghe, K.M., A systematic review investigating the effectiveness of face mask use in limiting the spread of COVID-19 among medically not diagnosed individuals: shedding light on current recommendations provided to individuals not medically diagnosed with COVID-19. Research Square. Preprint article. doi*:*10.21203/rs.3.rs-16701/v1*.* 2020*.*

[37] Mullins E, Evans D, Viner RM, O’Brien P, Morris E (2020). Coronavirus in pregnancy and delivery: rapid review. Ultrasound Obstet Gynecol.

[38] Pang J, Wang MX, Ang IYH, Tan SHX, Lewis RF, Chen JIP, et al. Potential Rapid Diagnostics, Vaccine and Therapeutics for 2019 Novel coronavirus (2019-nCoV): a systematic review. J Clin Med. 2020;9(3):623. 10.3390/jcm9030623 PMC7141113 32110875

[39] Rodriguez-Morales AJ, Cardona-Ospina JA, Gutiérrez-Ocampo E, Villamizar-Peña R, Holguin-Rivera Y, Escalera-Antezana JP, Alvarado-Arnez LE, Bonilla-Aldana DK, Franco-Paredes C, Henao-Martinez AF, Paniz-Mondolfi A, Lagos-Grisales GJ, Ramírez-Vallejo E, Suárez JA, Zambrano LI, Villamil-Gómez WE, Balbin-Ramon GJ, Rabaan AA, Harapan H, Dhama K, Nishiura H, Kataoka H, Ahmad T, Sah R, Latin American Network of Coronavirus Disease 2019-COVID-19 Research (LANCOVID-19). Electronic address: https://www.lancovid.org (2020). Clinical, laboratory and imaging features of COVID-19: a systematic review and meta-analysis. Travel Med Infect Dis.

[40] Salehi S, Abedi A, Balakrishnan S, Gholamrezanezhad A (2020). Coronavirus disease 2019 (COVID-19): a systematic review of imaging findings in 919 patients. AJR Am J Roentgenol.

[41] Sun P, Qie S, Liu Z, Ren J, Li K, Xi J (2020). Clinical characteristics of hospitalized patients with SARS-CoV-2 infection: a single arm meta-analysis. J Med Virol.

[42] Yang J, Zheng Y, Gou X, Pu K, Chen Z, Guo Q, Ji R, Wang H, Wang Y, Zhou Y (2020). Prevalence of comorbidities and its effects in patients infected with SARS-CoV-2: a systematic review and meta-analysis. Int J Infect Dis.

[43] Bassetti M, Vena A, Giacobbe DR (2020). The novel Chinese coronavirus (2019-nCoV) infections: challenges for fighting the storm. Eur J Clin Investig.

[44] Hwang CS (2006). Olfactory neuropathy in severe acute respiratory syndrome: report of a case. Acta Neurol Taiwanica.

[45] Suzuki M, Saito K, Min WP, Vladau C, Toida K, Itoh H, Murakami S (2007). Identification of viruses in patients with postviral olfactory dysfunction. Laryngoscope.

[46] Rajgor DD, Lee MH, Archuleta S, Bagdasarian N, Quek SC (2020). The many estimates of the COVID-19 case fatality rate. Lancet Infect Dis.

[47] Wolkewitz M, Puljak L (2020). Methodological challenges of analysing COVID-19 data during the pandemic. BMC Med Res Methodol.

[48] Rombey T, Lochner V, Puljak L, Könsgen N, Mathes T, Pieper D (2020). Epidemiology and reporting characteristics of non-Cochrane updates of systematic reviews: a cross-sectional study. Res Synth Methods.

[49] Runjic E, Rombey T, Pieper D, Puljak L (2019). Half of systematic reviews about pain registered in PROSPERO were not published and the majority had inaccurate status. J Clin Epidemiol.

[50] Runjic E, Behmen D, Pieper D, Mathes T, Tricco AC, Moher D, Puljak L (2019). Following Cochrane review protocols to completion 10 years later: a retrospective cohort study and author survey. J Clin Epidemiol.

[51] Tricco AC, Antony J, Zarin W, Strifler L, Ghassemi M, Ivory J, Perrier L, Hutton B, Moher D, Straus SE (2015). A scoping review of rapid review methods. BMC Med.

[52] COVID-19 Rapid Reviews: Cochrane’s response so far. Available at: https://training.cochrane.org/resource/covid-19-rapid-reviews-cochrane-response-so-far. Accessed 1 June 2021.

[53] Cochrane. Living systematic reviews. Available at: https://community.cochrane.org/review-production/production-resources/living-systematic-reviews. Accessed 1 June 2021.

[54] Millard T, Synnot A, Elliott J, Green S, McDonald S, Turner T (2019). Feasibility and acceptability of living systematic reviews: results from a mixed-methods evaluation. Syst Rev.

[55] Babic A, Poklepovic Pericic T, Pieper D, Puljak L. How to decide whether a systematic review is stable and not in need of updating: analysis of Cochrane reviews. Res Synth Methods. 2020;11(6):884–90. 10.1002/jrsm.1451. 10.1002/jrsm.1451 32890455

[56] Lovato A, Rossettini G, de Filippis C (2020). Sore throat in COVID-19: comment on “clinical characteristics of hospitalized patients with SARS-CoV-2 infection: a single arm meta-analysis”. J Med Virol.

[57] Leung C (2020). Comment on Li et al: COVID-19 patients’ clinical characteristics, discharge rate, and fatality rate of meta-analysis. J Med Virol.

[58] Li LQ, Huang T, Wang YQ, Wang ZP, Liang Y, Huang TB, Zhang HY, Sun WM, Wang YP (2020). Response to Char’s comment: comment on Li et al: COVID-19 patients’ clinical characteristics, discharge rate, and fatality rate of meta-analysis. J Med Virol.

