# Supplementary material for: Coronavirus disease (COVID-19) pandemic: an overview of systematic reviews
Source: BMC Infect Dis. 2021 Jun 4;21:525. doi: 10.1186/s12879-021-06214-4 (PMC8177249; doi:10.1186/s12879-021-06214-4)
Supplement: Supplementary file 1 — Additional file 1: Appendix 1. Search strategies used in the study. [file 12879_2021_6214_MOESM1_ESM.docx]

**Appendix 1. Search strategies used in the study**

**PubMed**

(((((coronavirus[MeSH Terms]) OR coronavirus infections[MeSH Terms]) OR "betacoronavirus"[MeSH Terms]) OR "betacoronavirus 1"[MeSH Terms]) OR (Coronaviruses OR “Coronavirus Infection” OR "COVID-19" OR “Coronavirus Infection Disease 2019” OR “2019 Novel Coronavirus Infection” OR “2019-nCoV Infection” OR “2019 nCoV Infection” OR “2019-nCoV Infections” OR Betacoronavirus* OR “Novel Coronavirus Pneumonia” OR “2019 novel coronavirus" OR “coronavirus disease 2019” OR “nCoV” OR covid* OR “bat coronavirus”))

AND

((Meta-analysis OR “meta analysis” OR metaanalysis OR “systematic review”[Title/Abstract])

OR (systematic review OR PT meta-analysis[Publication Type]))

126 records

**Embase (Elsevier)**

'coronavirinae'/exp OR 'coronavirinae' OR 'betacoronavirus'/exp OR 'betacoronavirus' OR 'betacoronavirus 1'/exp OR 'betacoronavirus 1' OR coronaviruses OR 'coronavirus infection'/exp OR 'coronavirus infection' OR 'covid-19' OR 'coronavirus infection disease 2019' OR '2019 novel coronavirus infection' OR '2019-ncov infection' OR '2019 ncov infection' OR '2019-ncov infections' OR betacoronavirus* OR 'novel coronavirus pneumonia' OR '2019 novel coronavirus'/exp OR '2019 novel coronavirus' OR 'coronavirus disease 2019'/exp OR 'coronavirus disease 2019' OR 'ncov' OR covid* OR 'bat coronavirus'

AND

'meta analysis':ab,ti OR metaanalysis:ab,ti OR 'systematic review':ab,ti OR 'systematic review':it OR 'meta analysis':it

AND [embase]/lim NOT ([embase]/lim AND [medline]/lim)

91 records

**Latin American and Caribbean Center on Health Sciences Information (LILACS)**

MH:("Coronavirus Infection") OR ("Infecciones por Coronavirus") OR ("Infecções por Coronavirus") OR ("Coronavirus Infection") OR ("Infection, Coronavirus") OR ("Infections, Coronavirus") OR "Coronavirus" OR "Coronavirus" OR "Coronavirus" OR “bat coronavirus” OR MH:C02.782.600.550.200$ OR MH:B04.820.504.540.150$

AND (“systematic review” OR "estudo sistematico" OR "meta-analise" OR "meta-analisis" OR metaanalise OR metaanalisis)

432 records

**Cochrane CENTRAL**

(MeSH descriptor: [Coronavirus] explode all trees OR MeSH descriptor: [Betacoronavirus] explode all trees OR MeSH descriptor: [Coronavirus Infections] explode all trees OR Coronavirus* OR betacoronavirus* OR nCoV* OR novel coronavirus* OR novel corona virus OR covid* OR “bat coronavirus”)

258 records of systematic reviews

(160 records of protocols)

**CINAHL (Ebsco)**

TI ( ( "coronavirus" OR "coronavirus infections" OR "betacoronavirus" OR "betacoronavirus 1" OR "coronaviruses" OR "Coronavirus Infection" OR "COVID-19" OR "Coronavirus Infection Disease 2019" OR "2019 Novel Coronavirus Infection" OR "2019-nCoV Infection" OR "2019 nCoV Infection" OR "2019-nCoV Infections" OR "betacoronavirus*" OR "Novel Coronavirus Pneumonia" OR "2019 novel coronavirus" OR "coronavirus disease 2019" OR "nCoV" OR "covid" OR "coronavirinae" OR “bat coronavirus”) ) OR AB ( ( "coronavirus" OR "coronavirus infections" OR "betacoronavirus" OR "betacoronavirus 1" OR "coronaviruses" OR "Coronavirus Infection" OR "COVID-19" OR "Coronavirus Infection Disease 2019" OR "2019 Novel Coronavirus Infection" OR "2019-nCoV Infection" OR "2019 nCoV Infection" OR "2019-nCoV Infections" OR "betacoronavirus*" OR "Novel Coronavirus Pneumonia" OR "2019 novel coronavirus" OR "coronavirus disease 2019" OR "nCoV" OR "covid" OR "coronavirinae" OR “bat coronavirus”) )

AND
TI ( Meta-analysis OR “meta analysis” OR metaanalysis OR “systematic review” ) OR AB ( Meta-analysis OR “meta analysis” OR metaanalysis OR “systematic review” ) OR PT ( systematic review or meta-analysis )

22 records

**Web of Science**

TS =( "coronavirus" OR "coronavirus infections" OR "betacoronavirus" OR "betacoronavirus 1" OR "coronaviruses" OR "Coronavirus Infection" OR "COVID-19" OR "Coronavirus Infection Disease 2019" OR "2019 Novel Coronavirus Infection" OR "2019-nCoV Infection" OR "2019 nCoV Infection" OR "2019-nCoV Infections" OR "betacoronavirus*" OR "Novel Coronavirus Pneumonia" OR "2019 novel coronavirus" OR "coronavirus disease 2019" OR "nCoV" OR "covid" OR "coronavirinae" OR "bat coronavirus")

Databases= WOS, BIOSIS, CABI, FSTA, KJD, MEDLINE, RSCI, SCIELO, ZOOREC Timespan=All years

Search language=Auto

AND TS=("systematic review" OR meta-analysis OR "meta analysis" OR metaanalysis)

Databases= WOS, BIOSIS, CABI, FSTA, KJD, MEDLINE, RSCI, SCIELO, ZOOREC Timespan=All years

Search language=Auto

97 records

**Epistemonikos**

(((title:(( "coronavirus" OR "coronavirus infections" OR "betacoronavirus" OR "betacoronavirus 1" OR "coronaviruses" OR "Coronavirus Infection" OR "COVID-19" OR "Coronavirus Infection Disease 2019" OR "2019 Novel Coronavirus Infection" OR "2019-nCoV Infection" OR "2019 nCoV Infection" OR "2019-nCoV Infections" OR "betacoronavirus*" OR "Novel Coronavirus Pneumonia" OR "2019 novel coronavirus" OR "coronavirus disease 2019" OR "nCoV" OR "covid" OR "coronavirinae" OR "bat coronavirus")) OR abstract:(( "coronavirus" OR "coronavirus infections" OR "betacoronavirus" OR "betacoronavirus 1" OR "coronaviruses" OR "Coronavirus Infection" OR "COVID-19" OR "Coronavirus Infection Disease 2019" OR "2019 Novel Coronavirus Infection" OR "2019-nCoV Infection" OR "2019 nCoV Infection" OR "2019-nCoV Infections" OR "betacoronavirus*" OR "Novel Coronavirus Pneumonia" OR "2019 novel coronavirus" OR "coronavirus disease 2019" OR "nCoV" OR "covid" OR "coronavirinae" OR "bat coronavirus"))) AND (title:(Meta-analysis OR "meta analysis" OR metaanalysis OR "systematic review") OR abstract:(Meta-analysis OR "meta analysis" OR metaanalysis OR "systematic review"))) OR abstract:((title:(( "coronavirus" OR "coronavirus infections" OR "betacoronavirus" OR "betacoronavirus 1" OR "coronaviruses" OR "Coronavirus Infection" OR "COVID-19" OR "Coronavirus Infection Disease 2019" OR "2019 Novel Coronavirus Infection" OR "2019-nCoV Infection" OR "2019 nCoV Infection" OR "2019-nCoV Infections" OR "betacoronavirus*" OR "Novel Coronavirus Pneumonia" OR "2019 novel coronavirus" OR "coronavirus disease 2019" OR "nCoV" OR "covid" OR "coronavirinae" OR "bat coronavirus")) OR abstract:(( "coronavirus" OR "coronavirus infections" OR "betacoronavirus" OR "betacoronavirus 1" OR "coronaviruses" OR "Coronavirus Infection" OR "COVID-19" OR "Coronavirus Infection Disease 2019" OR "2019 Novel Coronavirus Infection" OR "2019-nCoV Infection" OR "2019 nCoV Infection" OR "2019-nCoV Infections" OR "betacoronavirus*" OR "Novel Coronavirus Pneumonia" OR "2019 novel coronavirus" OR "coronavirus disease 2019" OR "nCoV" OR "covid" OR "coronavirinae" OR "bat coronavirus"))) AND (title:(Meta-analysis OR "meta analysis" OR metaanalysis OR "systematic review") OR abstract:(Meta-analysis OR "meta analysis" OR metaanalysis OR "systematic review"))))

33 records

**PDQ Evidence**

"coronavirus" OR "coronavirus infections" OR "betacoronavirus" OR "betacoronavirus 1" OR "coronaviruses" OR "Coronavirus Infection" OR "COVID-19" OR "Coronavirus Infection Disease 2019" OR "2019 Novel Coronavirus Infection" OR "2019-nCoV Infection" OR "2019 nCoV Infection" OR "2019-nCoV Infections" OR "betacoronavirus*" OR "Novel Coronavirus Pneumonia" OR "2019 novel coronavirus" OR "coronavirus disease 2019" OR "nCoV" OR "covid" OR "coronavirinae" OR "bat coronavirus"

1 record, a primary study

World Health Organization (WHO) section for Systematic Reviews: 3 additional records

Total number of records: 1063 records, after deduplication in EndNote 888 records in total
