# Supplementary material for: Coronavirus disease (COVID-19) pandemic: an overview of systematic reviews
Source: BMC Infect Dis. 2021 Jun 4;21:525. doi: 10.1186/s12879-021-06214-4 (PMC8177249; doi:10.1186/s12879-021-06214-4)
Supplement: Supplementary file 2 — Additional file 2: Appendix 2. Adjusted scoring of AMSTAR 2 used in this study for systematic reviews of studies that did not analyze interventions. [file 12879_2021_6214_MOESM2_ESM.docx]

**Appendix 2. Adjusted scoring of AMSTAR 2 used in this study for systematic reviews of studies that did not analyze interventions**

**Item 1. Did the research questions and inclusion criteria for the review include the components of PICO?**

We considered that I-intervention and C-comparison were not eligible for systematic reviews (SRs) that are not SRs of interventions.

**Item 2. Did the report of the review contain an explicit statement that the review methods were established prior to the conduct of the review and did the report justify any significant deviations from the protocol?**

For scoping reviews, we considered this item of RoB/quality assessment “not applicable”, as authors of scoping revies do not typically assess RoB/quality of included studies.

**Item 3. Did the review authors explain their selection of the study designs for inclusion in the review?**

No adjustment

**Item 4. Did the review authors use a comprehensive literature search strategy?**

No adjustment

**Item 5. Did the review authors perform study selection in duplicate?**

No adjustment

**Item 6. Did the review authors perform data extraction in duplicate?**

No adjustment

**Item 7. Did the review authors provide a list of excluded studies and justify the exclusions?**

No adjustment

**Item 8. Did the review authors describe the included studies in adequate detail?**

We considered that I-intervention and C-comparison were not eligible for systematic reviews (SRs) that are not SRs of interventions. We scored “No” if there were no sufficient information on relevant items for the type of a review, for example, when there is no information on how cases were selected.

**Item 9. Did the review authors use a satisfactory technique for assessing the risk of bias (RoB) in individual studies that were included in the review?**

Since there are no widely adopted RoB (or quality) tools for critical appraisal of non-randomized studies that do not analyze interventions, we assessed whether the review used any tool to assess RoB/quality, regardless of which tool that was. For scoping reviews, we considered this item “not applicable”, as authors of scoping revies do not typically assess RoB/quality of included studies.

**Item 10. Did the review authors report on the sources of funding for the studies included in the review?**

No adjustment

**Item 11. If meta-analysis was performed did the review authors use appropriate methods for statistical combination of results?**

No adjustment

**Item 12. If meta-analysis was performed, did the review authors assess the potential impact of RoB in individual studies on the results of the meta-analysis or other evidence synthesis?**

No adjustment

**Item 13. Did the review authors account for RoB in individual studies when interpreting/ discussing the results of the review?**

The same consideration as for Item 9.

**Item 14. Did the review authors provide a satisfactory explanation for, and discussion of, any heterogeneity observed in the results of the review?**

No adjustment

**Item 15. If they performed quantitative synthesis did the review authors carry out an adequate investigation of publication bias (small study bias) and discuss its likely impact on the results of the review?**

No adjustment

**Item 16. Did the review authors report any potential sources of conflict of interest, including any funding they received for conducting the review?**

No adjustment
