# Supplementary material for: Coronavirus disease (COVID-19) pandemic: an overview of systematic reviews
Source: BMC Infect Dis. 2021 Jun 4;21:525. doi: 10.1186/s12879-021-06214-4 (PMC8177249; doi:10.1186/s12879-021-06214-4)
Supplement: Supplementary file 3 — Additional file 3: Appendix 3. List of excluded studies, with reasons. [file 12879_2021_6214_MOESM3_ESM.docx]

**Appendix 3. List of excluded studies, with reasons**

**Biological studies**

Xu, J.; Zhao, S.; Teng, T.; Abdalla, A.E.; Zhu, W.; Xie, L.; Wang, Y.; Guo, X. Systematic Comparison of Two Animal-to-Human Transmitted Human Coronaviruses: SARS-CoV-2 and SARS-CoV. *Viruses* 2020, *12*, 244.

Huang, J., Cao, Y., Bu, X. *et al.* Residue analysis of a CTL epitope of SARS-CoV spike protein by IFN-gamma production and bioinformatics prediction. *BMC Immunol* 13, 50 (2012). https://doi.org/10.1186/1471-2172-13-50

Liu Z, Xiao X, Wei X, Li J, Yang J, Tan H, Zhu J, Zhang Q, Wu J, Liu L. Composition and divergence of coronavirus spike proteins and host ACE2 receptors predict potential intermediate hosts of SARS-CoV-2. J Med Virol. 2020 Jun;92(6):595-601. doi: 10.1002/jmv.25726. Epub 2020 Mar 11. PMID: 32100877; PMCID: PMC7228221.

Wu A, Peng Y, Huang B, Ding X, Wang X, Niu P, Meng J, Zhu Z, Zhang Z, Wang J, Sheng J, Quan L, Xia Z, Tan W, Cheng G, Jiang T. Genome Composition and Divergence of the Novel Coronavirus (2019-nCoV) Originating in China. Cell Host Microbe. 2020 Mar 11;27(3):325-328. doi: 10.1016/j.chom.2020.02.001. Epub 2020 Feb 7. PMID: 32035028; PMCID: PMC7154514.

**Not a systematic review**

Bonilla-Aldana DK, Quintero-Rada K, Montoya-Posada JP, et al. SARS-CoV, MERS-CoV and now the 2019-novel CoV: Have we investigated enough about coronaviruses? - A bibliometric analysis. *Travel Med Infect Dis*. 2020;33:101566. doi:10.1016/j.tmaid.2020.101566

Shang L, Zhao J, Hu Y, Du R, Cao B. On the use of corticosteroids for 2019-nCoV pneumonia. Lancet. 2020 Feb 29;395(10225):683-684. doi: 10.1016/S0140-6736(20)30361-5. Epub 2020 Feb 12. PMID: 32122468; PMCID: PMC7159292.

Yao TT, Qian JD, Zhu WY, Wang Y, Wang GQ. A systematic review of lopinavir therapy for SARS coronavirus and MERS coronavirus-A possible reference for coronavirus disease-19 treatment option. J Med Virol. 2020 Jun;92(6):556-563. doi: 10.1002/jmv.25729. Epub 2020 Mar 12. PMID: 32104907; PMCID: PMC7217143.

Zhang L, Liu Y. Potential interventions for novel coronavirus in China: A systematic review. J Med Virol. 2020 May;92(5):479-490. doi: 10.1002/jmv.25707. Epub 2020 Mar 3. PMID: 32052466; PMCID: PMC7166986.

Tian HY. [2019-nCoV: new challenges from coronavirus]. Zhonghua Yu Fang Yi Xue Za Zhi. 2020 Mar 6;54(3):235-238. Chinese. doi: 10.3760/cma.j.issn.0253-9624.2020.03.002. PMID: 32187929.

**Wrong coronavirus**

Lin C, Ye R, Xia YL. A meta-analysis to evaluate the effectiveness of real-time PCR for diagnosing novel coronavirus infections. Genet Mol Res. 2015 Dec 2;14(4):15634-41. doi: 10.4238/2015.December.1.15. PMID: 26634531.

.
