# Supplementary material for: Coronavirus disease (COVID-19) pandemic: an overview of systematic reviews
Source: BMC Infect Dis. 2021 Jun 4;21:525. doi: 10.1186/s12879-021-06214-4 (PMC8177249; doi:10.1186/s12879-021-06214-4)
Supplement: Supplementary file 5 — Additional file 5: Appendix 5. A detailed explanation of AMSTAR scoring for each item in each review. [file 12879_2021_6214_MOESM5_ESM.docx]

**Appendix 5. A detailed explanation of AMSTAR scoring for each item in each review**

Contents

[Adhikari et al. 2](#_Toc64656291)

[Borges et al. 7](#_Toc64656292)

[Cortegiani et al. 12](#_Toc64656293)

[Li B. et al. 17](#_Toc64656294)

[Li LQ et al. 22](#_Toc64656295)

[Lippi et al. (Active smoking…) 27](#_Toc64656296)

[Lippi et al. (Cardiac troponin…) 32](#_Toc64656297)

[Lippi et al. (procalcitonin) 37](#_Toc64656298)

[Lippi et al. (Thrombocytopenia…) 42](#_Toc64656299)

[Ludvigsson et al. 47](#_Toc64656300)

[Lupia et al. 52](#_Toc64656301)

[Marasinghe 57](#_Toc64656302)

[Mullins et al. 62](#_Toc64656303)

[Pang et al. 67](#_Toc64656304)

[Rodriguez-Morales et al. 72](#_Toc64656305)

[Salehi et al. 77](#_Toc64656306)

[Sun et al. 82](#_Toc64656307)

[Yang et al. 87](#_Toc64656308)

# Adhikari et al.

| **1. Did the research questions and inclusion criteria for the review include the components of PICO?** | | | | | |
| --- | --- | --- | --- | --- | --- |
| For Yes:   🗷 Population  n/a Intervention  n/a Comparator group  🗷 Outcome | | Optional (recommended)   Timeframe for follow-up | 🗷   | Yes No |  |
|  Comments: Authors stated they used the Arskey & O’Malley method, which does not include O. However, this was taken to be the symptoms of COVID-19. As this was an SR of non-intervention studies, I-intervention and C-comparison were not applicable. | | | | |  |
| **2. Did the report of the review contain an explicit statement that the review methods were established prior to the conduct of the review and did the report justify any significant deviations from the protocol?** | | | | | |
|  | For Partial Yes:  The authors state that they had a written protocol or guide that included ALL the following:  🗷 review question(s)  🗷 a search strategy  🗷 inclusion/exclusion criteria  n/a a risk of bias assessment | For Yes:  As for partial yes, plus the protocol should be registered and should also have specified:   a meta-analysis/synthesis plan, if appropriate, *and*   a plan for investigating causes of heterogeneity   justification for any deviations from the protocol |   🗷   | Yes Partial Yes No |  |
|  | Comments: This was a scoping review, and in those authors do not typically assess the quality of included studies. | | | |  |
| **3. Did the review authors explain their selection of the study designs for inclusion in the review?** | | | | | |
|  | For Yes, the review should satisfy ONE of the following:   *Explanation for* including only RCTs   OR *Explanation for* including only NRSI   OR *Explanation for* including both RCTs and NRSI | | 🗷   | Yes No |  |
|  | Comments: An explicit explanation is not provided, but the authors included all types of study designs. | | | |  |
| **4. Did the review authors use a comprehensive literature search strategy?** | | | | | |
|  | For Partial Yes (all the following): | For Yes, should also have (all the following):   searched the reference lists / bibliographies of included studies   searched trial/study registries   included/consulted content experts in the field  🗷 where relevant, searched for grey literature  🗷 conducted search within 24 months of completion of the review |  |  |  |
|  | 🗷 searched at least 2 databases (relevant to research question)  🗷 provided key word and/or search strategy  🗷 justified publication restrictions |  |   🗷 | Yes Partial Yes No |  |
|  | (e.g. language) |  |  |  |  |
|  | Comments: Justification for language restriction implied in the Limitations (p. 10). | | | |  |
|  | **5. Did the review authors perform study selection in duplicate?** | |  | |  |
|  | For Yes, either ONE of the following:  🗷 at least two reviewers independently agreed on selection of eligible studies and achieved consensus on which studies to include   OR two reviewers selected a sample of eligible studies and achieved good agreement (at least 80 percent), with the remainder selected by one reviewer. | | 🗷 | Yes No |  |
|  | Comments: None | | | |  |

| **6. Did the review authors perform data extraction in duplicate?** | | | |
| --- | --- | --- | --- |
| For Yes, either ONE of the following:   at least two reviewers achieved consensus on which data to extract from included studies   OR two reviewers extracted data from a sample of eligible studies and achieved good agreement (at least 80 percent), with the remainder extracted by one reviewer. | | |  Yes  🗷 No |
| Comments: The authors did not report how many reviewers extracted data. | | | |
| **7. Did the review authors provide a list of excluded studies and justify the exclusions?** | | | |
|  | For Partial Yes:   provided a list of all potentially relevant studies that were read in full-text form but excluded from the review | For Yes, must also have:   Justified the exclusion from the review of each potentially relevant study |  Yes   Partial Yes  🗷 No |
|  | Comments: Only gave the 65 included studies, plus the number of excluded at full-text with reasons. | | |
| **8. Did the review authors describe the included studies in adequate detail?** | | | |
|  | For Partial Yes (ALL the following):  🗷 described populations  n/a described interventions  n/a described comparators  🗷 described outcomes  🗷 described research designs | For Yes, should also have ALL the following:  🗷 described population in detail  n/a described intervention in detail (including doses where relevant)  n/a described comparator in detail (including doses where relevant)  🗷 described study’s setting  🗷 timeframe for follow-up | 🗷 Yes   Partial Yes   No |
|  | Comments: The review provided a detailed summary of the various research designs. | | |
| **9. Did the review authors use a satisfactory technique for assessing the risk of bias (RoB) in individual studies that were included in the review?** | | | |
|  | **RCTs**  For Partial Yes, must have assessed RoB from   unconcealed allocation, *and*   lack of blinding of patients and assessors when assessing outcomes (unnecessary for objective outcomes such as all-cause mortality) | For Yes, must also have assessed RoB from:   allocation sequence that was not truly random, *and*   selection of the reported result from among multiple measurements or analyses of a specified outcome |  Yes   Partial Yes  🗷 No   Includes only NRSI |
|  | **NRSI**  For Partial Yes, must have assessed RoB:   from confounding, *and*   from selection bias | For Yes, must also have assessed RoB:   methods used to ascertain exposures and outcomes, *and*   selection of the reported result from among multiple measurements or analyses of a specified |  Yes   Partial Yes  🗷 No  Includes only RCTs |
| Comments: Not included in the Arskey & O’Malley method. This was a scoping review, and in those authors do not typically assess the quality of included studies. | | | |

| **10. Did the review authors report outcomes on the sources of funding for the studies included in the review?** | | | | | | |
| --- | --- | --- | --- | --- | --- | --- |
| For Yes:   Must have reported on the sources of funding for individual studies included in the review.  Note: Reporting that the reviewers looked for this information but it was not reported by study authors also qualifies. | | | |  Yes  🗷 No |  |  |
| Comments: None | | | |  |  |  |
| **11. If meta-analysis was performed did the review authors use appropriate methods for statistical combination of results?** | | | | | | |
|  | **RCTs**  For Yes:   The authors justified combining the data in a meta-analysis   AND they used an appropriate weighted technique to combine study results and adjusted for heterogeneity if present.   AND investigated the causes of any heterogeneity | |  Yes   No   No meta-analysis conducted | | |  |
|  | **For NRSI**  For Yes:   The authors justified combining the data in a meta-analysis   AND they used an appropriate weighted technique to combine study results, adjusting for heterogeneity if present   AND they statistically combined effect estimates from NRSI that were adjusted for confounding, rather than combining raw data, or justified combining raw data when adjusted effect estimates were not available   AND they reported separate summary estimates for RCTs and NRSI separately when both were included in the review | |  Yes   No  🗷 No meta-analysis conducted | | |  |
|  | Comments: None | | | | |  |
| **12. If meta-analysis was performed, did the review authors assess the potential impact of RoB in individual studies on the results of the meta-analysis or other evidence synthesis?** | | | | | | |
|  | For Yes:   included only low risk of bias RCTs   OR, if the pooled estimate was based on RCTs and/or NRSI at variable RoB, the authors performed analyses to investigate possible impact of RoB on summary estimates of effect. | |  Yes   No  🗷 No meta-analysis conducted | | |  |
|  | Comments: None | | | | |  |
| **13. Did the review authors account for RoB in individual studies when interpreting/ discussing the results of the review?** | | | | | | |
|  | For Yes:   included only low risk of bias RCTs   OR, if RCTs with moderate or high RoB, or NRSI were included the review provided a discussion of the likely impact of RoB on the results | |  Yes  🗷 No | | |  |
|  | Comments: Not included in the Arskey & O’Malley method. This was a scoping review, and in those authors do not typically assess the quality of included studies. | | | | |  |
| **14. Did the review authors provide a satisfactory explanation for, and discussion of, any heterogeneity observed in the results of the review?** | | | | | | |
|  | For Yes:   There was no significant heterogeneity in the results   OR if heterogeneity was present the authors performed an investigation of sources of any heterogeneity in the results and discussed the impact of this on the results of the review | | 🗷 Yes   No | | |  |
|  | Comments: Heterogeneity is not discussed explicitly, but the variety of types of studies is discussed throughout. | | | | |  |
| **15. If they performed quantitative synthesis did the review authors carry out an adequate investigation of publication bias (small study bias) and discuss its likely impact on the results of the review?** | | | | | | |
|  | For Yes:   performed graphical or statistical tests for publication bias and discussed the likelihood and magnitude of impact of publication bias | |  Yes   No  🗷 No meta-analysis conducted | | |  |
|  | Comments: None | | | | |  |
| **16. Did the review authors report any potential sources of conflict of interest, including any funding they received for conducting the review?** | | | | | | |
|  | For Yes:   The authors reported no competing interests OR  🗷 The authors described their funding sources and how they managed potential conflicts of interest | 🗷 Yes   No | | | |  |
|  | Comments: None | | | | |  |

**To cite this tool:** Shea BJ, Reeves BC, Wells G, Thuku M, Hamel C, Moran J, Moher D, Tugwell P, Welch V, Kristjansson E, Henry DA. AMSTAR 2: a critical appraisal tool for systematic reviews that include randomised or non-randomised studies of healthcare interventions, or both. BMJ. 2017 Sep 21;358:j4008.

# Borges et al.

| **1. Did the research questions and inclusion criteria for the review include the components of PICO?** | | | | | |
| --- | --- | --- | --- | --- | --- |
| For Yes:   🗷 Population  n/a Intervention  n/a Comparator group  🗷 Outcome | | Optional (recommended)   Timeframe for follow-up | 🗷 | Yes  No |  |
|  Comments: As this was a SR of non-intervention studies, I-intervention and C-comparison were not applicable. | | | | |  |
| **2. Did the report of the review contain an explicit statement that the review methods were established prior to the conduct of the review and did the report justify any significant deviations from the protocol?** | | | | | |
|  | For Partial Yes:  The authors state that they had a written protocol or guide that included ALL the following:   review question(s)   a search strategy   inclusion/exclusion criteria   a risk of bias assessment | For Yes:  As for partial yes, plus the protocol should be registered and should also have specified:   a meta-analysis/synthesis plan, if appropriate, *and*   a plan for investigating causes of heterogeneity   justification for any deviations from the protocol | 🗷   | Yes Partial Yes No |  |
|  | Comments: None | | | |  |
| **3. Did the review authors explain their selection of the study designs for inclusion in the review?** | | | | | |
|  | For Yes, the review should satisfy ONE of the following:   *Explanation for* including only RCTs   OR *Explanation for* including only NRSI   OR *Explanation for* including both RCTs and NRSI | | 🗷 | Yes No |  |
|  | Comments: All study designs included. | | | |  |
| **4. Did the review authors use a comprehensive literature search strategy?** | | | | | |
|  | For Partial Yes (all the following): | For Yes, should also have (all the following):  🗷 searched the reference lists / bibliographies of included studies   searched trial/study registries  🗷 included/consulted content experts in the field   where relevant, searched for grey literature  🗷 conducted search within 24 months of completion of the review |  |  |  |
|  | 🗷 searched at least 2 databases (relevant to research question)  🗷 provided key word and/or search strategy  🗷 justified publication restrictions |  |   🗷 | Yes Partial Yes No |  |
|  | (e.g. language) |  |  |  |  |
|  | Comments: All languages included. | | | |  |
|  | **5. Did the review authors perform study selection in duplicate?** | |  | |  |
|  | For Yes, either ONE of the following:   at least two reviewers independently agreed on selection of eligible studies and achieved consensus on which studies to include   OR two reviewers selected a sample of eligible studies and achieved good agreement (at least 80 percent), with the remainder selected by one reviewer. | | 🗷   | Yes No |  |
|  | Comments: None | | | |  |

| **6. Did the review authors perform data extraction in duplicate?** | | | |
| --- | --- | --- | --- |
| For Yes, either ONE of the following:   at least two reviewers achieved consensus on which data to extract from included studies   OR two reviewers extracted data from a sample of eligible studies and achieved good agreement (at least 80 percent), with the remainder extracted by one reviewer. | | | 🗷 Yes   No |
| Comments: None | | | |
| **7. Did the review authors provide a list of excluded studies and justify the exclusions?** | | | |
|  | For Partial Yes:   provided a list of all potentially relevant studies that were read in full-text form but excluded from the review | For Yes, must also have:   Justified the exclusion from the review of each potentially relevant study |  Yes   Partial Yes  🗷 No |
|  | Comments: Numbers of reasons for exclusion given, but not a list of excluded studies. | | |
| **8. Did the review authors describe the included studies in adequate detail?** | | | |
|  | For Partial Yes (ALL the following):  🗷 described populations  n/a described interventions  n/a described comparators  🗷 described outcomes  🗷 described research designs | For Yes, should also have ALL the following:  🗷 described population in detail  n/a described intervention in detail (including doses where relevant)  n/a described comparator in detail (including doses where relevant)  🗷 described study’s setting  🗷 timeframe for follow-up | 🗷 Yes   Partial Yes   No |
|  | Comments: None | | |
| **9. Did the review authors use a satisfactory technique for assessing the risk of bias (RoB) in individual studies that were included in the review?** | | | |
|  | **RCTs**  For Partial Yes, must have assessed RoB from   unconcealed allocation, *and*   lack of blinding of patients and assessors when assessing outcomes (unnecessary for objective outcomes such as all-cause mortality) | For Yes, must also have assessed RoB from:   allocation sequence that was not truly random, *and*   selection of the reported result from among multiple measurements or analyses of a specified outcome |  Yes   Partial Yes   No   Includes only NRSI |
|  | **NRSI**  For Partial Yes, must have assessed RoB:   from confounding, *and*   from selection bias | For Yes, must also have assessed RoB:   methods used to ascertain exposures and outcomes, *and*   selection of the reported result from among multiple measurements or analyses of a specified outcome |  🗷 Yes   Partial Yes   No  Includes only RCTs |
| Comments: Used Methodological Quality and Synthesis of Case Series and Case Reports Protocol. | | | |

| **10. Did the review authors report outcomes on the sources of funding for the studies included in the review?** | | | | | | |
| --- | --- | --- | --- | --- | --- | --- |
| For Yes:   Must have reported on the sources of funding for individual studies included in the review.  Note: Reporting that the reviewers looked for this information but it was not reported by study authors also qualifies. | | | |  Yes   🗷 No |  |  |
| Comments: None | | | |  |  |  |
| **11. If meta-analysis was performed did the review authors use appropriate methods for statistical combination of results?** | | | | | | |
|  | **RCTs**  For Yes:   The authors justified combining the data in a meta-analysis   AND they used an appropriate weighted technique to combine study results and adjusted for heterogeneity if present.   AND investigated the causes of any heterogeneity | |  Yes   No   No meta-analysis conducted | | |  |
|  | **For NRSI**  For Yes:  🗷 The authors justified combining the data in a meta-analysis  🗷 AND they used an appropriate weighted technique to combine study results, adjusting for heterogeneity if present  🗷 AND they statistically combined effect estimates from NRSI that were adjusted for confounding, rather than combining raw data, or justified combining raw data when adjusted effect estimates were not available   AND they reported separate summary estimates for RCTs and NRSI separately when both were included in the review | | 🗷 Yes   No   No meta-analysis conducted | | |  |
|  | Comments: None | | | | |  |
| **12. If meta-analysis was performed, did the review authors assess the potential impact of RoB in individual studies on the results of the meta-analysis or other evidence synthesis?** | | | | | | |
|  | For Yes:   included only low risk of bias RCTs   OR, if the pooled estimate was based on RCTs and/or NRSI at variable RoB, the authors performed analyses to investigate possible impact of RoB on summary estimates of effect. | | 🗷 Yes   No   No meta-analysis conducted | | |  |
|  | Comments: None | | | | |  |
| **13. Did the review authors account for RoB in individual studies when interpreting/ discussing the results of the review?** | | | | | | |
|  | For Yes:   included only low risk of bias RCTs  🗷 OR, if RCTs with moderate or high RoB, or NRSI were included the review provided a discussion of the likely impact of RoB on the results | | 🗷 Yes   No | | |  |
|  | Comments: None | | | | |  |
| **14. Did the review authors provide a satisfactory explanation for, and discussion of, any heterogeneity observed in the results of the review?** | | | | | | |
|  | For Yes:   There was no significant heterogeneity in the results  🗷 OR if heterogeneity was present the authors performed an investigation of sources of any heterogeneity in the results and discussed the impact of this on the results of the review | | 🗷 Yes   No | | |  |
|  | Comments: None | | | | |  |
| **15. If they performed quantitative synthesis did the review authors carry out an adequate investigation of publication bias (small study bias) and discuss its likely impact on the results of the review?** | | | | | | |
|  | For Yes:   performed graphical or statistical tests for publication bias and discussed the likelihood and magnitude of impact of publication bias | |  Yes  🗷 No   No meta-analysis conducted | | |  |
|  | Comments: None | | | | |  |
| **16. Did the review authors report any potential sources of conflict of interest, including any funding they received for conducting the review?** | | | | | | |
|  | For Yes:  🗷 The authors reported no competing interests OR   The authors described their funding sources and how they managed potential conflicts of interest | 🗷 Yes   No | | | |  |
|  | Comments: None | | | | |  |

**To cite this tool:** Shea BJ, Reeves BC, Wells G, Thuku M, Hamel C, Moran J, Moher D, Tugwell P, Welch V, Kristjansson E, Henry DA. AMSTAR 2: a critical appraisal tool for systematic reviews that include randomised or non-randomised studies of healthcare interventions, or both. BMJ. 2017 Sep 21;358:j4008.

# Cortegiani et al.

| **1. Did the research questions and inclusion criteria for the review include the components of PICO?** | | | | | |
| --- | --- | --- | --- | --- | --- |
| For Yes:   🗷 Population  🗷 Intervention  🗷 Comparator group  🗷 Outcome | | Optional (recommended)   Timeframe for follow-up | 🗷 | Yes No |  |
|  Comments: I = chloroquine | | | | |  |
| **2. Did the report of the review contain an explicit statement that the review methods were established prior to the conduct of the review and did the report justify any significant deviations from the protocol?** | | | | | |
|  | For Partial Yes:  The authors state that they had a written protocol or guide that included ALL the following:   review question(s)   a search strategy   inclusion/exclusion criteria   a risk of bias assessment | For Yes:  As for partial yes, plus the protocol should be registered and should also have specified:   a meta-analysis/synthesis plan, if appropriate, *and*   a plan for investigating causes of heterogeneity   justification for any deviations from the protocol |   🗷   | Yes Partial Yes No |  |
|  | Comments: They stated they had a protocol but did not register it due to the urgency of getting data released. However, they did not explicitly state what was addressed in the protocol. | | | |  |
| **3. Did the review authors explain their selection of the study designs for inclusion in the review?** | | | | | |
|  | For Yes, the review should satisfy ONE of the following:   *Explanation for* including only RCTs   OR *Explanation for* including only NRSI   OR *Explanation for* including both RCTs and NRSI | | 🗷   | Yes No |  |
|  | Comments: No restrictions were placed on study designs in the search strategy. | | | |  |
| **4. Did the review authors use a comprehensive literature search strategy?** | | | | | |
|  | For Partial Yes (all the following): | For Yes, should also have (all the following):  🗷 searched the reference lists / bibliographies of included studies  🗷searched trial/study registries  🗷 included/consulted content experts in the field  🗷 where relevant, searched for grey literature  🗷 conducted search within 24 months of completion of the review |  |  |  |
|  | 🗷 searched at least 2 databases (relevant to research question)  🗷 provided key word and/or search strategy  🗷 justified publication restrictions |  | 🗷   | Yes Partial Yes No |  |
|  | (e.g. language) |  |  |  |  |
|  | Comments: Authors would seem to be content experts, which is the only criterion not explicitly addressed. | | | |  |
|  | **5. Did the review authors perform study selection in duplicate?** | |  | |  |
|  | For Yes, either ONE of the following:  🗷 at least two reviewers independently agreed on selection of eligible studies and achieved consensus on which studies to include   OR two reviewers selected a sample of eligible studies and achieved good agreement (at least 80 percent), with the remainder selected by one reviewer. | | 🗷 | Yes No |  |
|  | Comments: None | | | |  |

| **6. Did the review authors perform data extraction in duplicate?** | | | |
| --- | --- | --- | --- |
| For Yes, either ONE of the following:  🗷 at least two reviewers achieved consensus on which data to extract from included studies   OR two reviewers extracted data from a sample of eligible studies and achieved good agreement (at least 80 percent), with the remainder extracted by one reviewer. | | | 🗷 Yes   No |
| Comments: None | | | |
| **7. Did the review authors provide a list of excluded studies and justify the exclusions?** | | | |
|  | For Partial Yes:   provided a list of all potentially relevant studies that were read in full-text form but excluded from the review | For Yes, must also have:   Justified the exclusion from the review of each potentially relevant study |  Yes   Partial Yes  🗷 No |
|  | Comments: 8 articles were read in full text, but 6 included. Other 2 not identified. | | |
| **8. Did the review authors describe the included studies in adequate detail?** | | | |
|  | For Partial Yes (ALL the following):  🗷 described populations  🗷 described interventions  🗷 described comparators  🗷 described outcomes  🗷 described research designs | For Yes, should also have ALL the following:  🗷 described population in detail  🗷 described intervention in detail (including doses where relevant)  🗷 described comparator in detail (including doses where relevant)  🗷 described study’s setting  🗷 timeframe for follow-up | 🗷 Yes   Partial Yes   No |
|  | Comments: None | | |
| **9. Did the review authors use a satisfactory technique for assessing the risk of bias (RoB) in individual studies that were included in the review?** | | | |
|  | **RCTs**  For Partial Yes, must have assessed RoB from   unconcealed allocation, *and*   lack of blinding of patients and assessors when assessing outcomes (unnecessary for objective outcomes such as all-cause mortality) | For Yes, must also have assessed RoB from:   allocation sequence that was not truly random, *and*   selection of the reported result from among multiple measurements or analyses of a specified outcome |  Yes   Partial Yes  🗷 No   Includes only NRSI |
|  | **NRSI**  For Partial Yes, must have assessed RoB:   from confounding, *and*   from selection bias | For Yes, must also have assessed RoB:   methods used to ascertain exposures and outcomes, *and*   selection of the reported result from among multiple measurements or analyses of a specified |  Yes   Partial Yes  🗷 No  Includes only RCTs |
| Comments: None | | | |

| **10. Did the review authors report outcomes on the sources of funding for the studies included in the review?** | | | | | | |
| --- | --- | --- | --- | --- | --- | --- |
| For Yes:   Must have reported on the sources of funding for individual studies included in the review.  Note: Reporting that the reviewers looked for this information but it was not reported by study authors also qualifies. | | | |  Yes  🗷 No |  |  |
| Comments: None | | | |  |  |  |
| **11. If meta-analysis was performed did the review authors use appropriate methods for statistical combination of results?** | | | | | | |
|  | **RCTs**  For Yes:   The authors justified combining the data in a meta-analysis   AND they used an appropriate weighted technique to combine study results and adjusted for heterogeneity if present.   AND investigated the causes of any heterogeneity | |  Yes   No  🗷 No meta-analysis conducted | | |  |
|  | **For NRSI**  For Yes:   The authors justified combining the data in a meta-analysis   AND they used an appropriate weighted technique to combine study results, adjusting for heterogeneity if present   AND they statistically combined effect estimates from NRSI that were adjusted for confounding, rather than combining raw data, or justified combining raw data when adjusted effect estimates were not available   AND they reported separate summary estimates for RCTs and NRSI separately when both were included in the review | |  Yes   No  🗷 No meta-analysis conducted | | |  |
|  | Comments: None | | | | |  |
| **12. If meta-analysis was performed, did the review authors assess the potential impact of RoB in individual studies on the results of the meta-analysis or other evidence synthesis?** | | | | | | |
|  | For Yes:   included only low risk of bias RCTs   OR, if the pooled estimate was based on RCTs and/or NRSI at variable RoB, the authors performed analyses to investigate possible impact of RoB on summary estimates of effect. | |  Yes   No  🗷 No meta-analysis conducted | | |  |
|  | Comments: None | | | | |  |
| **13. Did the review authors account for RoB in individual studies when interpreting/ discussing the results of the review?** | | | | | | |
|  | For Yes:   included only low risk of bias RCTs   OR, if RCTs with moderate or high RoB, or NRSI were included the review provided a discussion of the likely impact of RoB on the results | |  Yes  🗷 No | | |  |
|  | Comments: None | | | | |  |
| **14. Did the review authors provide a satisfactory explanation for, and discussion of, any heterogeneity observed in the results of the review?** | | | | | | |
|  | For Yes:   There was no significant heterogeneity in the results   OR if heterogeneity was present the authors performed an investigation of sources of any heterogeneity in the results and discussed the impact of this on the results of the review | |  Yes  🗷 No | | |  |
|  | Comments: None | | | | |  |
| **15. If they performed quantitative synthesis did the review authors carry out an adequate investigation of publication bias (small study bias) and discuss its likely impact on the results of the review?** | | | | | | |
|  | For Yes:   performed graphical or statistical tests for publication bias and discussed the likelihood and magnitude of impact of publication bias | |  Yes   No  🗷 No meta-analysis conducted | | |  |
|  | Comments: None | | | | |  |
| **16. Did the review authors report any potential sources of conflict of interest, including any funding they received for conducting the review?** | | | | | | |
|  | For Yes:  🗷 The authors reported no competing interests OR   The authors described their funding sources and how they managed potential conflicts of interest | 🗷 Yes   No | | | |  |
|  | Comments: None | | | | |  |

**To cite this tool:** Shea BJ, Reeves BC, Wells G, Thuku M, Hamel C, Moran J, Moher D, Tugwell P, Welch V, Kristjansson E, Henry DA. AMSTAR 2: a critical appraisal tool for systematic reviews that include randomised or non-randomised studies of healthcare interventions, or both. BMJ. 2017 Sep 21;358:j4008.

# Li B. et al.

| **1. Did the research questions and inclusion criteria for the review include the components of PICO?** | | | | | |
| --- | --- | --- | --- | --- | --- |
| For Yes:   🗷 Population  n/a Intervention  n/a Comparator group  🗷 Outcome | | Optional (recommended)   Timeframe for follow-up | 🗷 | Yes  No |  |
|  Comments: As this was a SR of non-intervention studies, I-intervention and C-comparison were not applicable. | | | | |  |
| **2. Did the report of the review contain an explicit statement that the review methods were established prior to the conduct of the review and did the report justify any significant deviations from the protocol?** | | | | | |
|  | For Partial Yes:  The authors state that they had a written protocol or guide that included ALL the following:   review question(s)   a search strategy   inclusion/exclusion criteria   a risk of bias assessment | For Yes:  As for partial yes, plus the protocol should be registered and should also have specified:   a meta-analysis/synthesis plan, if appropriate, *and*   a plan for investigating causes of heterogeneity   justification for any deviations from the protocol |     🗷 | Yes Partial Yes No |  |
|  | Comments: None | | | |  |
| **3. Did the review authors explain their selection of the study designs for inclusion in the review?** | | | | | |
|  | For Yes, the review should satisfy ONE of the following:   *Explanation for* including only RCTs   OR *Explanation for* including only NRSI   OR *Explanation for* including both RCTs and NRSI | | 🗷 | Yes No |  |
|  | Comments: They stated that they searched for all studies addressing prevalence (under Data Source). | | | |  |
| **4. Did the review authors use a comprehensive literature search strategy?** | | | | | |
|  | For Partial Yes (all the following): | For Yes, should also have (all the following):   searched the reference lists / bibliographies of included studies   searched trial/study registries   included/consulted content experts in the field  🗷 where relevant, searched for grey literature  🗷 conducted search within 24 months of completion of the review |  |  |  |
|  | 🗷 searched at least 2 databases (relevant to research question)  🗷 provided key word and/or search strategy   justified publication restrictions |  |     🗷 | Yes Partial Yes No |  |
|  | (e.g. language) |  |  |  |  |
|  | Comments: Restriction to English not justified, which is important given early data in Chinese. | | | |  |
|  | **5. Did the review authors perform study selection in duplicate?** | |  | |  |
|  | For Yes, either ONE of the following:   at least two reviewers independently agreed on selection of eligible studies and achieved consensus on which studies to include   OR two reviewers selected a sample of eligible studies and achieved good agreement (at least 80 percent), with the remainder selected by one reviewer. | |   🗷 | Yes No |  |
|  | Comments: Not reported | | | |  |

| **6. Did the review authors perform data extraction in duplicate?** | | | |
| --- | --- | --- | --- |
| For Yes, either ONE of the following:   at least two reviewers achieved consensus on which data to extract from included studies   OR two reviewers extracted data from a sample of eligible studies and achieved good agreement (at least 80 percent), with the remainder extracted by one reviewer. | | |  Yes  🗷 No |
| Comments: None | | | |
| **7. Did the review authors provide a list of excluded studies and justify the exclusions?** | | | |
|  | For Partial Yes:   provided a list of all potentially relevant studies that were read in full-text form but excluded from the review | For Yes, must also have:   Justified the exclusion from the review of each potentially relevant study |  Yes   Partial Yes  🗷 No |
|  | Comments: They gave the number of excluded studies and justified the exclusion but did not list them. | | |
| **8. Did the review authors describe the included studies in adequate detail?** | | | |
|  | For Partial Yes (ALL the following):  🗷 described populations  n/a described interventions  n/a described comparators  🗷 described outcomes  🗷 described research designs | For Yes, should also have ALL the following:  🗷 described population in detail  n/a described intervention in detail (including doses where relevant)  n/a described comparator in detail (including doses where relevant)  🗷 described study’s setting   timeframe for follow-up |  Yes  🗷 Partial Yes   No |
|  | Comments: These were not intervention studies. | | |
| **9. Did the review authors use a satisfactory technique for assessing the risk of bias (RoB) in individual studies that were included in the review?** | | | |
|  | **RCTs**  For Partial Yes, must have assessed RoB from   unconcealed allocation, *and*   lack of blinding of patients and assessors when assessing outcomes (unnecessary for objective outcomes such as all-cause mortality) | For Yes, must also have assessed RoB from:   allocation sequence that was not truly random, *and*   selection of the reported result from among multiple measurements or analyses of a specified outcome |  Yes   Partial Yes   No  🗷 Includes only NRSI |
|  | **NRSI**  For Partial Yes, must have assessed RoB:   from confounding, *and*   from selection bias | For Yes, must also have assessed RoB:   methods used to ascertain exposures and outcomes, *and*   selection of the reported result from among multiple measurements or analyses of a specified | 🗷 Yes   Partial Yes   No  Includes only RCTs |
| Comments: They stated they followed Cochrane’s RoB tool. | | | |

| **10. Did the review authors report outcomes on the sources of funding for the studies included in the review?** | | | | | | |
| --- | --- | --- | --- | --- | --- | --- |
| For Yes:   Must have reported on the sources of funding for individual studies included in the review.  Note: Reporting that the reviewers looked for this information but it was not reported by study authors also qualifies. | | | |  Yes  🗷 No |  |  |
| Comments: None | | | |  |  |  |
| **11. If meta-analysis was performed did the review authors use appropriate methods for statistical combination of results?** | | | | | | |
|  | **RCTs**  For Yes:   The authors justified combining the data in a meta-analysis   AND they used an appropriate weighted technique to combine study results and adjusted for heterogeneity if present.   AND investigated the causes of any heterogeneity | |  Yes   No   No meta-analysis conducted | | |  |
|  | **For NRSI**  For Yes:  🗷 The authors justified combining the data in a meta-analysis  🗷 AND they used an appropriate weighted technique to combine study results, adjusting for heterogeneity if present   AND they statistically combined effect estimates from NRSI that were adjusted for confounding, rather than combining raw data, or justified combining raw data when adjusted effect estimates were not available   AND they reported separate summary estimates for RCTs and NRSI separately when both were included in the review | | 🗷 Yes   No   No meta-analysis conducted | | |  |
|  | Comments: The meta-analyses look appropriate for a prevalence review, and it seems like some of the criteria are not relevant for that reason. | | | | |  |
| **12. If meta-analysis was performed, did the review authors assess the potential impact of RoB in individual studies on the results of the meta-analysis or other evidence synthesis?** | | | | | | |
|  | For Yes:   included only low risk of bias RCTs   OR, if the pooled estimate was based on RCTs and/or NRSI at variable RoB, the authors performed analyses to investigate possible impact of RoB on summary estimates of effect. | |  Yes  🗷 No   No meta-analysis conducted | | |  |
|  | Comments: While they stated they used Cochrane’s RoB, they did not discuss the results of this anywhere. | | | | |  |
| **13. Did the review authors account for RoB in individual studies when interpreting/ discussing the results of the review?** | | | | | | |
|  | For Yes:   included only low risk of bias RCTs   OR, if RCTs with moderate or high RoB, or NRSI were included the review provided a discussion of the likely impact of RoB on the results | |  Yes  🗷 No | | |  |
|  | Comments: While they stated they used Cochrane’s RoB, they did not discuss the results of this anywhere. | | | | |  |
| **14. Did the review authors provide a satisfactory explanation for, and discussion of, any heterogeneity observed in the results of the review?** | | | | | | |
|  | For Yes:   There was no significant heterogeneity in the results  🗷 OR if heterogeneity was present the authors performed an investigation of sources of any heterogeneity in the results and discussed the impact of this on the results of the review | | 🗷 Yes   No | | |  |
|  | Comments: None | | | | |  |
| **15. If they performed quantitative synthesis did the review authors carry out an adequate investigation of publication bias (small study bias) and discuss its likely impact on the results of the review?** | | | | | | |
|  | For Yes:  🗷 performed graphical or statistical tests for publication bias and discussed the likelihood and magnitude of impact of publication bias | | 🗷 Yes   No   No meta-analysis conducted | | |  |
|  | Comments: None | | | | |  |
| **16. Did the review authors report any potential sources of conflict of interest, including any funding they received for conducting the review?** | | | | | | |
|  | For Yes:   The authors reported no competing interests OR  🗷 The authors described their funding sources and how they managed potential conflicts of interest | 🗷 Yes   No | | | |  |
|  | Comments: None | | | | |  |

**To cite this tool:** Shea BJ, Reeves BC, Wells G, Thuku M, Hamel C, Moran J, Moher D, Tugwell P, Welch V, Kristjansson E, Henry DA. AMSTAR 2: a critical appraisal tool for systematic reviews that include randomised or non-randomised studies of healthcare interventions, or both. BMJ. 2017 Sep 21;358:j4008.

# Li LQ et al.

| **1. Did the research questions and inclusion criteria for the review include the components of PICO?** | | | | | |
| --- | --- | --- | --- | --- | --- |
| For Yes:   🗷 Population  n/a Intervention  n/a Comparator group  🗷 Outcome | | Optional (recommended)   Timeframe for follow-up | 🗷 | Yes  No |  |
|  Comments: C not relevant for case studies, nor Intervention. | | | | |  |
| **2. Did the report of the review contain an explicit statement that the review methods were established prior to the conduct of the review and did the report justify any significant deviations from the protocol?** | | | | | |
|  | For Partial Yes:  The authors state that they had a written protocol or guide that included ALL the following:   review question(s)   a search strategy   inclusion/exclusion criteria   a risk of bias assessment | For Yes:  As for partial yes, plus the protocol should be registered and should also have specified:   a meta-analysis/synthesis plan, if appropriate, *and*   a plan for investigating causes of heterogeneity   justification for any deviations from the protocol |     🗷 | Yes Partial Yes No |  |
|  | Comments: No protocol is mentioned. | | | |  |
| **3. Did the review authors explain their selection of the study designs for inclusion in the review?** | | | | | |
|  | For Yes, the review should satisfy ONE of the following:   *Explanation for* including only RCTs   OR *Explanation for* including only NRSI   OR *Explanation for* including both RCTs and NRSI | | 🗷   | Yes No |  |
|  | Comments: They stated that they searched for all case studies and that RCTs were not available yet. We inferred that this criterion was met. | | | |  |
| **4. Did the review authors use a comprehensive literature search strategy?** | | | | | |
|  | For Partial Yes (all the following): | For Yes, should also have (all the following):  🗷 searched the reference lists / bibliographies of included studies   searched trial/study registries   included/consulted content experts in the field   where relevant, searched for grey literature  🗷 conducted search within 24 months of completion of the review |  |  |  |
|  | 🗷 searched at least 2 databases (relevant to research question)  🗷 provided key word and/or search strategy   justified publication restrictions |  |   🗷   | Yes Partial Yes No |  |
|  | (e.g. language) |  |  |  |  |
|  | Comments: Language restrictions were not mentioned. | | | |  |
|  | **5. Did the review authors perform study selection in duplicate?** | |  | |  |
|  | For Yes, either ONE of the following:  🗷 at least two reviewers independently agreed on selection of eligible studies and achieved consensus on which studies to include   OR two reviewers selected a sample of eligible studies and achieved good agreement (at least 80 percent), with the remainder selected by one reviewer. | | 🗷 | Yes No |  |
|  | Comments: None | | | |  |

| **6. Did the review authors perform data extraction in duplicate?** | | | |
| --- | --- | --- | --- |
| For Yes, either ONE of the following:  🗷 at least two reviewers achieved consensus on which data to extract from included studies   OR two reviewers extracted data from a sample of eligible studies and achieved good agreement (at least 80 percent), with the remainder extracted by one reviewer. | | | 🗷 Yes   No |
| Comments: None | | | |
| **7. Did the review authors provide a list of excluded studies and justify the exclusions?** | | | |
|  | For Partial Yes:   provided a list of all potentially relevant studies that were read in full-text form but excluded from the review | For Yes, must also have:   Justified the exclusion from the review of each potentially relevant study |  Yes   Partial Yes  🗷 No |
|  | Comments: They gave the number of excluded studies and justified their exclusion but did not list all the references for these. | | |
| **8. Did the review authors describe the included studies in adequate detail?** | | | |
|  | For Partial Yes (ALL the following):  🗷 described populations  n/a described interventions  n/a described comparators  🗷 described outcomes  🗷 described research designs | For Yes, should also have ALL the following:  🗷 described population in detail  n/a described intervention in detail (including doses where relevant)  n/a described comparator in detail (including doses where relevant)  🗷 described study’s setting   timeframe for follow-up |  Yes  🗷 Partial Yes   No |
|  | Comments: Intervention and Comparator not relevant here. | | |
| **9. Did the review authors use a satisfactory technique for assessing the risk of bias (RoB) in individual studies that were included in the review?** | | | |
|  | **RCTs**  For Partial Yes, must have assessed RoB from   unconcealed allocation, *and*   lack of blinding of patients and assessors when assessing outcomes (unnecessary for objective outcomes such as all-cause mortality) | For Yes, must also have assessed RoB from:   allocation sequence that was not truly random, *and*   selection of the reported result from among multiple measurements or analyses of a specified outcome |  Yes   Partial Yes   No   Includes only NRSI |
|  | **NRSI**  For Partial Yes, must have assessed RoB:  🗷 from confounding, *and*  🗷 from selection bias | For Yes, must also have assessed RoB:  🗷 methods used to ascertain exposures and outcomes, *and*  🗷 selection of the reported result from among multiple measurements or analyses of a specified outcome | 🗷 Yes   Partial Yes   No  Includes only RCTs |
| Comments: They used the MINORS tool. | | | |

| **10. Did the review authors report outcomes on the sources of funding for the studies included in the review?** | | | | | | |
| --- | --- | --- | --- | --- | --- | --- |
| For Yes:   Must have reported on the sources of funding for individual studies included in the review.  Note: Reporting that the reviewers looked for this information but it was not reported by study authors also qualifies. | | | |  Yes  🗷 No |  |  |
| Comments: None | | | |  |  |  |
| **11. If meta-analysis was performed did the review authors use appropriate methods for statistical combination of results?** | | | | | | |
|  | **RCTs**  For Yes:   The authors justified combining the data in a meta-analysis   AND they used an appropriate weighted technique to combine study results and adjusted for heterogeneity if present.   AND investigated the causes of any heterogeneity | |  Yes   No   No meta-analysis conducted | | |  |
|  | **For NRSI**  For Yes:  🗷 The authors justified combining the data in a meta-analysis  🗷 AND they used an appropriate weighted technique to combine study results, adjusting for heterogeneity if present  🗷 AND they statistically combined effect estimates from NRSI that were adjusted for confounding, rather than combining raw data, or justified combining raw data when adjusted effect estimates were not available   AND they reported separate summary estimates for RCTs and NRSI separately when both were included in the review | | 🗷 Yes   No   No meta-analysis conducted | | |  |
|  | Comments: None | | | | |  |
| **12. If meta-analysis was performed, did the review authors assess the potential impact of RoB in individual studies on the results of the meta-analysis or other evidence synthesis?** | | | | | | |
|  | For Yes:   included only low risk of bias RCTs  🗷 OR, if the pooled estimate was based on RCTs and/or NRSI at variable RoB, the authors performed analyses to investigate possible impact of RoB on summary estimates of effect. | | 🗷 Yes   No   No meta-analysis conducted | | |  |
|  | Comments: None | | | | |  |
| **13. Did the review authors account for RoB in individual studies when interpreting/ discussing the results of the review?** | | | | | | |
|  | For Yes:   included only low risk of bias RCTs  🗷 OR, if RCTs with moderate or high RoB, or NRSI were included the review provided a discussion of the likely impact of RoB on the results | | 🗷 Yes   No | | |  |
|  | Comments: None | | | | |  |
| **14. Did the review authors provide a satisfactory explanation for, and discussion of, any heterogeneity observed in the results of the review?** | | | | | | |
|  | For Yes:  🗷 There was no significant heterogeneity in the results   OR if heterogeneity was present the authors performed an investigation of sources of any heterogeneity in the results and discussed the impact of this on the results of the review | | 🗷 Yes   No | | |  |
|  | Comments: None | | | | |  |
| **15. If they performed quantitative synthesis did the review authors carry out an adequate investigation of publication bias (small study bias) and discuss its likely impact on the results of the review?** | | | | | | |
|  | For Yes:  🗷 performed graphical or statistical tests for publication bias and discussed the likelihood and magnitude of impact of publication bias | | 🗷 Yes   No   No meta-analysis conducted | | |  |
|  | Comments: None | | | | |  |
| **16. Did the review authors report any potential sources of conflict of interest, including any funding they received for conducting the review?** | | | | | | |
|  | For Yes:   The authors reported no competing interests OR  🗷 The authors described their funding sources and how they managed potential conflicts of interest | 🗷 Yes   No | | | |  |
|  | Comments: None | | | | |  |

**To cite this tool:** Shea BJ, Reeves BC, Wells G, Thuku M, Hamel C, Moran J, Moher D, Tugwell P, Welch V, Kristjansson E, Henry DA. AMSTAR 2: a critical appraisal tool for systematic reviews that include randomised or non-randomised studies of healthcare interventions, or both. BMJ. 2017 Sep 21;358:j4008.

# Lippi et al. (Active smoking…)

| **1. Did the research questions and inclusion criteria for the review include the components of PICO?** | | | | | |
| --- | --- | --- | --- | --- | --- |
| For Yes:   🗷 Population  n/a Intervention  n/a Comparator group  🗷 Outcome | | Optional (recommended)   Timeframe for follow-up | 🗷 | Yes  No |  |
|  Comments: As this was a SR of non-intervention studies, I-intervention and C-comparison were not applicable. | | | | |  |
| **2. Did the report of the review contain an explicit statement that the review methods were established prior to the conduct of the review and did the report justify any significant deviations from the protocol?** | | | | | |
|  | For Partial Yes:  The authors state that they had a written protocol or guide that included ALL the following:   review question(s)   a search strategy   inclusion/exclusion criteria   a risk of bias assessment | For Yes:  As for partial yes, plus the protocol should be registered and should also have specified:   a meta-analysis/synthesis plan, if appropriate, *and*   a plan for investigating causes of heterogeneity   justification for any deviations from the protocol |     🗷 | Yes Partial Yes No |  |
|  | Comments: None | | | |  |
| **3. Did the review authors explain their selection of the study designs for inclusion in the review?** | | | | | |
|  | For Yes, the review should satisfy ONE of the following:   *Explanation for* including only RCTs   OR *Explanation for* including only NRSI   OR *Explanation for* including both RCTs and NRSI | |   🗷 | Yes No |  |
|  | Comments: None | | | |  |
| **4. Did the review authors use a comprehensive literature search strategy?** | | | | | |
|  | For Partial Yes (all the following): | For Yes, should also have (all the following):  🗷 searched the reference lists / bibliographies of included studies   searched trial/study registries  🗷 included/consulted content experts in the field   where relevant, searched for grey literature  🗷 conducted search within 24 months of completion of the review |  |  |  |
|  | 🗷 searched at least 2 databases (relevant to research question)  🗷 provided key word and/or search strategy  🗷 justified publication restrictions |  |   🗷 | Yes Partial Yes No |  |
|  | (e.g. language) |  |  |  |  |
|  | Comments: No language restriction. | | | |  |
|  | **5. Did the review authors perform study selection in duplicate?** | |  | |  |
|  | For Yes, either ONE of the following:   at least two reviewers independently agreed on selection of eligible studies and achieved consensus on which studies to include   OR two reviewers selected a sample of eligible studies and achieved good agreement (at least 80 percent), with the remainder selected by one reviewer. | |   🗷 | Yes No |  |
|  | Comments: None | | | |  |

| **6. Did the review authors perform data extraction in duplicate?** | | | |
| --- | --- | --- | --- |
| For Yes, either ONE of the following:   at least two reviewers achieved consensus on which data to extract from included studies   OR two reviewers extracted data from a sample of eligible studies and achieved good agreement (at least 80 percent), with the remainder extracted by one reviewer. | | |  Yes  🗷 No |
| Comments: None | | | |
| **7. Did the review authors provide a list of excluded studies and justify the exclusions?** | | | |
|  | For Partial Yes:   provided a list of all potentially relevant studies that were read in full-text form but excluded from the review | For Yes, must also have:   Justified the exclusion from the review of each potentially relevant study |  Yes   Partial Yes  🗷 No |
|  | Comments: They gave the numbers of studies excluded and the reasons for exclusion, but not a list. | | |
| **8. Did the review authors describe the included studies in adequate detail?** | | | |
|  | For Partial Yes (ALL the following):  🗷 described populations  n/a described interventions  n/a described comparators  🗷 described outcomes   described research designs | For Yes, should also have ALL the following:  🗷 described population in detail  n/a described intervention in detail (including doses where relevant)  n/a described comparator in detail (including doses where relevant)  🗷 described study’s setting   timeframe for follow-up |  Yes   Partial Yes  🗷 No |
|  | Comments: They did not describe the types of cases nor how the COVID-19 diagnosis was validated. | | |
| **9. Did the review authors use a satisfactory technique for assessing the risk of bias (RoB) in individual studies that were included in the review?** | | | |
|  | **RCTs**  For Partial Yes, must have assessed RoB from   unconcealed allocation, *and*   lack of blinding of patients and assessors when assessing outcomes (unnecessary for objective outcomes such as all-cause mortality) | For Yes, must also have assessed RoB from:   allocation sequence that was not truly random, *and*   selection of the reported result from among multiple measurements or analyses of a specified outcome |  Yes   Partial Yes   No   Includes only NRSI |
|  | **NRSI**  For Partial Yes, must have assessed RoB:   from confounding, *and*   from selection bias | For Yes, must also have assessed RoB:   methods used to ascertain exposures and outcomes, *and*   selection of the reported result from among multiple measurements or analyses of a specified outcome |  Yes   Partial Yes  🗷 No  Includes only RCTs |
| Comments: None | | | |

| **10. Did the review authors report outcomes on the sources of funding for the studies included in the review?** | | | | | | |
| --- | --- | --- | --- | --- | --- | --- |
| For Yes:   Must have reported on the sources of funding for individual studies included in the review.  Note: Reporting that the reviewers looked for this information but it was not reported by study authors also qualifies. | | | |  Yes  🗷 No |  |  |
| Comments: None | | | |  |  |  |
| **11. If meta-analysis was performed did the review authors use appropriate methods for statistical combination of results?** | | | | | | |
|  | **RCTs**  For Yes:   The authors justified combining the data in a meta-analysis   AND they used an appropriate weighted technique to combine study results and adjusted for heterogeneity if present.   AND investigated the causes of any heterogeneity | |  Yes   No   No meta-analysis conducted | | |  |
|  | **For NRSI**  For Yes:  🗷 The authors justified combining the data in a meta-analysis  🗷 AND they used an appropriate weighted technique to combine study results, adjusting for heterogeneity if present   AND they statistically combined effect estimates from NRSI that were adjusted for confounding, rather than combining raw data, or justified combining raw data when adjusted effect estimates were not available   AND they reported separate summary estimates for RCTs and NRSI separately when both were included in the review | |  Yes  🗷 No   No meta-analysis conducted | | |  |
|  | Comments: We consider it was justified by meeting the inclusion criteria. | | | | |  |
| **12. If meta-analysis was performed, did the review authors assess the potential impact of RoB in individual studies on the results of the meta-analysis or other evidence synthesis?** | | | | | | |
|  | For Yes:   included only low risk of bias RCTs   OR, if the pooled estimate was based on RCTs and/or NRSI at variable RoB, the authors performed analyses to investigate possible impact of RoB on summary estimates of effect. | |  Yes  🗷 No   No meta-analysis conducted | | |  |
|  | Comments: None | | | | |  |
| **13. Did the review authors account for RoB in individual studies when interpreting/ discussing the results of the review?** | | | | | | |
|  | For Yes:   included only low risk of bias RCTs   OR, if RCTs with moderate or high RoB, or NRSI were included the review provided a discussion of the likely impact of RoB on the results | |  Yes  🗷 No | | |  |
|  | Comments: None | | | | |  |
| **14. Did the review authors provide a satisfactory explanation for, and discussion of, any heterogeneity observed in the results of the review?** | | | | | | |
|  | For Yes:   There was no significant heterogeneity in the results   OR if heterogeneity was present the authors performed an investigation of sources of any heterogeneity in the results and discussed the impact of this on the results of the review | |  Yes  🗷 No | | |  |
|  | Comments: None | | | | |  |
| **15. If they performed quantitative synthesis did the review authors carry out an adequate investigation of publication bias (small study bias) and discuss its likely impact on the results of the review?** | | | | | | |
|  | For Yes:   performed graphical or statistical tests for publication bias and discussed the likelihood and magnitude of impact of publication bias | |  Yes  🗷 No   No meta-analysis conducted | | |  |
|  | Comments: None | | | | |  |
| **16. Did the review authors report any potential sources of conflict of interest, including any funding they received for conducting the review?** | | | | | | |
|  | For Yes:  🗷 The authors reported no competing interests OR   The authors described their funding sources and how they managed potential conflicts of interest | 🗷 Yes   No | | | |  |
|  | Comments: None | | | | |  |

**To cite this tool:** Shea BJ, Reeves BC, Wells G, Thuku M, Hamel C, Moran J, Moher D, Tugwell P, Welch V, Kristjansson E, Henry DA. AMSTAR 2: a critical appraisal tool for systematic reviews that include randomised or non-randomised studies of healthcare interventions, or both. BMJ. 2017 Sep 21;358:j4008.

# Lippi et al. (Cardiac troponin…)

| **1. Did the research questions and inclusion criteria for the review include the components of PICO?** | | | | | |
| --- | --- | --- | --- | --- | --- |
| For Yes:   🗷 Population  n/a Intervention  n/a Comparator group  🗷 Outcome | | Optional (recommended)   Timeframe for follow-up | 🗷 | Yes  No |  |
|  Comments: As this was a SR of non-intervention studies, I-intervention and C-comparison were not applicable. | | | | |  |
| **2. Did the report of the review contain an explicit statement that the review methods were established prior to the conduct of the review and did the report justify any significant deviations from the protocol?** | | | | | |
|  | For Partial Yes:  The authors state that they had a written protocol or guide that included ALL the following:   review question(s)   a search strategy   inclusion/exclusion criteria   a risk of bias assessment | For Yes:  As for partial yes, plus the protocol should be registered and should also have specified:   a meta-analysis/synthesis plan, if appropriate, *and*   a plan for investigating causes of heterogeneity   justification for any deviations from the protocol |     🗷 | Yes Partial Yes No |  |
|  | Comments: None | | | |  |
| **3. Did the review authors explain their selection of the study designs for inclusion in the review?** | | | | | |
|  | For Yes, the review should satisfy ONE of the following:   *Explanation for* including only RCTs   OR *Explanation for* including only NRSI   OR *Explanation for* including both RCTs and NRSI | |   🗷 | Yes No |  |
|  | Comments: None | | | |  |
| **4. Did the review authors use a comprehensive literature search strategy?** | | | | | |
|  | For Partial Yes (all the following): | For Yes, should also have (all the following):  🗷 searched the reference lists / bibliographies of included studies   searched trial/study registries  🗷 included/consulted content experts in the field   where relevant, searched for grey literature  🗷 conducted search within 24 months of completion of the review |  |  |  |
|  | 🗷 searched at least 2 databases (relevant to research question)  🗷 provided key word and/or search strategy  🗷 justified publication restrictions |  |   🗷 | Yes Partial Yes No |  |
|  | (e.g. language) |  |  |  |  |
|  | Comments: No language restriction. | | | |  |
|  | **5. Did the review authors perform study selection in duplicate?** | |  | |  |
|  | For Yes, either ONE of the following:   at least two reviewers independently agreed on selection of eligible studies and achieved consensus on which studies to include   OR two reviewers selected a sample of eligible studies and achieved good agreement (at least 80 percent), with the remainder selected by one reviewer. | |   🗷 | Yes No |  |
|  | Comments: None | | | |  |

| **6. Did the review authors perform data extraction in duplicate?** | | | |
| --- | --- | --- | --- |
| For Yes, either ONE of the following:   at least two reviewers achieved consensus on which data to extract from included studies   OR two reviewers extracted data from a sample of eligible studies and achieved good agreement (at least 80 percent), with the remainder extracted by one reviewer. | | |  Yes  🗷 No |
| Comments: None | | | |
| **7. Did the review authors provide a list of excluded studies and justify the exclusions?** | | | |
|  | For Partial Yes:   provided a list of all potentially relevant studies that were read in full-text form but excluded from the review | For Yes, must also have:   Justified the exclusion from the review of each potentially relevant study |  Yes   Partial Yes  🗷 No |
|  | Comments: They gave the numbers of studies excluded and the reasons for exclusion, but not a list. | | |
| **8. Did the review authors describe the included studies in adequate detail?** | | | |
|  | For Partial Yes (ALL the following):   described populations  n/a described interventions  n/a described comparators  🗷 described outcomes   described research designs | For Yes, should also have ALL the following:   described population in detail  n/a described intervention in detail (including doses where relevant)  n/a described comparator in detail (including doses where relevant)  🗷 described study’s setting   timeframe for follow-up |  Yes   Partial Yes  🗷 No |
|  | Comments: Gave the sample size, but no details on age, gender, etc. | | |
| **9. Did the review authors use a satisfactory technique for assessing the risk of bias (RoB) in individual studies that were included in the review?** | | | |
|  | **RCTs**  For Partial Yes, must have assessed RoB from   unconcealed allocation, *and*   lack of blinding of patients and assessors when assessing outcomes (unnecessary for objective outcomes such as all-cause mortality) | For Yes, must also have assessed RoB from:   allocation sequence that was not truly random, *and*   selection of the reported result from among multiple measurements or analyses of a specified outcome |  Yes   Partial Yes   No   Includes only NRSI |
|  | **NRSI**  For Partial Yes, must have assessed RoB:   from confounding, *and*   from selection bias | For Yes, must also have assessed RoB:   methods used to ascertain exposures and outcomes, *and*   selection of the reported result from among multiple measurements or analyses of a specified outcome |  Yes   Partial Yes  🗷 No  Includes only RCTs |
| Comments: None | | | |

| **10. Did the review authors report outcomes on the sources of funding for the studies included in the review?** | | | | | | |
| --- | --- | --- | --- | --- | --- | --- |
| For Yes:   Must have reported on the sources of funding for individual studies included in the review.  Note: Reporting that the reviewers looked for this information but it was not reported by study authors also qualifies. | | | |  Yes  🗷 No |  |  |
| Comments: None | | | |  |  |  |
| **11. If meta-analysis was performed did the review authors use appropriate methods for statistical combination of results?** | | | | | | |
|  | **RCTs**  For Yes:   The authors justified combining the data in a meta-analysis   AND they used an appropriate weighted technique to combine study results and adjusted for heterogeneity if present.   AND investigated the causes of any heterogeneity | |  Yes   No   No meta-analysis conducted | | |  |
|  | **For NRSI**  For Yes:  🗷 The authors justified combining the data in a meta-analysis  🗷 AND they used an appropriate weighted technique to combine study results, adjusting for heterogeneity if present   AND they statistically combined effect estimates from NRSI that were adjusted for confounding, rather than combining raw data, or justified combining raw data when adjusted effect estimates were not available   AND they reported separate summary estimates for RCTs and NRSI separately when both were included in the review | |  Yes  🗷 No   No meta-analysis conducted | | |  |
|  | Comments: We consider it was justified by the studies meeting the inclusion criteria. | | | | |  |
| **12. If meta-analysis was performed, did the review authors assess the potential impact of RoB in individual studies on the results of the meta-analysis or other evidence synthesis?** | | | | | | |
|  | For Yes:   included only low risk of bias RCTs   OR, if the pooled estimate was based on RCTs and/or NRSI at variable RoB, the authors performed analyses to investigate possible impact of RoB on summary estimates of effect. | |  Yes  🗷 No   No meta-analysis conducted | | |  |
|  | Comments: None | | | | |  |
| **13. Did the review authors account for RoB in individual studies when interpreting/ discussing the results of the review?** | | | | | | |
|  | For Yes:   included only low risk of bias RCTs   OR, if RCTs with moderate or high RoB, or NRSI were included the review provided a discussion of the likely impact of RoB on the results | |  Yes  🗷 No | | |  |
|  | Comments: None | | | | |  |
| **14. Did the review authors provide a satisfactory explanation for, and discussion of, any heterogeneity observed in the results of the review?** | | | | | | |
|  | For Yes:   There was no significant heterogeneity in the results   OR if heterogeneity was present the authors performed an investigation of sources of any heterogeneity in the results and discussed the impact of this on the results of the review | |  Yes  🗷 No | | |  |
|  | Comments: I^2^ = 98%, but they did not take it into account in discussions. | | | | |  |
| **15. If they performed quantitative synthesis did the review authors carry out an adequate investigation of publication bias (small study bias) and discuss its likely impact on the results of the review?** | | | | | | |
|  | For Yes:   performed graphical or statistical tests for publication bias and discussed the likelihood and magnitude of impact of publication bias | |  Yes  🗷 No   No meta-analysis conducted | | |  |
|  | Comments: None | | | | |  |
| **16. Did the review authors report any potential sources of conflict of interest, including any funding they received for conducting the review?** | | | | | | |
|  | For Yes:  🗷 The authors reported no competing interests OR   The authors described their funding sources and how they managed potential conflicts of interest | 🗷 Yes   No | | | |  |
|  | Comments: None | | | | |  |

**To cite this tool:** Shea BJ, Reeves BC, Wells G, Thuku M, Hamel C, Moran J, Moher D, Tugwell P, Welch V, Kristjansson E, Henry DA. AMSTAR 2: a critical appraisal tool for systematic reviews that include randomised or non-randomised studies of healthcare interventions, or both. BMJ. 2017 Sep 21;358:j4008.

# Lippi et al. (procalcitonin)

| **1. Did the research questions and inclusion criteria for the review include the components of PICO?** | | | | | |
| --- | --- | --- | --- | --- | --- |
| For Yes:   🗷 Population  n/a Intervention  n/a Comparator group  🗷 Outcome | | Optional (recommended)   Timeframe for follow-up | 🗷 | Yes  No |  |
|  Comments: As this was a SR of non-intervention studies, I-intervention and C-comparison were not applicable. O was reported procalcitonin values with clinically validated severe disease. | | | | |  |
| **2. Did the report of the review contain an explicit statement that the review methods were established prior to the conduct of the review and did the report justify any significant deviations from the protocol?** | | | | | |
|  | For Partial Yes:  The authors state that they had a written protocol or guide that included ALL the following:   review question(s)   a search strategy   inclusion/exclusion criteria   a risk of bias assessment | For Yes:  As for partial yes, plus the protocol should be registered and should also have specified:   a meta-analysis/synthesis plan, if appropriate, *and*   a plan for investigating causes of heterogeneity   justification for any deviations from the protocol |     🗷 | Yes Partial Yes No |  |
|  | Comments: None | | | |  |
| **3. Did the review authors explain their selection of the study designs for inclusion in the review?** | | | | | |
|  | For Yes, the review should satisfy ONE of the following:   *Explanation for* including only RCTs   OR *Explanation for* including only NRSI   OR *Explanation for* including both RCTs and NRSI | |   🗷 | Yes No |  |
|  | Comments: None | | | |  |
| **4. Did the review authors use a comprehensive literature search strategy?** | | | | | |
|  | For Partial Yes (all the following): | For Yes, should also have (all the following):  🗷 searched the reference lists / bibliographies of included studies   searched trial/study registries  🗷 included/consulted content experts in the field   where relevant, searched for grey literature  🗷 conducted search within 24 months of completion of the review |  |  |  |
|  | 🗷 searched at least 2 databases (relevant to research question)  🗷 provided key word and/or search strategy  🗷 justified publication restrictions |  |   🗷 | Yes Partial Yes No |  |
|  | (e.g. language) |  |  |  |  |
|  | Comments: No language restriction. | | | |  |
|  | **5. Did the review authors perform study selection in duplicate?** | |  | |  |
|  | For Yes, either ONE of the following:   at least two reviewers independently agreed on selection of eligible studies and achieved consensus on which studies to include   OR two reviewers selected a sample of eligible studies and achieved good agreement (at least 80 percent), with the remainder selected by one reviewer. | |   🗷 | Yes No |  |
|  | Comments: Stated that “authors” did selection, but not if it was independently or in duplicate. | | | |  |

| **6. Did the review authors perform data extraction in duplicate?** | | | |
| --- | --- | --- | --- |
| For Yes, either ONE of the following:   at least two reviewers achieved consensus on which data to extract from included studies   OR two reviewers extracted data from a sample of eligible studies and achieved good agreement (at least 80 percent), with the remainder extracted by one reviewer. | | |  Yes  🗷 No |
| Comments: None | | | |
| **7. Did the review authors provide a list of excluded studies and justify the exclusions?** | | | |
|  | For Partial Yes:   provided a list of all potentially relevant studies that were read in full-text form but excluded from the review | For Yes, must also have:   Justified the exclusion from the review of each potentially relevant study |  Yes   Partial Yes  🗷 No |
|  | Comments: They gave the numbers of studies excluded and the reasons for exclusion, but not a list. | | |
| **8. Did the review authors describe the included studies in adequate detail?** | | | |
|  | For Partial Yes (ALL the following):   described populations  n/a described interventions  n/a described comparators  🗷 described outcomes   described research designs | For Yes, should also have ALL the following:   described population in detail  n/a described intervention in detail (including doses where relevant)  n/a described comparator in detail (including doses where relevant)   described study’s setting   timeframe for follow-up |  Yes   Partial Yes  🗷 No |
|  | Comments: None | | |
| **9. Did the review authors use a satisfactory technique for assessing the risk of bias (RoB) in individual studies that were included in the review?** | | | |
|  | **RCTs**  For Partial Yes, must have assessed RoB from   unconcealed allocation, *and*   lack of blinding of patients and assessors when assessing outcomes (unnecessary for objective outcomes such as all-cause mortality) | For Yes, must also have assessed RoB from:   allocation sequence that was not truly random, *and*   selection of the reported result from among multiple measurements or analyses of a specified outcome |  Yes   Partial Yes   No   Includes only NRSI |
|  | **NRSI**  For Partial Yes, must have assessed RoB:   from confounding, *and*   from selection bias | For Yes, must also have assessed RoB:   methods used to ascertain exposures and outcomes, *and*   selection of the reported result from among multiple measurements or analyses of a specified outcome |  Yes   Partial Yes  🗷 No  Includes only RCTs |
| Comments: None | | | |

| **10. Did the review authors report outcomes on the sources of funding for the studies included in the review?** | | | | | | |
| --- | --- | --- | --- | --- | --- | --- |
| For Yes:   Must have reported on the sources of funding for individual studies included in the review.  Note: Reporting that the reviewers looked for this information but it was not reported by study authors also qualifies. | | | |  Yes  🗷 No |  |  |
| Comments: None | | | |  |  |  |
| **11. If meta-analysis was performed did the review authors use appropriate methods for statistical combination of results?** | | | | | | |
|  | **RCTs**  For Yes:   The authors justified combining the data in a meta-analysis   AND they used an appropriate weighted technique to combine study results and adjusted for heterogeneity if present.   AND investigated the causes of any heterogeneity | |  Yes   No   No meta-analysis conducted | | |  |
|  | **For NRSI**  For Yes:  🗷 The authors justified combining the data in a meta-analysis  🗷 AND they used an appropriate weighted technique to combine study results, adjusting for heterogeneity if present   AND they statistically combined effect estimates from NRSI that were adjusted for confounding, rather than combining raw data, or justified combining raw data when adjusted effect estimates were not available   AND they reported separate summary estimates for RCTs and NRSI separately when both were included in the review | |  Yes  🗷 No   No meta-analysis conducted | | |  |
|  | Comments: We consider it was justified by the studies meeting the inclusion criteria. | | | | |  |
| **12. If meta-analysis was performed, did the review authors assess the potential impact of RoB in individual studies on the results of the meta-analysis or other evidence synthesis?** | | | | | | |
|  | For Yes:   included only low risk of bias RCTs   OR, if the pooled estimate was based on RCTs and/or NRSI at variable RoB, the authors performed analyses to investigate possible impact of RoB on summary estimates of effect. | |  Yes  🗷 No   No meta-analysis conducted | | |  |
|  | Comments: None | | | | |  |
| **13. Did the review authors account for RoB in individual studies when interpreting/ discussing the results of the review?** | | | | | | |
|  | For Yes:   included only low risk of bias RCTs   OR, if RCTs with moderate or high RoB, or NRSI were included the review provided a discussion of the likely impact of RoB on the results | |  Yes  🗷 No | | |  |
|  | Comments: None | | | | |  |
| **14. Did the review authors provide a satisfactory explanation for, and discussion of, any heterogeneity observed in the results of the review?** | | | | | | |
|  | For Yes:  🗷 There was no significant heterogeneity in the results   OR if heterogeneity was present the authors performed an investigation of sources of any heterogeneity in the results and discussed the impact of this on the results of the review | | 🗷 Yes   No | | |  |
|  | Comments: I^2^ = 34% which they said was modest. | | | | |  |
| **15. If they performed quantitative synthesis did the review authors carry out an adequate investigation of publication bias (small study bias) and discuss its likely impact on the results of the review?** | | | | | | |
|  | For Yes:   performed graphical or statistical tests for publication bias and discussed the likelihood and magnitude of impact of publication bias | |  Yes  🗷 No   No meta-analysis conducted | | |  |
|  | Comments: None | | | | |  |
| **16. Did the review authors report any potential sources of conflict of interest, including any funding they received for conducting the review?** | | | | | | |
|  | For Yes:   The authors reported no competing interests OR   The authors described their funding sources and how they managed potential conflicts of interest |  Yes  🗷 No | | | |  |
|  | Comments: None | | | | |  |

**To cite this tool:** Shea BJ, Reeves BC, Wells G, Thuku M, Hamel C, Moran J, Moher D, Tugwell P, Welch V, Kristjansson E, Henry DA. AMSTAR 2: a critical appraisal tool for systematic reviews that include randomised or non-randomised studies of healthcare interventions, or both. BMJ. 2017 Sep 21;358:j4008.

# Lippi et al. (Thrombocytopenia…)

| **1. Did the research questions and inclusion criteria for the review include the components of PICO?** | | | | | |
| --- | --- | --- | --- | --- | --- |
| For Yes:   🗷 Population  n/a Intervention  n/a Comparator group  🗷 Outcome | | Optional (recommended)   Timeframe for follow-up | 🗷 | Yes  No |  |
|  Comments: As this was a SR of non-intervention studies, I-intervention and C-comparison were not applicable. O was reported platelet count with clinically validated severe disease. | | | | |  |
| **2. Did the report of the review contain an explicit statement that the review methods were established prior to the conduct of the review and did the report justify any significant deviations from the protocol?** | | | | | |
|  | For Partial Yes:  The authors state that they had a written protocol or guide that included ALL the following:   review question(s)   a search strategy   inclusion/exclusion criteria   a risk of bias assessment | For Yes:  As for partial yes, plus the protocol should be registered and should also have specified:   a meta-analysis/synthesis plan, if appropriate, *and*   a plan for investigating causes of heterogeneity   justification for any deviations from the protocol |     🗷 | Yes Partial Yes No |  |
|  | Comments: None | | | |  |
| **3. Did the review authors explain their selection of the study designs for inclusion in the review?** | | | | | |
|  | For Yes, the review should satisfy ONE of the following:   *Explanation for* including only RCTs   OR *Explanation for* including only NRSI   OR *Explanation for* including both RCTs and NRSI | |   🗷 | Yes No |  |
|  | Comments: None | | | |  |
| **4. Did the review authors use a comprehensive literature search strategy?** | | | | | |
|  | For Partial Yes (all the following): | For Yes, should also have (all the following):  🗷 searched the reference lists / bibliographies of included studies   searched trial/study registries  🗷 included/consulted content experts in the field   where relevant, searched for grey literature  🗷 conducted search within 24 months of completion of the review |  |  |  |
|  | 🗷 searched at least 2 databases (relevant to research question)  🗷 provided key word and/or search strategy  🗷 justified publication restrictions |  |   🗷 | Yes Partial Yes No |  |
|  | (e.g. language) |  |  |  |  |
|  | Comments: No language restriction. | | | |  |
|  | **5. Did the review authors perform study selection in duplicate?** | |  | |  |
|  | For Yes, either ONE of the following:  🗷 at least two reviewers independently agreed on selection of eligible studies and achieved consensus on which studies to include   OR two reviewers selected a sample of eligible studies and achieved good agreement (at least 80 percent), with the remainder selected by one reviewer. | | 🗷 | Yes No |  |
|  | Comments: Stated that “authors” did selection, but not if it was independently or in duplicate. | | | |  |

| **6. Did the review authors perform data extraction in duplicate?** | | | |
| --- | --- | --- | --- |
| For Yes, either ONE of the following:   at least two reviewers achieved consensus on which data to extract from included studies   OR two reviewers extracted data from a sample of eligible studies and achieved good agreement (at least 80 percent), with the remainder extracted by one reviewer. | | |  Yes  🗷 No |
| Comments: | | | |
| **7. Did the review authors provide a list of excluded studies and justify the exclusions?** | | | |
|  | For Partial Yes:   provided a list of all potentially relevant studies that were read in full-text form but excluded from the review | For Yes, must also have:   Justified the exclusion from the review of each potentially relevant study |  Yes   Partial Yes  🗷 No |
|  | Comments: They gave the numbers of studies excluded and the reasons for exclusion, but not a list. | | |
| **8. Did the review authors describe the included studies in adequate detail?** | | | |
|  | For Partial Yes (ALL the following):  🗷 described populations  n/a described interventions  n/a described comparators  🗷 described outcomes   described research designs | For Yes, should also have ALL the following:  🗷 described population in detail  n/a described intervention in detail (including doses where relevant)  n/a described comparator in detail (including doses where relevant)  🗷 described study’s setting   timeframe for follow-up |  Yes   Partial Yes  🗷 No |
|  | Comments: They stated that the research design was case studies but gave no details on how the cases were selected, etc. | | |
| **9. Did the review authors use a satisfactory technique for assessing the risk of bias (RoB) in individual studies that were included in the review?** | | | |
|  | **RCTs**  For Partial Yes, must have assessed RoB from   unconcealed allocation, *and*   lack of blinding of patients and assessors when assessing outcomes (unnecessary for objective outcomes such as all-cause mortality) | For Yes, must also have assessed RoB from:   allocation sequence that was not truly random, *and*   selection of the reported result from among multiple measurements or analyses of a specified outcome |  Yes   Partial Yes   No   Includes only NRSI |
|  | **NRSI**  For Partial Yes, must have assessed RoB:   from confounding, *and*   from selection bias | For Yes, must also have assessed RoB:   methods used to ascertain exposures and outcomes, *and*   selection of the reported result from among multiple measurements or analyses of a specified outcome |  Yes   Partial Yes  🗷 No  Includes only RCTs |
| Comments: None | | | |

| **10. Did the review authors report outcomes on the sources of funding for the studies included in the review?** | | | | | | |
| --- | --- | --- | --- | --- | --- | --- |
| For Yes:   Must have reported on the sources of funding for individual studies included in the review.  Note: Reporting that the reviewers looked for this information but it was not reported by study authors also qualifies. | | | |  Yes  🗷 No |  |  |
| Comments: None | | | |  |  |  |
| **11. If meta-analysis was performed did the review authors use appropriate methods for statistical combination of results?** | | | | | | |
|  | **RCTs**  For Yes:   The authors justified combining the data in a meta-analysis   AND they used an appropriate weighted technique to combine study results and adjusted for heterogeneity if present.   AND investigated the causes of any heterogeneity | |  Yes   No   No meta-analysis conducted | | |  |
|  | **For NRSI**  For Yes:  🗷 The authors justified combining the data in a meta-analysis  🗷 AND they used an appropriate weighted technique to combine study results, adjusting for heterogeneity if present   AND they statistically combined effect estimates from NRSI that were adjusted for confounding, rather than combining raw data, or justified combining raw data when adjusted effect estimates were not available   AND they reported separate summary estimates for RCTs and NRSI separately when both were included in the review | |  Yes  🗷 No   No meta-analysis conducted | | |  |
|  | Comments: We consider it was justified by the studies meeting the inclusion criteria. | | | | |  |
| **12. If meta-analysis was performed, did the review authors assess the potential impact of RoB in individual studies on the results of the meta-analysis or other evidence synthesis?** | | | | | | |
|  | For Yes:   included only low risk of bias RCTs   OR, if the pooled estimate was based on RCTs and/or NRSI at variable RoB, the authors performed analyses to investigate possible impact of RoB on summary estimates of effect. | |  Yes  🗷 No   No meta-analysis conducted | | |  |
|  | Comments: None | | | | |  |
| **13. Did the review authors account for RoB in individual studies when interpreting/ discussing the results of the review?** | | | | | | |
|  | For Yes:   included only low risk of bias RCTs   OR, if RCTs with moderate or high RoB, or NRSI were included the review provided a discussion of the likely impact of RoB on the results | |  Yes  🗷 No | | |  |
|  | Comments: None | | | | |  |
| **14. Did the review authors provide a satisfactory explanation for, and discussion of, any heterogeneity observed in the results of the review?** | | | | | | |
|  | For Yes:   There was no significant heterogeneity in the results  🗷 OR if heterogeneity was present the authors performed an investigation of sources of any heterogeneity in the results and discussed the impact of this on the results of the review | | 🗷 Yes   No | | |  |
|  | Comments: I^2^ = 92%, and they mentioned this as a limitation. They used subgroup analysis to investigate it but concluded this was limited by a lack of individual patient data. | | | | |  |
| **15. If they performed quantitative synthesis did the review authors carry out an adequate investigation of publication bias (small study bias) and discuss its likely impact on the results of the review?** | | | | | | |
|  | For Yes:   performed graphical or statistical tests for publication bias and discussed the likelihood and magnitude of impact of publication bias | |  Yes  🗷 No   No meta-analysis conducted | | |  |
|  | Comments: None | | | | |  |
| **16. Did the review authors report any potential sources of conflict of interest, including any funding they received for conducting the review?** | | | | | | |
|  | For Yes:  🗷 The authors reported no competing interests OR   The authors described their funding sources and how they managed potential conflicts of interest | 🗷 Yes   No | | | |  |
|  | Comments: None | | | | |  |

**To cite this tool:** Shea BJ, Reeves BC, Wells G, Thuku M, Hamel C, Moran J, Moher D, Tugwell P, Welch V, Kristjansson E, Henry DA. AMSTAR 2: a critical appraisal tool for systematic reviews that include randomised or non-randomised studies of healthcare interventions, or both. BMJ. 2017 Sep 21;358:j4008.

# Ludvigsson et al.

| **1. Did the research questions and inclusion criteria for the review include the components of PICO?** | | | | | |
| --- | --- | --- | --- | --- | --- |
| For Yes:   🗷 Population  n/a Intervention  n/a Comparator group  🗷 Outcome | | Optional (recommended)   Timeframe for follow-up | 🗷 | Yes  No |  |
|  Comments: As this was a SR of non-intervention studies, I-intervention and C-comparison were not applicable. Several O were described here. | | | | |  |
| **2. Did the report of the review contain an explicit statement that the review methods were established prior to the conduct of the review and did the report justify any significant deviations from the protocol?** | | | | | |
|  | For Partial Yes:  The authors state that they had a written protocol or guide that included ALL the following:   review question(s)   a search strategy   inclusion/exclusion criteria   a risk of bias assessment | For Yes:  As for partial yes, plus the protocol should be registered and should also have specified:   a meta-analysis/synthesis plan, if appropriate, *and*   a plan for investigating causes of heterogeneity   justification for any deviations from the protocol |     🗷 | Yes Partial Yes No |  |
|  | Comments: They stated they did not have pre-specified protocol. | | | |  |
| **3. Did the review authors explain their selection of the study designs for inclusion in the review?** | | | | | |
|  | For Yes, the review should satisfy ONE of the following:   *Explanation for* including only RCTs   OR *Explanation for* including only NRSI   OR *Explanation for* including both RCTs and NRSI | |   🗷 | Yes No |  |
|  | Comments: Not reported | | | |  |
| **4. Did the review authors use a comprehensive literature search strategy?** | | | | | |
|  | For Partial Yes (all the following): | For Yes, should also have (all the following):  🗷 searched the reference lists / bibliographies of included studies   searched trial/study registries  🗷 included/consulted content experts in the field   where relevant, searched for grey literature  🗷 conducted search within 24 months of completion of the review |  |  |  |
|  | 🗷 searched at least 2 databases (relevant to research question)  🗷 provided key word and/or search strategy  🗷 justified publication restrictions |  |   🗷   | Yes Partial Yes No |  |
|  | (e.g. language) |  |  |  |  |
|  | Comments: No language restrictions were applied, but they were unable to read the full text of some Chinese articles. | | | |  |
|  | **5. Did the review authors perform study selection in duplicate?** | |  | |  |
|  | For Yes, either ONE of the following:   at least two reviewers independently agreed on selection of eligible studies and achieved consensus on which studies to include   OR two reviewers selected a sample of eligible studies and achieved good agreement (at least 80 percent), with the remainder selected by one reviewer. | |   🗷 | Yes No |  |
|  | Comments: | | | |  |

| **6. Did the review authors perform data extraction in duplicate?** | | | |
| --- | --- | --- | --- |
| For Yes, either ONE of the following:   at least two reviewers achieved consensus on which data to extract from included studies   OR two reviewers extracted data from a sample of eligible studies and achieved good agreement (at least 80 percent), with the remainder extracted by one reviewer. | | |  Yes  🗷 No |
| Comments: None | | | |
| **7. Did the review authors provide a list of excluded studies and justify the exclusions?** | | | |
|  | For Partial Yes:   provided a list of all potentially relevant studies that were read in full-text form but excluded from the review | For Yes, must also have:   Justified the exclusion from the review of each potentially relevant study |  Yes   Partial Yes  🗷 No |
|  | Comments: None | | |
| **8. Did the review authors describe the included studies in adequate detail?** | | | |
|  | For Partial Yes (ALL the following):  🗷 described populations  n/a described interventions  n/a described comparators  🗷 described outcomes   described research designs | For Yes, should also have ALL the following:   described population in detail  n/a described intervention in detail (including doses where relevant)  n/a described comparator in detail (including doses where relevant)   described study’s setting   timeframe for follow-up |  Yes   Partial Yes  🗷 No |
|  | Comments: None | | |
| **9. Did the review authors use a satisfactory technique for assessing the risk of bias (RoB) in individual studies that were included in the review?** | | | |
|  | **RCTs**  For Partial Yes, must have assessed RoB from   unconcealed allocation, *and*   lack of blinding of patients and assessors when assessing outcomes (unnecessary for objective outcomes such as all-cause mortality) | For Yes, must also have assessed RoB from:   allocation sequence that was not truly random, *and*   selection of the reported result from among multiple measurements or analyses of a specified outcome |  Yes   Partial Yes   No   Includes only NRSI |
|  | **NRSI**  For Partial Yes, must have assessed RoB:   from confounding, *and*   from selection bias | For Yes, must also have assessed RoB:   methods used to ascertain exposures and outcomes, *and*   selection of the reported result from among multiple measurements or analyses of a specified outcome |  Yes   Partial Yes  🗷 No  Includes only RCTs |
| Comments: None | | | |

| **10. Did the review authors report outcomes on the sources of funding for the studies included in the review?** | | | | | | |
| --- | --- | --- | --- | --- | --- | --- |
| For Yes:   Must have reported on the sources of funding for individual studies included in the review.  Note: Reporting that the reviewers looked for this information but it was not reported by study authors also qualifies. | | | |  Yes  🗷 No |  |  |
| Comments: None | | | |  |  |  |
| **11. If meta-analysis was performed did the review authors use appropriate methods for statistical combination of results?** | | | | | | |
|  | **RCTs**  For Yes:   The authors justified combining the data in a meta-analysis   AND they used an appropriate weighted technique to combine study results and adjusted for heterogeneity if present.   AND investigated the causes of any heterogeneity | |  Yes   No   No meta-analysis conducted | | |  |
|  | **For NRSI**  For Yes:   The authors justified combining the data in a meta-analysis   AND they used an appropriate weighted technique to combine study results, adjusting for heterogeneity if present   AND they statistically combined effect estimates from NRSI that were adjusted for confounding, rather than combining raw data, or justified combining raw data when adjusted effect estimates were not available   AND they reported separate summary estimates for RCTs and NRSI separately when both were included in the review | |  Yes   No  🗷 No meta-analysis conducted | | |  |
|  | Comments: None | | | | |  |
| **12. If meta-analysis was performed, did the review authors assess the potential impact of RoB in individual studies on the results of the meta-analysis or other evidence synthesis?** | | | | | | |
|  | For Yes:   included only low risk of bias RCTs   OR, if the pooled estimate was based on RCTs and/or NRSI at variable RoB, the authors performed analyses to investigate possible impact of RoB on summary estimates of effect. | |  Yes   No  🗷 No meta-analysis conducted | | |  |
|  | Comments: None | | | | |  |
| **13. Did the review authors account for RoB in individual studies when interpreting/ discussing the results of the review?** | | | | | | |
|  | For Yes:   included only low risk of bias RCTs   OR, if RCTs with moderate or high RoB, or NRSI were included the review provided a discussion of the likely impact of RoB on the results | |  Yes  🗷 No | | |  |
|  | Comments: None | | | | |  |
| **14. Did the review authors provide a satisfactory explanation for, and discussion of, any heterogeneity observed in the results of the review?** | | | | | | |
|  | For Yes:   There was no significant heterogeneity in the results   OR if heterogeneity was present the authors performed an investigation of sources of any heterogeneity in the results and discussed the impact of this on the results of the review | |  Yes  🗷 No | | |  |
|  | Comments: None | | | | |  |
| **15. If they performed quantitative synthesis did the review authors carry out an adequate investigation of publication bias (small study bias) and discuss its likely impact on the results of the review?** | | | | | | |
|  | For Yes:   performed graphical or statistical tests for publication bias and discussed the likelihood and magnitude of impact of publication bias | |  Yes   No  🗷 No meta-analysis conducted | | |  |
|  | Comments: None | | | | |  |
| **16. Did the review authors report any potential sources of conflict of interest, including any funding they received for conducting the review?** | | | | | | |
|  | For Yes:   The authors reported no competing interests OR   The authors described their funding sources and how they managed potential conflicts of interest | 🗷 Yes   No | | | |  |
|  | Comments: None | | | | |  |

**To cite this tool:** Shea BJ, Reeves BC, Wells G, Thuku M, Hamel C, Moran J, Moher D, Tugwell P, Welch V, Kristjansson E, Henry DA. AMSTAR 2: a critical appraisal tool for systematic reviews that include randomised or non-randomised studies of healthcare interventions, or both. BMJ. 2017 Sep 21;358:j4008.

# Lupia et al.

| **1. Did the research questions and inclusion criteria for the review include the components of PICO?** | | | | | |
| --- | --- | --- | --- | --- | --- |
| For Yes:   🗷 Population  n/a Intervention  n/a Comparator group  🗷 Outcome | | Optional (recommended)   Timeframe for follow-up | 🗷 | Yes  No |  |
|  Comments: As this was a SR of non-intervention studies, I-intervention and C-comparison were not applicable. | | | | |  |
| **2. Did the report of the review contain an explicit statement that the review methods were established prior to the conduct of the review and did the report justify any significant deviations from the protocol?** | | | | | |
|  | For Partial Yes:  The authors state that they had a written protocol or guide that included ALL the following:   review question(s)   a search strategy   inclusion/exclusion criteria   a risk of bias assessment | For Yes:  As for partial yes, plus the protocol should be registered and should also have specified:   a meta-analysis/synthesis plan, if appropriate, *and*   a plan for investigating causes of heterogeneity   justification for any deviations from the protocol |     🗷 | Yes Partial Yes No |  |
|  | Comments: None | | | |  |
| **3. Did the review authors explain their selection of the study designs for inclusion in the review?** | | | | | |
|  | For Yes, the review should satisfy ONE of the following:   *Explanation for* including only RCTs   OR *Explanation for* including only NRSI   OR *Explanation for* including both RCTs and NRSI | |   🗷 | Yes No |  |
|  | Comments: None | | | |  |
| **4. Did the review authors use a comprehensive literature search strategy?** | | | | | |
|  | For Partial Yes (all the following): | For Yes, should also have (all the following):   searched the reference lists / bibliographies of included studies   searched trial/study registries   included/consulted content experts in the field   where relevant, searched for grey literature   conducted search within 24 months of completion of the review |  |  |  |
|  | 🗷 searched at least 2 databases (relevant to research question)  🗷 provided key word and/or search strategy   justified publication restrictions |  |     🗷 | Yes Partial Yes No |  |
|  | (e.g. language) |  |  |  |  |
|  | Comments: Only English studies included. | | | |  |
|  | **5. Did the review authors perform study selection in duplicate?** | |  | |  |
|  | For Yes, either ONE of the following:   at least two reviewers independently agreed on selection of eligible studies and achieved consensus on which studies to include   OR two reviewers selected a sample of eligible studies and achieved good agreement (at least 80 percent), with the remainder selected by one reviewer. | |   🗷 | Yes No |  |
|  | Comments: Two authors performed study selection but they did not state if it was done independently. | | | |  |

| **6. Did the review authors perform data extraction in duplicate?** | | | |
| --- | --- | --- | --- |
| For Yes, either ONE of the following:   at least two reviewers achieved consensus on which data to extract from included studies   OR two reviewers extracted data from a sample of eligible studies and achieved good agreement (at least 80 percent), with the remainder extracted by one reviewer. | | |  Yes  🗷 No |
| Comments: None | | | |
| **7. Did the review authors provide a list of excluded studies and justify the exclusions?** | | | |
|  | For Partial Yes:   provided a list of all potentially relevant studies that were read in full-text form but excluded from the review | For Yes, must also have:   Justified the exclusion from the review of each potentially relevant study |  Yes   Partial Yes  🗷 No |
|  | Comments: None | | |
| **8. Did the review authors describe the included studies in adequate detail?** | | | |
|  | For Partial Yes (ALL the following):  🗷 described populations  n/a described interventions  n/a described comparators  🗷 described outcomes   described research designs | For Yes, should also have ALL the following:  🗷 described population in detail  n/a described intervention in detail (including doses where relevant)  n/a described comparator in detail (including doses where relevant)  🗷 described study’s setting   timeframe for follow-up |  Yes   Partial Yes  🗷 No |
|  | Comments: None | | |
| **9. Did the review authors use a satisfactory technique for assessing the risk of bias (RoB) in individual studies that were included in the review?** | | | |
|  | **RCTs**  For Partial Yes, must have assessed RoB from   unconcealed allocation, *and*   lack of blinding of patients and assessors when assessing outcomes (unnecessary for objective outcomes such as all-cause mortality) | For Yes, must also have assessed RoB from:   allocation sequence that was not truly random, *and*   selection of the reported result from among multiple measurements or analyses of a specified outcome |  Yes   Partial Yes   No   Includes only NRSI |
|  | **NRSI**  For Partial Yes, must have assessed RoB:   from confounding, *and*   from selection bias | For Yes, must also have assessed RoB:   methods used to ascertain exposures and outcomes, *and*   selection of the reported result from among multiple measurements or analyses of a specified outcome |  Yes   Partial Yes  🗷 No  Includes only RCTs |
| Comments: None | | | |

| **10. Did the review authors report outcomes on the sources of funding for the studies included in the review?** | | | | | | |
| --- | --- | --- | --- | --- | --- | --- |
| For Yes:   Must have reported on the sources of funding for individual studies included in the review.  Note: Reporting that the reviewers looked for this information but it was not reported by study authors also qualifies. | | | |  Yes  🗷 No |  |  |
| Comments: None | | | |  |  |  |
| **11. If meta-analysis was performed did the review authors use appropriate methods for statistical combination of results?** | | | | | | |
|  | **RCTs**  For Yes:   The authors justified combining the data in a meta-analysis   AND they used an appropriate weighted technique to combine study results and adjusted for heterogeneity if present.   AND investigated the causes of any heterogeneity | |  Yes   No   No meta-analysis conducted | | |  |
|  | **For NRSI**  For Yes:   The authors justified combining the data in a meta-analysis   AND they used an appropriate weighted technique to combine study results, adjusting for heterogeneity if present   AND they statistically combined effect estimates from NRSI that were adjusted for confounding, rather than combining raw data, or justified combining raw data when adjusted effect estimates were not available   AND they reported separate summary estimates for RCTs and NRSI separately when both were included in the review | |  Yes   No  🗷 No meta-analysis conducted | | |  |
|  | Comments: None | | | | |  |
| **12. If meta-analysis was performed, did the review authors assess the potential impact of RoB in individual studies on the results of the meta-analysis or other evidence synthesis?** | | | | | | |
|  | For Yes:   included only low risk of bias RCTs   OR, if the pooled estimate was based on RCTs and/or NRSI at variable RoB, the authors performed analyses to investigate possible impact of RoB on summary estimates of effect. | |  Yes   No  🗷 No meta-analysis conducted | | |  |
|  | Comments: None | | | | |  |
| **13. Did the review authors account for RoB in individual studies when interpreting/ discussing the results of the review?** | | | | | | |
|  | For Yes:   included only low risk of bias RCTs   OR, if RCTs with moderate or high RoB, or NRSI were included the review provided a discussion of the likely impact of RoB on the results | |  Yes  🗷 No | | |  |
|  | Comments: None | | | | |  |
| **14. Did the review authors provide a satisfactory explanation for, and discussion of, any heterogeneity observed in the results of the review?** | | | | | | |
|  | For Yes:   There was no significant heterogeneity in the results   OR if heterogeneity was present the authors performed an investigation of sources of any heterogeneity in the results and discussed the impact of this on the results of the review | |  Yes  🗷 No | | |  |
|  | Comments: None | | | | |  |
| **15. If they performed quantitative synthesis did the review authors carry out an adequate investigation of publication bias (small study bias) and discuss its likely impact on the results of the review?** | | | | | | |
|  | For Yes:   performed graphical or statistical tests for publication bias and discussed the likelihood and magnitude of impact of publication bias | |  Yes   No  🗷 No meta-analysis conducted | | |  |
|  | Comments: None | | | | |  |
| **16. Did the review authors report any potential sources of conflict of interest, including any funding they received for conducting the review?** | | | | | | |
|  | For Yes:  🗷 The authors reported no competing interests OR   The authors described their funding sources and how they managed potential conflicts of interest | 🗷 Yes   No | | | |  |
|  | Comments: None | | | | |  |

**To cite this tool:** Shea BJ, Reeves BC, Wells G, Thuku M, Hamel C, Moran J, Moher D, Tugwell P, Welch V, Kristjansson E, Henry DA. AMSTAR 2: a critical appraisal tool for systematic reviews that include randomised or non-randomised studies of healthcare interventions, or both. BMJ. 2017 Sep 21;358:j4008.

# Marasinghe

| **1. Did the research questions and inclusion criteria for the review include the components of PICO?** | | | | | |
| --- | --- | --- | --- | --- | --- |
| For Yes:   🗷 Population  🗷 Intervention  n/a Comparator group  🗷 Outcome | | Optional (recommended)   Timeframe for follow-up | 🗷 | Yes  No |  |
|  Comments: P = non-COVID-19 diagnosed people; Intervention = face masks; C – n/a; O = infection spread | | | | |  |
| **2. Did the report of the review contain an explicit statement that the review methods were established prior to the conduct of the review and did the report justify any significant deviations from the protocol?** | | | | | |
|  | For Partial Yes:  The authors state that they had a written protocol or guide that included ALL the following:   review question(s)   a search strategy   inclusion/exclusion criteria   a risk of bias assessment | For Yes:  As for partial yes, plus the protocol should be registered and should also have specified:   a meta-analysis/synthesis plan, if appropriate, *and*   a plan for investigating causes of heterogeneity   justification for any deviations from the protocol |     🗷 | Yes Partial Yes No |  |
|  | Comments: None | | | |  |
| **3. Did the review authors explain their selection of the study designs for inclusion in the review?** | | | | | |
|  | For Yes, the review should satisfy ONE of the following:   *Explanation for* including only RCTs   OR *Explanation for* including only NRSI   OR *Explanation for* including both RCTs and NRSI | | 🗷 | Yes No |  |
|  | Comments: They included all types of study designs. | | | |  |
| **4. Did the review authors use a comprehensive literature search strategy?** | | | | | |
|  | For Partial Yes (all the following): | For Yes, should also have (all the following):  🗷 searched the reference lists / bibliographies of included studies   searched trial/study registries   included/consulted content experts in the field   where relevant, searched for grey literature  🗷 conducted search within 24 months of completion of the review |  |  |  |
|  | 🗷 searched at least 2 databases (relevant to research question)  🗷 provided key word and/or search strategy   justified publication restrictions |  |     🗷 | Yes Partial Yes No |  |
|  | (e.g. language) |  |  |  |  |
|  | Comments: No justification given for limiting to English, only that it was a limitation. | | | |  |
|  | **5. Did the review authors perform study selection in duplicate?** | |  | |  |
|  | For Yes, either ONE of the following:   at least two reviewers independently agreed on selection of eligible studies and achieved consensus on which studies to include   OR two reviewers selected a sample of eligible studies and achieved good agreement (at least 80 percent), with the remainder selected by one reviewer. | |   🗷 | Yes No |  |
|  | Comments: One author performed study selection. | | | |  |

| **6. Did the review authors perform data extraction in duplicate?** | | | |
| --- | --- | --- | --- |
| For Yes, either ONE of the following:   at least two reviewers achieved consensus on which data to extract from included studies   OR two reviewers extracted data from a sample of eligible studies and achieved good agreement (at least 80 percent), with the remainder extracted by one reviewer. | | |  Yes   No  🗷 not applicable |
| Comments: No studies were identified. | | | |
| **7. Did the review authors provide a list of excluded studies and justify the exclusions?** | | | |
|  | For Partial Yes:   provided a list of all potentially relevant studies that were read in full-text form but excluded from the review | For Yes, must also have:   Justified the exclusion from the review of each potentially relevant study |  Yes   Partial Yes  🗷 No |
|  | Comments: None | | |
| **8. Did the review authors describe the included studies in adequate detail?** | | | |
|  | For Partial Yes (ALL the following):   described populations   described interventions   described comparators   described outcomes   described research designs | For Yes, should also have ALL the following:   described population in detail   described intervention in detail (including doses where relevant)   described comparator in detail (including doses where relevant)   described study’s setting   timeframe for follow-up |  Yes   Partial Yes   No  🗷 not applicable |
|  | Comments: No studies included in the review (empty review) | | |
| **9. Did the review authors use a satisfactory technique for assessing the risk of bias (RoB) in individual studies that were included in the review?** | | | |
|  | **RCTs**  For Partial Yes, must have assessed RoB from   unconcealed allocation, *and*   lack of blinding of patients and assessors when assessing outcomes (unnecessary for objective outcomes such as all-cause mortality) | For Yes, must also have assessed RoB from:   allocation sequence that was not truly random, *and*   selection of the reported result from among multiple measurements or analyses of a specified outcome |  Yes   Partial Yes   No   Includes only NRSI |
|  | **NRSI**  For Partial Yes, must have assessed RoB:   from confounding, *and*   from selection bias | For Yes, must also have assessed RoB:   methods used to ascertain exposures and outcomes, *and*   selection of the reported result from among multiple measurements or analyses of a specified outcome |  Yes   Partial Yes   No  Includes only RCTs  🗷 not applicable |
| Comments: None | | | |

| **10. Did the review authors report outcomes on the sources of funding for the studies included in the review?** | | | | | | |
| --- | --- | --- | --- | --- | --- | --- |
| For Yes:   Must have reported on the sources of funding for individual studies included in the review.  Note: Reporting that the reviewers looked for this information but it was not reported by study authors also qualifies. | | | |  Yes   No  🗷 not applicable |  |  |
| Comments: No studies included in the review (empty review) | | | |  |  |  |
| **11. If meta-analysis was performed did the review authors use appropriate methods for statistical combination of results?** | | | | | | |
|  | **RCTs**  For Yes:   The authors justified combining the data in a meta-analysis   AND they used an appropriate weighted technique to combine study results and adjusted for heterogeneity if present.   AND investigated the causes of any heterogeneity | |  Yes   No   No meta-analysis conducted | | |  |
|  | **For NRSI**  For Yes:   The authors justified combining the data in a meta-analysis   AND they used an appropriate weighted technique to combine study results, adjusting for heterogeneity if present   AND they statistically combined effect estimates from NRSI that were adjusted for confounding, rather than combining raw data, or justified combining raw data when adjusted effect estimates were not available   AND they reported separate summary estimates for RCTs and NRSI separately when both were included in the review | |  Yes   No  🗷 No meta-analysis conducted | | |  |
|  | Comments: No studies included in the review (empty review) | | | | |  |
| **12. If meta-analysis was performed, did the review authors assess the potential impact of RoB in individual studies on the results of the meta-analysis or other evidence synthesis?** | | | | | | |
|  | For Yes:   included only low risk of bias RCTs   OR, if the pooled estimate was based on RCTs and/or NRSI at variable RoB, the authors performed analyses to investigate possible impact of RoB on summary estimates of effect. | |  Yes   No  🗷 No meta-analysis conducted | | |  |
|  | Comments: No studies included in the review (empty review) | | | | |  |
| **13. Did the review authors account for RoB in individual studies when interpreting/ discussing the results of the review?** | | | | | | |
|  | For Yes:   included only low risk of bias RCTs   OR, if RCTs with moderate or high RoB, or NRSI were included the review provided a discussion of the likely impact of RoB on the results | |  Yes   No  🗷 not applicable | | |  |
|  | Comments: No studies included in the review (empty review) | | | | |  |
| **14. Did the review authors provide a satisfactory explanation for, and discussion of, any heterogeneity observed in the results of the review?** | | | | | | |
|  | For Yes:   There was no significant heterogeneity in the results   OR if heterogeneity was present the authors performed an investigation of sources of any heterogeneity in the results and discussed the impact of this on the results of the review | |  Yes   No  🗷 not applicable | | |  |
|  | Comments: No studies included in the review (empty review) | | | | |  |
| **15. If they performed quantitative synthesis did the review authors carry out an adequate investigation of publication bias (small study bias) and discuss its likely impact on the results of the review?** | | | | | | |
|  | For Yes:   performed graphical or statistical tests for publication bias and discussed the likelihood and magnitude of impact of publication bias | |  Yes   No  🗷 No meta-analysis conducted | | |  |
|  | Comments: No studies included in the review (empty review) | | | | |  |
| **16. Did the review authors report any potential sources of conflict of interest, including any funding they received for conducting the review?** | | | | | | |
|  | For Yes:  🗷 The authors reported no competing interests OR   The authors described their funding sources and how they managed potential conflicts of interest | 🗷 Yes   No | | | |  |
|  | Comments: None | | | | |  |

**To cite this tool:** Shea BJ, Reeves BC, Wells G, Thuku M, Hamel C, Moran J, Moher D, Tugwell P, Welch V, Kristjansson E, Henry DA. AMSTAR 2: a critical appraisal tool for systematic reviews that include randomised or non-randomised studies of healthcare interventions, or both. BMJ. 2017 Sep 21;358:j4008.

# Mullins et al.

| **1. Did the research questions and inclusion criteria for the review include the components of PICO?** | | | | | |
| --- | --- | --- | --- | --- | --- |
| For Yes:   🗷 Population  n/a Intervention  n/a Comparator group  🗷 Outcome | | Optional (recommended)   Timeframe for follow-up | 🗷   | Yes  No |  |
|  Comments: Since this SR was of case studies, we considered that I-Intervention and C-Comparator group were not applicable. | | | | |  |
| **2. Did the report of the review contain an explicit statement that the review methods were established prior to the conduct of the review and did the report justify any significant deviations from the protocol?** | | | | | |
|  | For Partial Yes:  The authors state that they had a written protocol or guide that included ALL the following:   review question(s)   a search strategy   inclusion/exclusion criteria   a risk of bias assessment | For Yes:  As for partial yes, plus the protocol should be registered and should also have specified:   a meta-analysis/synthesis plan, if appropriate, *and*   a plan for investigating causes of heterogeneity   justification for any deviations from the protocol |     🗷 | Yes Partial Yes No |  |
|  | Comments: Stated a protocol was not put in PROSPERO due to time restraints. | | | |  |
| **3. Did the review authors explain their selection of the study designs for inclusion in the review?** | | | | | |
|  | For Yes, the review should satisfy ONE of the following:   *Explanation for* including only RCTs   OR *Explanation for* including only NRSI   OR *Explanation for* including both RCTs and NRSI | |   🗷 | Yes No |  |
|  | Comments: They listed the included studies (RCTs and NRSI) but did not give any explanation for this. | | | |  |
| **4. Did the review authors use a comprehensive literature search strategy?** | | | | | |
|  | For Partial Yes (all the following): | For Yes, should also have (all the following):  🗷 searched the reference lists / bibliographies of included studies   searched trial/study registries  🗷 included/consulted content experts in the field   where relevant, searched for grey literature  🗷 conducted search within 24 months of completion of the review |  |  |  |
|  | 🗷 searched at least 2 databases (relevant to research question)   provided key word and/or search strategy  🗷 justified publication restrictions |  |     🗷 | Yes Partial Yes No |  |
|  | (e.g. language) |  |  |  |  |
|  | Comments: No language restrictions. They discussed their topic but did not indicate which terms were keywords or used in the search strategy. Keywords were listed for the article, not the search. | | | |  |
|  | **5. Did the review authors perform study selection in duplicate?** | |  | |  |
|  | For Yes, either ONE of the following:   at least two reviewers independently agreed on selection of eligible studies and achieved consensus on which studies to include   OR two reviewers selected a sample of eligible studies and achieved good agreement (at least 80 percent), with the remainder selected by one reviewer. | |   🗷 | Yes No |  |
|  | Comments: None | | | |  |

| **6. Did the review authors perform data extraction in duplicate?** | | | |
| --- | --- | --- | --- |
| For Yes, either ONE of the following:   at least two reviewers achieved consensus on which data to extract from included studies   OR two reviewers extracted data from a sample of eligible studies and achieved good agreement (at least 80 percent), with the remainder extracted by one reviewer. | | |  Yes  🗷 No |
| Comments: None | | | |
| **7. Did the review authors provide a list of excluded studies and justify the exclusions?** | | | |
|  | For Partial Yes:   provided a list of all potentially relevant studies that were read in full-text form but excluded from the review | For Yes, must also have:   Justified the exclusion from the review of each potentially relevant study | 🗷 Yes   Partial Yes   No |
|  | Comments: The authors reported: “The search of PubMed identified 9965 results; 69 abstracts were screened, of which 48 were excluded due to the study not including pregnant women or humans, or being an in-vitro study. Twenty-one relevant studies were identified^2–22^; their full texts were reviewed and all 21 were included.” Thus, the authors did not have studies that were read in full-text and then excluded. We scored this item “Yes”, as we did not have a reason to penalize the authors for this item. | | |
| **8. Did the review authors describe the included studies in adequate detail?** | | | |
|  | For Partial Yes (ALL the following):  🗷 described populations  n/a described interventions  n/a described comparators  🗷 described outcomes   described research designs | For Yes, should also have ALL the following:   described population in detail   described intervention in detail (including doses where relevant)   described comparator in detail (including doses where relevant)   described study’s setting   timeframe for follow-up |  Yes   Partial Yes  🗷 No |
|  | Comments: None | | |
| **9. Did the review authors use a satisfactory technique for assessing the risk of bias (RoB) in individual studies that were included in the review?** | | | |
|  | **RCTs**  For Partial Yes, must have assessed RoB from   unconcealed allocation, *and*   lack of blinding of patients and assessors when assessing outcomes (unnecessary for objective outcomes such as all-cause mortality) | For Yes, must also have assessed RoB from:   allocation sequence that was not truly random, *and*   selection of the reported result from among multiple measurements or analyses of a specified outcome |  Yes   Partial Yes   No   Includes only NRSI |
|  | **NRSI**  For Partial Yes, must have assessed RoB:   from confounding, *and*   from selection bias | For Yes, must also have assessed RoB:   methods used to ascertain exposures and outcomes, *and*   selection of the reported result from among multiple measurements or analyses of a specified outcome |  Yes   Partial Yes  🗷 No  Includes only RCTs |
| Comments: None | | | |

| **10. Did the review authors report outcomes on the sources of funding for the studies included in the review?** | | | | | | |
| --- | --- | --- | --- | --- | --- | --- |
| For Yes:   Must have reported on the sources of funding for individual studies included in the review.  Note: Reporting that the reviewers looked for this information but it was not reported by study authors also qualifies. | | | |  Yes  🗷 No |  |  |
| Comments: None | | | |  |  |  |
| **11. If meta-analysis was performed did the review authors use appropriate methods for statistical combination of results?** | | | | | | |
|  | **RCTs**  For Yes:   The authors justified combining the data in a meta-analysis   AND they used an appropriate weighted technique to combine study results and adjusted for heterogeneity if present.   AND investigated the causes of any heterogeneity | |  Yes   No   No meta-analysis conducted | | |  |
|  | **For NRSI**  For Yes:   The authors justified combining the data in a meta-analysis   AND they used an appropriate weighted technique to combine study results, adjusting for heterogeneity if present   AND they statistically combined effect estimates from NRSI that were adjusted for confounding, rather than combining raw data, or justified combining raw data when adjusted effect estimates were not available   AND they reported separate summary estimates for RCTs and NRSI separately when both were included in the review | |  Yes   No  🗷 No meta-analysis conducted | | |  |
|  | Comments: None | | | | |  |
| **12. If meta-analysis was performed, did the review authors assess the potential impact of RoB in individual studies on the results of the meta-analysis or other evidence synthesis?** | | | | | | |
|  | For Yes:   included only low risk of bias RCTs   OR, if the pooled estimate was based on RCTs and/or NRSI at variable RoB, the authors performed analyses to investigate possible impact of RoB on summary estimates of effect. | |  Yes   No  🗷 No meta-analysis conducted | | |  |
|  | Comments: None | | | | |  |
| **13. Did the review authors account for RoB in individual studies when interpreting/ discussing the results of the review?** | | | | | | |
|  | For Yes:   included only low risk of bias RCTs   OR, if RCTs with moderate or high RoB, or NRSI were included the review provided a discussion of the likely impact of RoB on the results | |  Yes  🗷 No | | |  |
|  | Comments: None | | | | |  |
| **14. Did the review authors provide a satisfactory explanation for, and discussion of, any heterogeneity observed in the results of the review?** | | | | | | |
|  | For Yes:   There was no significant heterogeneity in the results   OR if heterogeneity was present the authors performed an investigation of sources of any heterogeneity in the results and discussed the impact of this on the results of the review | |  Yes  🗷 No | | |  |
|  | Comments: None | | | | |  |
| **15. If they performed quantitative synthesis did the review authors carry out an adequate investigation of publication bias (small study bias) and discuss its likely impact on the results of the review?** | | | | | | |
|  | For Yes:   performed graphical or statistical tests for publication bias and discussed the likelihood and magnitude of impact of publication bias | |  Yes   No  🗷 No meta-analysis conducted | | |  |
|  | Comments: None | | | | |  |
| **16. Did the review authors report any potential sources of conflict of interest, including any funding they received for conducting the review?** | | | | | | |
|  | For Yes:  🗷 The authors reported no competing interests OR  🗷 The authors described their funding sources and how they managed potential conflicts of interest | 🗷 Yes   No | | | |  |
|  | Comments: None | | | | |  |

**To cite this tool:** Shea BJ, Reeves BC, Wells G, Thuku M, Hamel C, Moran J, Moher D, Tugwell P, Welch V, Kristjansson E, Henry DA. AMSTAR 2: a critical appraisal tool for systematic reviews that include randomised or non-randomised studies of healthcare interventions, or both. BMJ. 2017 Sep 21;358:j4008.

# Pang et al.

| **1. Did the research questions and inclusion criteria for the review include the components of PICO?** | | | | | |
| --- | --- | --- | --- | --- | --- |
| For Yes:   🗷 Population  🗷 Intervention   Comparator group  🗷 Outcome | | Optional (recommended)   Timeframe for follow-up |   🗷 | Yes  No |  |
|  Comments: Interventions were vaccines or therapeutics, plus other types of SR. | | | | |  |
| **2. Did the report of the review contain an explicit statement that the review methods were established prior to the conduct of the review and did the report justify any significant deviations from the protocol?** | | | | | |
|  | For Partial Yes:  The authors state that they had a written protocol or guide that included ALL the following:   review question(s)   a search strategy   inclusion/exclusion criteria   a risk of bias assessment | For Yes:  As for partial yes, plus the protocol should be registered and should also have specified:   a meta-analysis/synthesis plan, if appropriate, *and*   a plan for investigating causes of heterogeneity   justification for any deviations from the protocol |     🗷 | Yes Partial Yes No |  |
|  | Comments: None | | | |  |
| **3. Did the review authors explain their selection of the study designs for inclusion in the review?** | | | | | |
|  | For Yes, the review should satisfy ONE of the following:   *Explanation for* including only RCTs   OR *Explanation for* including only NRSI   OR *Explanation for* including both RCTs and NRSI | | 🗷   | Yes No |  |
|  | Comments: All designs included. | | | |  |
| **4. Did the review authors use a comprehensive literature search strategy?** | | | | | |
|  | For Partial Yes (all the following): | For Yes, should also have (all the following):  🗷 searched the reference lists / bibliographies of included studies  🗷 searched trial/study registries  🗷 included/consulted content experts in the field  🗷 where relevant, searched for grey literature  🗷 conducted search within 24 months of completion of the review |  |  |  |
|  | 🗷 searched at least 2 databases (relevant to research question)  🗷 provided key word and/or search strategy   justified publication restrictions |  |     🗷 | Yes Partial Yes No |  |
|  | (e.g. language) |  |  |  |  |
|  | Comments: English restriction not justified. | | | |  |
|  | **5. Did the review authors perform study selection in duplicate?** | |  | |  |
|  | For Yes, either ONE of the following:   at least two reviewers independently agreed on selection of eligible studies and achieved consensus on which studies to include   OR two reviewers selected a sample of eligible studies and achieved good agreement (at least 80 percent), with the remainder selected by one reviewer. | | 🗷 | Yes No |  |
|  | Comments: Two independent reviewers used (p. 2). | | | |  |

| **6. Did the review authors perform data extraction in duplicate?** | | | |
| --- | --- | --- | --- |
| For Yes, either ONE of the following:   at least two reviewers achieved consensus on which data to extract from included studies   OR two reviewers extracted data from a sample of eligible studies and achieved good agreement (at least 80 percent), with the remainder extracted by one reviewer. | | |  Yes  🗷 No |
| Comments: None | | | |
| **7. Did the review authors provide a list of excluded studies and justify the exclusions?** | | | |
|  | For Partial Yes:   provided a list of all potentially relevant studies that were read in full-text form but excluded from the review | For Yes, must also have:   Justified the exclusion from the review of each potentially relevant study |  Yes   Partial Yes  🗷 No |
|  | Comments: They gave the numbers of studies excluded and the reasons for exclusion, but not a list. | | |
| **8. Did the review authors describe the included studies in adequate detail?** | | | |
|  | For Partial Yes (ALL the following):  🗷 described populations  🗷 described interventions  n/a described comparators  🗷 described outcomes  🗷 described research designs | For Yes, should also have ALL the following:  🗷 described population in detail  🗷 described intervention in detail (including doses where relevant)  🗷 described comparator in detail (including doses where relevant)  🗷 described study’s setting  🗷 timeframe for follow-up | 🗷 Yes   Partial Yes   No |
|  | Comments: None | | |
| **9. Did the review authors use a satisfactory technique for assessing the risk of bias (RoB) in individual studies that were included in the review?** | | | |
|  | **RCTs**  For Partial Yes, must have assessed RoB from   unconcealed allocation, *and*   lack of blinding of patients and assessors when assessing outcomes (unnecessary for objective outcomes such as all-cause mortality) | For Yes, must also have assessed RoB from:   allocation sequence that was not truly random, *and*   selection of the reported result from among multiple measurements or analyses of a specified outcome |  Yes   Partial Yes  🗷 No   Includes only NRSI |
|  | **NRSI**  For Partial Yes, must have assessed RoB:   from confounding, *and*   from selection bias | For Yes, must also have assessed RoB:   methods used to ascertain exposures and outcomes, *and*   selection of the reported result from among multiple measurements or analyses of a specified outcome |  Yes   Partial Yes  🗷 No  Includes only RCTs |
| Comments: None | | | |

| **10. Did the review authors report outcomes on the sources of funding for the studies included in the review?** | | | | | | |
| --- | --- | --- | --- | --- | --- | --- |
| For Yes:   Must have reported on the sources of funding for individual studies included in the review.  Note: Reporting that the reviewers looked for this information but it was not reported by study authors also qualifies. | | | |  Yes  🗷 No |  |  |
| Comments: Funding of vaccines was reported, but not the other interventions. However, since this item does not have an option of a “Partial Yes”, we scored “No”. | | | |  |  |  |
| **11. If meta-analysis was performed did the review authors use appropriate methods for statistical combination of results?** | | | | | | |
|  | **RCTs**  For Yes:   The authors justified combining the data in a meta-analysis   AND they used an appropriate weighted technique to combine study results and adjusted for heterogeneity if present.   AND investigated the causes of any heterogeneity | |  Yes   No  🗷 No meta-analysis conducted | | |  |
|  | **For NRSI**  For Yes:   The authors justified combining the data in a meta-analysis   AND they used an appropriate weighted technique to combine study results, adjusting for heterogeneity if present   AND they statistically combined effect estimates from NRSI that were adjusted for confounding, rather than combining raw data, or justified combining raw data when adjusted effect estimates were not available   AND they reported separate summary estimates for RCTs and NRSI separately when both were included in the review | |  Yes   No  🗷 No meta-analysis conducted | | |  |
|  | Comments: None | | | | |  |
| **12. If meta-analysis was performed, did the review authors assess the potential impact of RoB in individual studies on the results of the meta-analysis or other evidence synthesis?** | | | | | | |
|  | For Yes:   included only low risk of bias RCTs   OR, if the pooled estimate was based on RCTs and/or NRSI at variable RoB, the authors performed analyses to investigate possible impact of RoB on summary estimates of effect. | |  Yes   No  🗷 No meta-analysis conducted | | |  |
|  | Comments: None | | | | |  |
| **13. Did the review authors account for RoB in individual studies when interpreting/ discussing the results of the review?** | | | | | | |
|  | For Yes:   included only low risk of bias RCTs   OR, if RCTs with moderate or high RoB, or NRSI were included the review provided a discussion of the likely impact of RoB on the results | |  Yes  🗷 No | | |  |
|  | Comments: None | | | | |  |
| **14. Did the review authors provide a satisfactory explanation for, and discussion of, any heterogeneity observed in the results of the review?** | | | | | | |
|  | For Yes:   There was no significant heterogeneity in the results   OR if heterogeneity was present the authors performed an investigation of sources of any heterogeneity in the results and discussed the impact of this on the results of the review | |  Yes  🗷 No | | |  |
|  | Comments: None | | | | |  |
| **15. If they performed quantitative synthesis did the review authors carry out an adequate investigation of publication bias (small study bias) and discuss its likely impact on the results of the review?** | | | | | | |
|  | For Yes:   performed graphical or statistical tests for publication bias and discussed the likelihood and magnitude of impact of publication bias | |  Yes   No  🗷 No meta-analysis conducted | | |  |
|  | Comments: None | | | | |  |
| **16. Did the review authors report any potential sources of conflict of interest, including any funding they received for conducting the review?** | | | | | | |
|  | For Yes:   The authors reported no competing interests OR  🗷 The authors described their funding sources and how they managed potential conflicts of interest | 🗷 Yes   No | | | |  |
|  | Comments: None | | | | |  |

**To cite this tool:** Shea BJ, Reeves BC, Wells G, Thuku M, Hamel C, Moran J, Moher D, Tugwell P, Welch V, Kristjansson E, Henry DA. AMSTAR 2: a critical appraisal tool for systematic reviews that include randomised or non-randomised studies of healthcare interventions, or both. BMJ. 2017 Sep 21;358:j4008.

# Rodriguez-Morales et al.

| **1. Did the research questions and inclusion criteria for the review include the components of PICO?** | | | | | |
| --- | --- | --- | --- | --- | --- |
| For Yes:   🗷 Population  n/a Intervention  n/a Comparator group  🗷 Outcome | | Optional (recommended)   Timeframe for follow-up | 🗷 | Yes  No |  |
|  Comments: Since this SR was of case studies, we considered that I-Intervention and C-Comparator group were not applicable. | | | | |  |
| **2. Did the report of the review contain an explicit statement that the review methods were established prior to the conduct of the review and did the report justify any significant deviations from the protocol?** | | | | | |
|  | For Partial Yes:  The authors state that they had a written protocol or guide that included ALL the following:   review question(s)   a search strategy   inclusion/exclusion criteria   a risk of bias assessment | For Yes:  As for partial yes, plus the protocol should be registered and should also have specified:   a meta-analysis/synthesis plan, if appropriate, *and*   a plan for investigating causes of heterogeneity   justification for any deviations from the protocol | 🗷     | Yes Partial Yes No |  |
|  | Comments: Stated on p. 2. Protocol in PROSPERO provides all details. | | | |  |
| **3. Did the review authors explain their selection of the study designs for inclusion in the review?** | | | | | |
|  | For Yes, the review should satisfy ONE of the following:   *Explanation for* including only RCTs  🗷 OR *Explanation for* including only NRSI   OR *Explanation for* including both RCTs and NRSI | | 🗷 | Yes No |  |
|  | Comments: They explained why case studies and NRSI studies were selected. | | | |  |
| **4. Did the review authors use a comprehensive literature search strategy?** | | | | | |
|  | For Partial Yes (all the following): | For Yes, should also have (all the following):   searched the reference lists / bibliographies of included studies   searched trial/study registries  🗷 included/consulted content experts in the field   where relevant, searched for grey literature  🗷 conducted search within 24 months of completion of the review |  |  |  |
|  | 🗷 searched at least 2 databases (relevant to research question)  🗷 provided key word and/or search strategy  🗷 justified publication restrictions |  |   🗷 | Yes Partial Yes No |  |
|  | (e.g. language) |  |  |  |  |
|  | Comments: No language restrictions. | | | |  |
|  | **5. Did the review authors perform study selection in duplicate?** | |  | |  |
|  | For Yes, either ONE of the following:  🗷 at least two reviewers independently agreed on selection of eligible studies and achieved consensus on which studies to include   OR two reviewers selected a sample of eligible studies and achieved good agreement (at least 80 percent), with the remainder selected by one reviewer. | | 🗷 | Yes No |  |
|  | Comments: None | | | |  |

| **6. Did the review authors perform data extraction in duplicate?** | | | |
| --- | --- | --- | --- |
| For Yes, either ONE of the following:  🗷 at least two reviewers achieved consensus on which data to extract from included studies   OR two reviewers extracted data from a sample of eligible studies and achieved good agreement (at least 80 percent), with the remainder extracted by one reviewer. | | | 🗷 Yes   No |
| Comments: None | | | |
| **7. Did the review authors provide a list of excluded studies and justify the exclusions?** | | | |
|  | For Partial Yes:   provided a list of all potentially relevant studies that were read in full-text form but excluded from the review | For Yes, must also have:   Justified the exclusion from the review of each potentially relevant study |  Yes   Partial Yes  🗷 No |
|  | Comments: They reported the numbers of studies excluded and the reasons for exclusion, but not the list of the studies | | |
| **8. Did the review authors describe the included studies in adequate detail?** | | | |
|  | For Partial Yes (ALL the following):  🗷 described populations  n/a described interventions  n/a described comparators  🗷 described outcomes  🗷 described research designs | For Yes, should also have ALL the following:  🗷 described population in detail  🗷 described intervention in detail (including doses where relevant)   described comparator in detail (including doses where relevant)  🗷 described study’s setting   timeframe for follow-up |  Yes  🗷 Partial Yes   No |
|  | Comments: They gave the research designs, but not in detail such as how cases were selected or observational studies conducted. | | |
| **9. Did the review authors use a satisfactory technique for assessing the risk of bias (RoB) in individual studies that were included in the review?** | | | |
|  | **RCTs**  For Partial Yes, must have assessed RoB from   unconcealed allocation, *and*   lack of blinding of patients and assessors when assessing outcomes (unnecessary for objective outcomes such as all-cause mortality) | For Yes, must also have assessed RoB from:   allocation sequence that was not truly random, *and*   selection of the reported result from among multiple measurements or analyses of a specified outcome |  Yes   Partial Yes   No   Includes only NRSI |
|  | **NRSI**  For Partial Yes, must have assessed RoB:   from confounding, *and*   from selection bias | For Yes, must also have assessed RoB:   methods used to ascertain exposures and outcomes, *and*   selection of the reported result from among multiple measurements or analyses of a specified outcome |  Yes   Partial Yes  🗷 No  Includes only RCTs |
| Comments: They reported that they used the IHE Quality Appraisal of Case Series Studies checklist and AXIS for cross-sectional studies. However, no results were presented. | | | |

| **10. Did the review authors report outcomes on the sources of funding for the studies included in the review?** | | | | | | |
| --- | --- | --- | --- | --- | --- | --- |
| For Yes:   Must have reported on the sources of funding for individual studies included in the review.  Note: Reporting that the reviewers looked for this information but it was not reported by study authors also qualifies. | | | |  Yes  🗷 No |  |  |
| Comments: None | | | |  |  |  |
| **11. If meta-analysis was performed did the review authors use appropriate methods for statistical combination of results?** | | | | | | |
|  | **RCTs**  For Yes:   The authors justified combining the data in a meta-analysis   AND they used an appropriate weighted technique to combine study results and adjusted for heterogeneity if present.   AND investigated the causes of any heterogeneity | |  Yes   No   No meta-analysis conducted | | |  |
|  | **For NRSI**  For Yes:  🗷 The authors justified combining the data in a meta-analysis  🗷 AND they used an appropriate weighted technique to combine study results, adjusting for heterogeneity if present  🗷 AND they statistically combined effect estimates from NRSI that were adjusted for confounding, rather than combining raw data, or justified combining raw data when adjusted effect estimates were not available   AND they reported separate summary estimates for RCTs and NRSI separately when both were included in the review | | 🗷 Yes   No   No meta-analysis conducted | | |  |
|  | Comments: None | | | | |  |
| **12. If meta-analysis was performed, did the review authors assess the potential impact of RoB in individual studies on the results of the meta-analysis or other evidence synthesis?** | | | | | | |
|  | For Yes:   included only low risk of bias RCTs   OR, if the pooled estimate was based on RCTs and/or NRSI at variable RoB, the authors performed analyses to investigate possible impact of RoB on summary estimates of effect. | |  Yes  🗷 No   No meta-analysis conducted | | |  |
|  | Comments: No discussion of impact of RoB. | | | | |  |
| **13. Did the review authors account for RoB in individual studies when interpreting/ discussing the results of the review?** | | | | | | |
|  | For Yes:   included only low risk of bias RCTs   OR, if RCTs with moderate or high RoB, or NRSI were included the review provided a discussion of the likely impact of RoB on the results | |  Yes  🗷 No | | |  |
|  | Comments: None | | | | |  |
| **14. Did the review authors provide a satisfactory explanation for, and discussion of, any heterogeneity observed in the results of the review?** | | | | | | |
|  | For Yes:   There was no significant heterogeneity in the results   OR if heterogeneity was present the authors performed an investigation of sources of any heterogeneity in the results and discussed the impact of this on the results of the review | |  Yes  🗷 No | | |  |
|  | Comments: Significant heterogeneity was present (Table 6) but not discussed. | | | | |  |
| **15. If they performed quantitative synthesis did the review authors carry out an adequate investigation of publication bias (small study bias) and discuss its likely impact on the results of the review?** | | | | | | |
|  | For Yes:  🗷 performed graphical or statistical tests for publication bias and discussed the likelihood and magnitude of impact of publication bias | | 🗷 Yes   No   No meta-analysis conducted | | |  |
|  | Comments: None | | | | |  |
| **16. Did the review authors report any potential sources of conflict of interest, including any funding they received for conducting the review?** | | | | | | |
|  | For Yes:   The authors reported no competing interests OR  🗷 The authors described their funding sources and how they managed potential conflicts of interest | 🗷 Yes   No | | | |  |
|  | Comments: None | | | | |  |

**To cite this tool:** Shea BJ, Reeves BC, Wells G, Thuku M, Hamel C, Moran J, Moher D, Tugwell P, Welch V, Kristjansson E, Henry DA. AMSTAR 2: a critical appraisal tool for systematic reviews that include randomised or non-randomised studies of healthcare interventions, or both. BMJ. 2017 Sep 21;358:j4008.

# Salehi et al.

| **1. Did the research questions and inclusion criteria for the review include the components of PICO?** | | | | | |
| --- | --- | --- | --- | --- | --- |
| For Yes:   🗷 Population  n/a Intervention  n/a Comparator group  🗷 Outcome | | Optional (recommended)   Timeframe for follow-up | 🗷   | Yes  No |  |
|  Comments: As this was a SR of non-intervention studies, I-intervention and C-comparison were not applicable. | | | | |  |
| **2. Did the report of the review contain an explicit statement that the review methods were established prior to the conduct of the review and did the report justify any significant deviations from the protocol?** | | | | | |
|  | For Partial Yes:  The authors state that they had a written protocol or guide that included ALL the following:   review question(s)   a search strategy   inclusion/exclusion criteria   a risk of bias assessment | For Yes:  As for partial yes, plus the protocol should be registered and should also have specified:   a meta-analysis/synthesis plan, if appropriate, *and*   a plan for investigating causes of heterogeneity   justification for any deviations from the protocol |     🗷 | Yes Partial Yes No |  |
|  | Comments: None | | | |  |
| **3. Did the review authors explain their selection of the study designs for inclusion in the review?** | | | | | |
|  | For Yes, the review should satisfy ONE of the following:   *Explanation for* including only RCTs   OR *Explanation for* including only NRSI   OR *Explanation for* including both RCTs and NRSI | |   🗷 | Yes No |  |
|  | Comments: None | | | |  |
| **4. Did the review authors use a comprehensive literature search strategy?** | | | | | |
|  | For Partial Yes (all the following): | For Yes, should also have (all the following):  🗷 searched the reference lists / bibliographies of included studies   searched trial/study registries  🗷 included/consulted content experts in the field  🗷 where relevant, searched for grey literature  🗷 conducted search within 24 months of completion of the review |  |  |  |
|  | 🗷 searched at least 2 databases (relevant to research question)  🗷 provided key word and/or search strategy   justified publication restrictions |  |    🗷 | Yes Partial Yes No |  |
|  | (e.g. language) |  |  |  |  |
|  | Comments: Restrictions to English were noted as a limitation but jot justified. | | | |  |
|  | **5. Did the review authors perform study selection in duplicate?** | |  | |  |
|  | For Yes, either ONE of the following:  🗷 at least two reviewers independently agreed on selection of eligible studies and achieved consensus on which studies to include   OR two reviewers selected a sample of eligible studies and achieved good agreement (at least 80 percent), with the remainder selected by one reviewer. | | 🗷 | Yes No |  |
|  | Comments: None | | | |  |

| **6. Did the review authors perform data extraction in duplicate?** | | | |
| --- | --- | --- | --- |
| For Yes, either ONE of the following:  🗷 at least two reviewers achieved consensus on which data to extract from included studies   OR two reviewers extracted data from a sample of eligible studies and achieved good agreement (at least 80 percent), with the remainder extracted by one reviewer. | | | 🗷 Yes   No |
| Comments: None | | | |
| **7. Did the review authors provide a list of excluded studies and justify the exclusions?** | | | |
|  | For Partial Yes:   provided a list of all potentially relevant studies that were read in full-text form but excluded from the review | For Yes, must also have:   Justified the exclusion from the review of each potentially relevant study |  Yes   Partial Yes  🗷 No |
|  | Comments: None | | |
| **8. Did the review authors describe the included studies in adequate detail?** | | | |
|  | For Partial Yes (ALL the following):   described populations  n/a described interventions  n/a described comparators  🗷 described outcomes   described research designs | For Yes, should also have ALL the following:   described population in detail  n/a described intervention in detail (including doses where relevant)  n/a described comparator in detail (including doses where relevant)   described study’s setting   timeframe for follow-up |  Yes   Partial Yes  🗷 No |
|  | Comments: None | | |
| **9. Did the review authors use a satisfactory technique for assessing the risk of bias (RoB) in individual studies that were included in the review?** | | | |
|  | **RCTs**  For Partial Yes, must have assessed RoB from   unconcealed allocation, *and*   lack of blinding of patients and assessors when assessing outcomes (unnecessary for objective outcomes such as all-cause mortality) | For Yes, must also have assessed RoB from:   allocation sequence that was not truly random, *and*   selection of the reported result from among multiple measurements or analyses of a specified outcome |  Yes   Partial Yes   No   Includes only NRSI |
|  | **NRSI**  For Partial Yes, must have assessed RoB:  🗷 from confounding, *and*  🗷 from selection bias | For Yes, must also have assessed RoB:  🗷 methods used to ascertain exposures and outcomes, *and*  🗷 selection of the reported result from among multiple measurements or analyses of a specified outcome | 🗷 Yes   Partial Yes   No  Includes only RCTs |
| Comments: They used the National Institutes of Health Quality Assessment Tool for Case Series Studies. This has signaling questions for all these issues. | | | |

| **10. Did the review authors report outcomes on the sources of funding for the studies included in the review?** | | | | | | |
| --- | --- | --- | --- | --- | --- | --- |
| For Yes:   Must have reported on the sources of funding for individual studies included in the review.  Note: Reporting that the reviewers looked for this information but it was not reported by study authors also qualifies. | | | |  Yes  🗷 No |  |  |
| Comments: None | | | |  |  |  |
| **11. If meta-analysis was performed did the review authors use appropriate methods for statistical combination of results?** | | | | | | |
|  | **RCTs**  For Yes:   The authors justified combining the data in a meta-analysis   AND they used an appropriate weighted technique to combine study results and adjusted for heterogeneity if present.   AND investigated the causes of any heterogeneity | |  Yes   No   No meta-analysis conducted | | |  |
|  | **For NRSI**  For Yes:   The authors justified combining the data in a meta-analysis   AND they used an appropriate weighted technique to combine study results, adjusting for heterogeneity if present   AND they statistically combined effect estimates from NRSI that were adjusted for confounding, rather than combining raw data, or justified combining raw data when adjusted effect estimates were not available   AND they reported separate summary estimates for RCTs and NRSI separately when both were included in the review | |  Yes   No  🗷 No meta-analysis conducted | | |  |
|  | Comments: None | | | | |  |
| **12. If meta-analysis was performed, did the review authors assess the potential impact of RoB in individual studies on the results of the meta-analysis or other evidence synthesis?** | | | | | | |
|  | For Yes:   included only low risk of bias RCTs   OR, if the pooled estimate was based on RCTs and/or NRSI at variable RoB, the authors performed analyses to investigate possible impact of RoB on summary estimates of effect. | |  Yes   No  🗷 No meta-analysis conducted | | |  |
|  | Comments: None | | | | |  |
| **13. Did the review authors account for RoB in individual studies when interpreting/ discussing the results of the review?** | | | | | | |
|  | For Yes:   included only low risk of bias RCTs  🗷 OR, if RCTs with moderate or high RoB, or NRSI were included the review provided a discussion of the likely impact of RoB on the results | | 🗷 Yes   No | | |  |
|  | Comments: None | | | | |  |
| **14. Did the review authors provide a satisfactory explanation for, and discussion of, any heterogeneity observed in the results of the review?** | | | | | | |
|  | For Yes:   There was no significant heterogeneity in the results   OR if heterogeneity was present the authors performed an investigation of sources of any heterogeneity in the results and discussed the impact of this on the results of the review | |  Yes  🗷 No | | |  |
|  | Comments: None | | | | |  |
| **15. If they performed quantitative synthesis did the review authors carry out an adequate investigation of publication bias (small study bias) and discuss its likely impact on the results of the review?** | | | | | | |
|  | For Yes:   performed graphical or statistical tests for publication bias and discussed the likelihood and magnitude of impact of publication bias | |  Yes   No  🗷 No meta-analysis conducted | | |  |
|  | Comments: None | | | | |  |
| **16. Did the review authors report any potential sources of conflict of interest, including any funding they received for conducting the review?** | | | | | | |
|  | For Yes:   The authors reported no competing interests OR   The authors described their funding sources and how they managed potential conflicts of interest |  Yes  🗷 No | | | |  |
|  | Comments: None | | | | |  |

**To cite this tool:** Shea BJ, Reeves BC, Wells G, Thuku M, Hamel C, Moran J, Moher D, Tugwell P, Welch V, Kristjansson E, Henry DA. AMSTAR 2: a critical appraisal tool for systematic reviews that include randomised or non-randomised studies of healthcare interventions, or both. BMJ. 2017 Sep 21;358:j4008.

# Sun et al.

| **1. Did the research questions and inclusion criteria for the review include the components of PICO?** | | | | | |
| --- | --- | --- | --- | --- | --- |
| For Yes:   🗷 Population  n/a Intervention  n/a Comparator group  🗷 Outcome | | Optional (recommended)   Timeframe for follow-up | 🗷 | Yes  No |  |
|  Comments: Since this was not a review of studies of interventions, we considered I-intervention and C-Comparator group as not applicable. | | | | |  |
| **2. Did the report of the review contain an explicit statement that the review methods were established prior to the conduct of the review and did the report justify any significant deviations from the protocol?** | | | | | |
|  | For Partial Yes:  The authors state that they had a written protocol or guide that included ALL the following:   review question(s)   a search strategy   inclusion/exclusion criteria   a risk of bias assessment | For Yes:  As for partial yes, plus the protocol should be registered and should also have specified:   a meta-analysis/synthesis plan, if appropriate, *and*   a plan for investigating causes of heterogeneity   justification for any deviations from the protocol |     🗷 | Yes Partial Yes No |  |
|  | Comments: None | | | |  |
| **3. Did the review authors explain their selection of the study designs for inclusion in the review?** | | | | | |
|  | For Yes, the review should satisfy ONE of the following:   *Explanation for* including only RCTs   OR *Explanation for* including only NRSI   OR *Explanation for* including both RCTs and NRSI | | 🗷 | Yes No |  |
|  | Comments: They included all study designs. | | | |  |
| **4. Did the review authors use a comprehensive literature search strategy?** | | | | | |
|  | For Partial Yes (all the following): | For Yes, should also have (all the following):   searched the reference lists / bibliographies of included studies   searched trial/study registries  🗷 included/consulted content experts in the field   where relevant, searched for grey literature  🗷 conducted search within 24 months of completion of the review |  |  |  |
|  | 🗷 searched at least 2 databases (relevant to research question)  🗷 provided key word and/or search strategy  🗷 justified publication restrictions |  |   🗷 | Yes Partial Yes No |  |
|  | (e.g. language) |  |  |  |  |
|  | Comments: No language restrictions. | | | |  |
|  | **5. Did the review authors perform study selection in duplicate?** | |  | |  |
|  | For Yes, either ONE of the following:   at least two reviewers independently agreed on selection of eligible studies and achieved consensus on which studies to include   OR two reviewers selected a sample of eligible studies and achieved good agreement (at least 80 percent), with the remainder selected by one reviewer. | |   🗷 | Yes No |  |
|  | Comments: None | | | |  |

| **6. Did the review authors perform data extraction in duplicate?** | | | |
| --- | --- | --- | --- |
| For Yes, either ONE of the following:   at least two reviewers achieved consensus on which data to extract from included studies   OR two reviewers extracted data from a sample of eligible studies and achieved good agreement (at least 80 percent), with the remainder extracted by one reviewer. | | |  Yes  🗷 No |
| Comments: None | | | |
| **7. Did the review authors provide a list of excluded studies and justify the exclusions?** | | | |
|  | For Partial Yes:   provided a list of all potentially relevant studies that were read in full-text form but excluded from the review | For Yes, must also have:   Justified the exclusion from the review of each potentially relevant study |  Yes   Partial Yes  🗷 No |
|  | Comments: The authors reported the numbers of studies excluded and the reasons for exclusion, but not the list of studies | | |
| **8. Did the review authors describe the included studies in adequate detail?** | | | |
|  | For Partial Yes (ALL the following):  🗷 described populations  n/a described interventions  n/a described comparators  🗷 described outcomes   described research designs | For Yes, should also have ALL the following:  🗷 described population in detail   described intervention in detail (including doses where relevant)   described comparator in detail (including doses where relevant)  🗷 described study’s setting  🗷 timeframe for follow-up |  Yes   Partial Yes  🗷 No |
|  | Comments: Research design just stated as retrospective study with no details. | | |
| **9. Did the review authors use a satisfactory technique for assessing the risk of bias (RoB) in individual studies that were included in the review?** | | | |
|  | **RCTs**  For Partial Yes, must have assessed RoB from   unconcealed allocation, *and*   lack of blinding of patients and assessors when assessing outcomes (unnecessary for objective outcomes such as all-cause mortality) | For Yes, must also have assessed RoB from:   allocation sequence that was not truly random, *and*   selection of the reported result from among multiple measurements or analyses of a specified outcome |  Yes   Partial Yes   No   Includes only NRSI |
|  | **NRSI**  For Partial Yes, must have assessed RoB:   from confounding, *and*   from selection bias | For Yes, must also have assessed RoB:   methods used to ascertain exposures and outcomes, *and*   selection of the reported result from among multiple measurements or analyses of a specified outcome | 🗷 Yes   Partial Yes   No  Includes only RCTs |
| Comments: Used Newcastle‐Ottawa Scale and Egger test. | | | |

| **10. Did the review authors report outcomes on the sources of funding for the studies included in the review?** | | | | | | |
| --- | --- | --- | --- | --- | --- | --- |
| For Yes:   Must have reported on the sources of funding for individual studies included in the review.  Note: Reporting that the reviewers looked for this information but it was not reported by study authors also qualifies. | | | |  Yes  🗷 No |  |  |
| Comments: None | | | |  |  |  |
| **11. If meta-analysis was performed did the review authors use appropriate methods for statistical combination of results?** | | | | | | |
|  | **RCTs**  For Yes:   The authors justified combining the data in a meta-analysis   AND they used an appropriate weighted technique to combine study results and adjusted for heterogeneity if present.   AND investigated the causes of any heterogeneity | |  Yes   No   No meta-analysis conducted | | |  |
|  | **For NRSI**  For Yes:  🗷 The authors justified combining the data in a meta-analysis  🗷 AND they used an appropriate weighted technique to combine study results, adjusting for heterogeneity if present  🗷 AND they statistically combined effect estimates from NRSI that were adjusted for confounding, rather than combining raw data, or justified combining raw data when adjusted effect estimates were not available   AND they reported separate summary estimates for RCTs and NRSI separately when both were included in the review | | 🗷 Yes   No   No meta-analysis conducted | | |  |
|  | Comments: None | | | | |  |
| **12. If meta-analysis was performed, did the review authors assess the potential impact of RoB in individual studies on the results of the meta-analysis or other evidence synthesis?** | | | | | | |
|  | For Yes:   included only low risk of bias RCTs   OR, if the pooled estimate was based on RCTs and/or NRSI at variable RoB, the authors performed analyses to investigate possible impact of RoB on summary estimates of effect. | |  Yes  🗷 No   No meta-analysis conducted | | |  |
|  | Comments: None | | | | |  |
| **13. Did the review authors account for RoB in individual studies when interpreting/ discussing the results of the review?** | | | | | | |
|  | For Yes:   included only low risk of bias RCTs  🗷 OR, if RCTs with moderate or high RoB, or NRSI were included the review provided a discussion of the likely impact of RoB on the results | | 🗷 Yes   No | | |  |
|  | Comments: None | | | | |  |
| **14. Did the review authors provide a satisfactory explanation for, and discussion of, any heterogeneity observed in the results of the review?** | | | | | | |
|  | For Yes:   There was no significant heterogeneity in the results  🗷 OR if heterogeneity was present the authors performed an investigation of sources of any heterogeneity in the results and discussed the impact of this on the results of the review | | 🗷 Yes   No | | |  |
|  | Comments: None | | | | |  |
| **15. If they performed quantitative synthesis did the review authors carry out an adequate investigation of publication bias (small study bias) and discuss its likely impact on the results of the review?** | | | | | | |
|  | For Yes:  🗷 performed graphical or statistical tests for publication bias and discussed the likelihood and magnitude of impact of publication bias | | 🗷 Yes   No   No meta-analysis conducted | | |  |
|  | Comments: Used Egger test. | | | | |  |
| **16. Did the review authors report any potential sources of conflict of interest, including any funding they received for conducting the review?** | | | | | | |
|  | For Yes:  🗷 The authors reported no competing interests OR   The authors described their funding sources and how they managed potential conflicts of interest | 🗷 Yes   No | | | |  |
|  | Comments: None | | | | |  |

**To cite this tool:** Shea BJ, Reeves BC, Wells G, Thuku M, Hamel C, Moran J, Moher D, Tugwell P, Welch V, Kristjansson E, Henry DA. AMSTAR 2: a critical appraisal tool for systematic reviews that include randomised or non-randomised studies of healthcare interventions, or both. BMJ. 2017 Sep 21;358:j4008.

# Yang et al.

| **1. Did the research questions and inclusion criteria for the review include the components of PICO?** | | | | | |
| --- | --- | --- | --- | --- | --- |
| For Yes:   🗷 Population  n/a Intervention  n/a Comparator group  🗷 Outcome | | Optional (recommended)   Timeframe for follow-up | 🗷   | Yes  No |  |
|  Comments: Since this SR was of case studies, we considered that Outcomes were morbidities. They compared severe and non-severe patients. We considered that I-intervention and C-Comparator group were not applicable since this was not a review of studies of interventions. | | | | |  |
| **2. Did the report of the review contain an explicit statement that the review methods were established prior to the conduct of the review and did the report justify any significant deviations from the protocol?** | | | | | |
|  | For Partial Yes:  The authors state that they had a written protocol or guide that included ALL the following:   review question(s)   a search strategy   inclusion/exclusion criteria   a risk of bias assessment | For Yes:  As for partial yes, plus the protocol should be registered and should also have specified:   a meta-analysis/synthesis plan, if appropriate, *and*   a plan for investigating causes of heterogeneity   justification for any deviations from the protocol |     🗷 | Yes Partial Yes No |  |
|  | Comments: None | | | |  |
| **3. Did the review authors explain their selection of the study designs for inclusion in the review?** | | | | | |
|  | For Yes, the review should satisfy ONE of the following:   *Explanation for* including only RCTs   OR *Explanation for* including only NRSI   OR *Explanation for* including both RCTs and NRSI | | 🗷 | Yes No |  |
|  | Comments: They explained that they were interested in the epidemiological and clinical features of COVID-19 patients and searched for studies that addressed these issues. They did not put limits on their search. | | | |  |
| **4. Did the review authors use a comprehensive literature search strategy?** | | | | | |
|  | For Partial Yes (all the following): | For Yes, should also have (all the following):  🗷 searched the reference lists / bibliographies of included studies   searched trial/study registries   included/consulted content experts in the field   where relevant, searched for grey literature   conducted search within 24 months of completion of the review |  |  |  |
|  | 🗷 searched at least 2 databases (relevant to research question)  🗷 provided key word and/or search strategy  🗷 justified publication restrictions |  |   🗷 | Yes Partial Yes No |  |
|  | (e.g. language) |  |  |  |  |
|  | Comments: No language restriction. | | | |  |
|  | **5. Did the review authors perform study selection in duplicate?** | |  | |  |
|  | For Yes, either ONE of the following:   at least two reviewers independently agreed on selection of eligible studies and achieved consensus on which studies to include   OR two reviewers selected a sample of eligible studies and achieved good agreement (at least 80 percent), with the remainder selected by one reviewer. | |   🗷 | Yes No |  |
|  | Comments: State 2 investigators did the search, but not if they selected studies independently. | | | |  |

| **6. Did the review authors perform data extraction in duplicate?** | | | |
| --- | --- | --- | --- |
| For Yes, either ONE of the following:  🗷 at least two reviewers achieved consensus on which data to extract from included studies   OR two reviewers extracted data from a sample of eligible studies and achieved good agreement (at least 80 percent), with the remainder extracted by one reviewer. | | | 🗷 Yes   No |
| Comments: None | | | |
| **7. Did the review authors provide a list of excluded studies and justify the exclusions?** | | | |
|  | For Partial Yes:   provided a list of all potentially relevant studies that were read in full-text form but excluded from the review | For Yes, must also have:   Justified the exclusion from the review of each potentially relevant study |  Yes   Partial Yes  🗷 No |
|  | Comments: None | | |
| **8. Did the review authors describe the included studies in adequate detail?** | | | |
|  | For Partial Yes (ALL the following):  🗷 described populations  n/a described interventions  n/a described comparators  🗷 described outcomes   described research designs | For Yes, should also have ALL the following:  🗷 described population in detail  n/a described intervention in detail (including doses where relevant)  n/a described comparator in detail (including doses where relevant)  🗷 described study’s setting   timeframe for follow-up |  Yes   Partial Yes  🗷 No |
|  | Comments: No description of the research designs given, other than that they described the clinical characteristics of the patients. | | |
| **9. Did the review authors use a satisfactory technique for assessing the risk of bias (RoB) in individual studies that were included in the review?** | | | |
|  | **RCTs**  For Partial Yes, must have assessed RoB from   unconcealed allocation, *and*   lack of blinding of patients and assessors when assessing outcomes (unnecessary for objective outcomes such as all-cause mortality) | For Yes, must also have assessed RoB from:   allocation sequence that was not truly random, *and*   selection of the reported result from among multiple measurements or analyses of a specified outcome |  Yes   Partial Yes   No   Includes only NRSI |
|  | **NRSI**  For Partial Yes, must have assessed RoB:   from confounding, *and*   from selection bias | For Yes, must also have assessed RoB:   methods used to ascertain exposures and outcomes, *and*   selection of the reported result from among multiple measurements or analyses of a specified outcome |  Yes   Partial Yes  🗷 No  Includes only RCTs |
| Comments: None | | | |

| **10. Did the review authors report outcomes on the sources of funding for the studies included in the review?** | | | | | | |
| --- | --- | --- | --- | --- | --- | --- |
| For Yes:   Must have reported on the sources of funding for individual studies included in the review.  Note: Reporting that the reviewers looked for this information but it was not reported by study authors also qualifies. | | | |  Yes  🗷 No |  |  |
| Comments: None | | | |  |  |  |
| **11. If meta-analysis was performed did the review authors use appropriate methods for statistical combination of results?** | | | | | | |
|  | **RCTs**  For Yes:   The authors justified combining the data in a meta-analysis   AND they used an appropriate weighted technique to combine study results and adjusted for heterogeneity if present.   AND investigated the causes of any heterogeneity | |  Yes   No   No meta-analysis conducted | | |  |
|  | **For NRSI**  For Yes:  🗷 The authors justified combining the data in a meta-analysis  🗷 AND they used an appropriate weighted technique to combine study results, adjusting for heterogeneity if present  🗷 AND they statistically combined effect estimates from NRSI that were adjusted for confounding, rather than combining raw data, or justified combining raw data when adjusted effect estimates were not available   AND they reported separate summary estimates for RCTs and NRSI separately when both were included in the review | | 🗷 Yes   No   No meta-analysis conducted | | |  |
|  | Comments: None | | | | |  |
| **12. If meta-analysis was performed, did the review authors assess the potential impact of RoB in individual studies on the results of the meta-analysis or other evidence synthesis?** | | | | | | |
|  | For Yes:   included only low risk of bias RCTs   OR, if the pooled estimate was based on RCTs and/or NRSI at variable RoB, the authors performed analyses to investigate possible impact of RoB on summary estimates of effect. | |  Yes  🗷 No   No meta-analysis conducted | | |  |
|  | Comments: None | | | | |  |
| **13. Did the review authors account for RoB in individual studies when interpreting/ discussing the results of the review?** | | | | | | |
|  | For Yes:   included only low risk of bias RCTs   OR, if RCTs with moderate or high RoB, or NRSI were included the review provided a discussion of the likely impact of RoB on the results | |  Yes  🗷 No | | |  |
|  | Comments: None | | | | |  |
| **14. Did the review authors provide a satisfactory explanation for, and discussion of, any heterogeneity observed in the results of the review?** | | | | | | |
|  | For Yes:   There was no significant heterogeneity in the results  🗷 OR if heterogeneity was present the authors performed an investigation of sources of any heterogeneity in the results and discussed the impact of this on the results of the review | | 🗷 Yes   No | | |  |
|  | Comments: None | | | | |  |
| **15. If they performed quantitative synthesis did the review authors carry out an adequate investigation of publication bias (small study bias) and discuss its likely impact on the results of the review?** | | | | | | |
|  | For Yes:   performed graphical or statistical tests for publication bias and discussed the likelihood and magnitude of impact of publication bias | |  Yes  🗷 No   No meta-analysis conducted | | |  |
|  | Comments: None | | | | |  |
| **16. Did the review authors report any potential sources of conflict of interest, including any funding they received for conducting the review?** | | | | | | |
|  | For Yes:  🗷 The authors reported no competing interests OR   The authors described their funding sources and how they managed potential conflicts of interest | 🗷 Yes   No | | | |  |
|  | Comments: None | | | | |  |

**To cite this tool:** Shea BJ, Reeves BC, Wells G, Thuku M, Hamel C, Moran J, Moher D, Tugwell P, Welch V, Kristjansson E, Henry DA. AMSTAR 2: a critical appraisal tool for systematic reviews that include randomised or non-randomised studies of healthcare interventions, or both. BMJ. 2017 Sep 21;358:j4008.
