# Supplementary material for: Coronavirus disease (COVID-19) pandemic: an overview of systematic reviews
Source: BMC Infect Dis. 2021 Jun 4;21:525. doi: 10.1186/s12879-021-06214-4 (PMC8177249; doi:10.1186/s12879-021-06214-4)

**Appendix 6. Member list – InterNetCOVID-19**


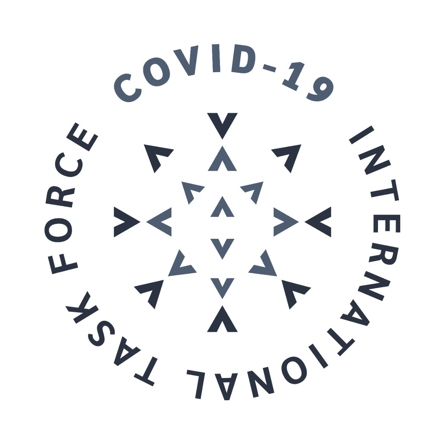


| **List of members involved to the International Network of Coronavirus Disease 2019 (InterNetCOVID-19)** | |
| --- | --- |
| **Core members** |  |
| **Brazil** |  |
| Israel Júnior Borges do Nascimento | University Hospital and School of Medicine, Universidade Federal de Minas Gerais, Belo Horizonte, Minas Gerais, 30130-100 Brazil. |
| Milena Soriano Marcolino | University Hospital and School of Medicine, Universidade Federal de Minas Gerais, Belo Horizonte, Minas Gerais, 30130-100 Brazil. |
| Silvana Mangeon Meirelles Guimarães | University Hospital and School of Medicine, Universidade Federal de Minas Gerais, Belo Horizonte, Minas Gerais, 30130-100 Brazil. |
| Vinicius Tassoni Civile | Cochrane Brazil; Evidence-Based Health Program, Universidade Federal de São Paulo, São Paulo, 04021-001 Brazil. |
| Nelson Canvas Junior | Cochrane Brazil; Universidade Paulista, São Paulo, 04057-000 Brazil. |
| **Germany** |  |
| Thilo von Groote | Department of Anesthesiology, Intensive Care and Pain Medicine, University of Münster, Münster, 48149 Germany. |
| Hebatullah Mohamed Abdulazeem | Department of Sport and Health Sciences, Technische Universität München, Munich, 80333 Germany. |
| **Australia** |  |
| Ishanka Weerasekara | School of Health Sciences, Faculty of Health and Medicine, The University of Newcastle, Callaghan, 2308 Australia |
| **Croatia** |  |
| Nensi Cacic | Cochrane Croatia, University of Split School of Medicine, Split, 21000 Croatia. |
| Ana Jeroncic | Cochrane Croatia, University of Split School of Medicine, Split, 21000 Croatia. |
| Tina Poklepovic Pericic | Cochrane Croatia, University of Split School of Medicine, Split, 21000 Croatia. |
| Ana Marusic | Cochrane Croatia, University of Split School of Medicine, Split, 21000 Croatia. |
| Irena Zakarija-Grkovic | Cochrane Croatia, University of Split School of Medicine, Split, 21000 Croatia. |
| 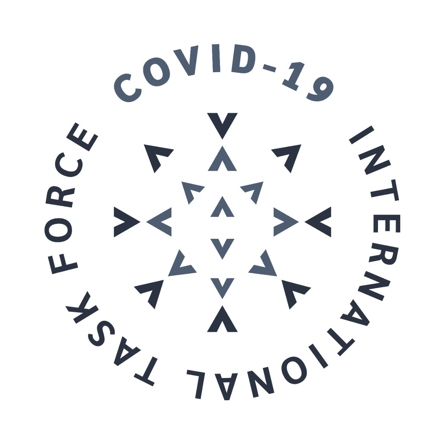  **Canada** |  |
| Nicola Roberto Bragazzi | Laboratory for Industrial and Applied Mathematics (LIAM), Department of Mathematics and Statistics, York University, Toronto, Ontario, M3J 1P3 Canada |
| **Sri Lanka** |  |
| Umesh Jayarajah | Department of Surgery, Faculty of Medicine, University of Colombo, Colombo, 00700 Sri Lanka. |
| **United States of America** |  |
| Dónal P. O’Mathúna | Helene Fuld Health Trust National Institute for Evidence-based Practice in Nursing and Healthcare, College of Nursing, The Ohio State University, Columbus, Ohio, 43210 United States of America |
| **Sweden** |  |
| Maria Bjorklund | Faculty of Medicine, Lund University, Lund, SE-221-00, Sweden |
| **Affiliate Members** |  |
| Catherine Henderson | Swanscoe Communications, Bollington, Macclesfield, SK10 5JH United Kingdom. |
| Livia Puljak | Center for Evidence-Based Medicine and Healthcare, Catholic University of Croatia, Ilica 242, 10000, Zagreb, Croatia. |
| Meisam Abdar Esfahani | Cochrane Iran Associate Centre, National Institute for Medical Research Development, Tehran, 16846 Iran |
| Ahmad Sofi-Mahmudi | Cochrane Iran Associate Centre, National Institute for Medical Research Development, Tehran, 16846 Iran |
| Mohammad Altujjar | Department of Internal Medicine, University of Toledo, Toledo, Ohio, 43606 United States of America |
| Nathalia Sernizon Guimarães | University Hospital and School of Medicine, Universidade Federal de Minas Gerais, Belo Horizonte, Minas Gerais, 30130-100 Brazil. |
| Maoyi Tian | The George Institute for Global Health, University of New South Wales, Sydney, New South Wales, 2052 Australia |
| Diana Maria Cespedes Arcani | Department of Cardiovascular and Thoracic Surgery, Zhongnan Hospital, Wuhan University, Hubei, China |
| Alvaro Nagib Attallah | Cochrane Brazil; Evidence-Based Health Program, Universidade Federal de São Paulo, São Paulo, 04021-001 Brazil. |
| Abhijna Vithal Yergolkar | Department of Pharmacy Practice, M. S. Ramaiah University of Applied Sciences, Bangalore, India |
| Joanna Przeździecka-Dołyk | Department and Clinic of Ophthalmology, Medical University of Wroclaw, Wroclaw, Poland |
| Santino Filoso | Yorkville University, Fredericton, New Brunswick, Canada |
| Ingrid Ellen Herculano dos Santos | Department of Graphic Design, Universidade Federal de Campina Grande, Campina Grande, Paraiba, Brazil |
| Bruna Diniz | Department of Marketing and Propaganda, UNIESP, Paraíba, Brazil |
| Cristina Riboni | Paediatric Surgery Department, San Matteo Hospital, Pavia, Italy |
| Henning Klapproth | Department of Internal Medicine, University of Münster, Münster, 48149 Germany. |
| Katiane Cunha | Faculty of Medicine, Universidade Estadual do Para, Maraíba, Pará, Brazil |
| Luanna Monteiro | University Hospital and School of Medicine, Universidade Federal de Minas Gerais, Belo Horizonte, Minas Gerais, Brazil. |
| Maria de Fátima Leite | Laboratório de Sinalização de Cálcio, Instituto de Ciências Biológicas, Universidade Federal de Minas Gerais, Belo Horizonte, Brazil |
| Michael Nathanson | Liver Clinic and Yale Digestive Diseases, Yale School of Medicine, New Haven, Connecticut, United States of America |
| Valéria Alves Fernandes | University Hospital and School of Medicine, Universidade Federal de Minas Gerais, Belo Horizonte, Minas Gerais, Brazil. |


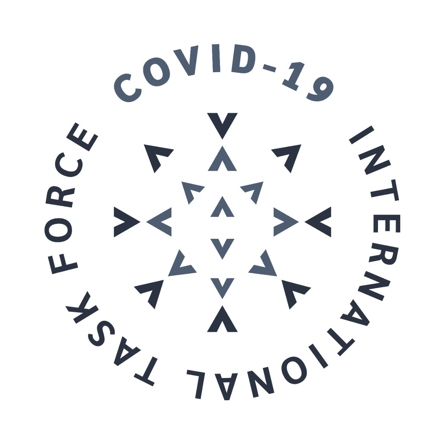

Supplement: Supplementary file 6 — Additional file 6: Appendix 6. List of members and affiliates of International Network of Coronavirus Disease 2019 (InterNetCOVID-19). [file 12879_2021_6214_MOESM6_ESM.docx]
